# Supplementary figures and images for: Seizure evolution in a mouse model of West syndrome involves complex and time-dependent synapse remodeling, gliosis and alterations in lipid metabolism
Source: PLoS Biol. 2025 Oct 9;23(10):e3003192. doi: 10.1371/journal.pbio.3003192 (PMC12520403; doi:10.1371/journal.pbio.3003192)

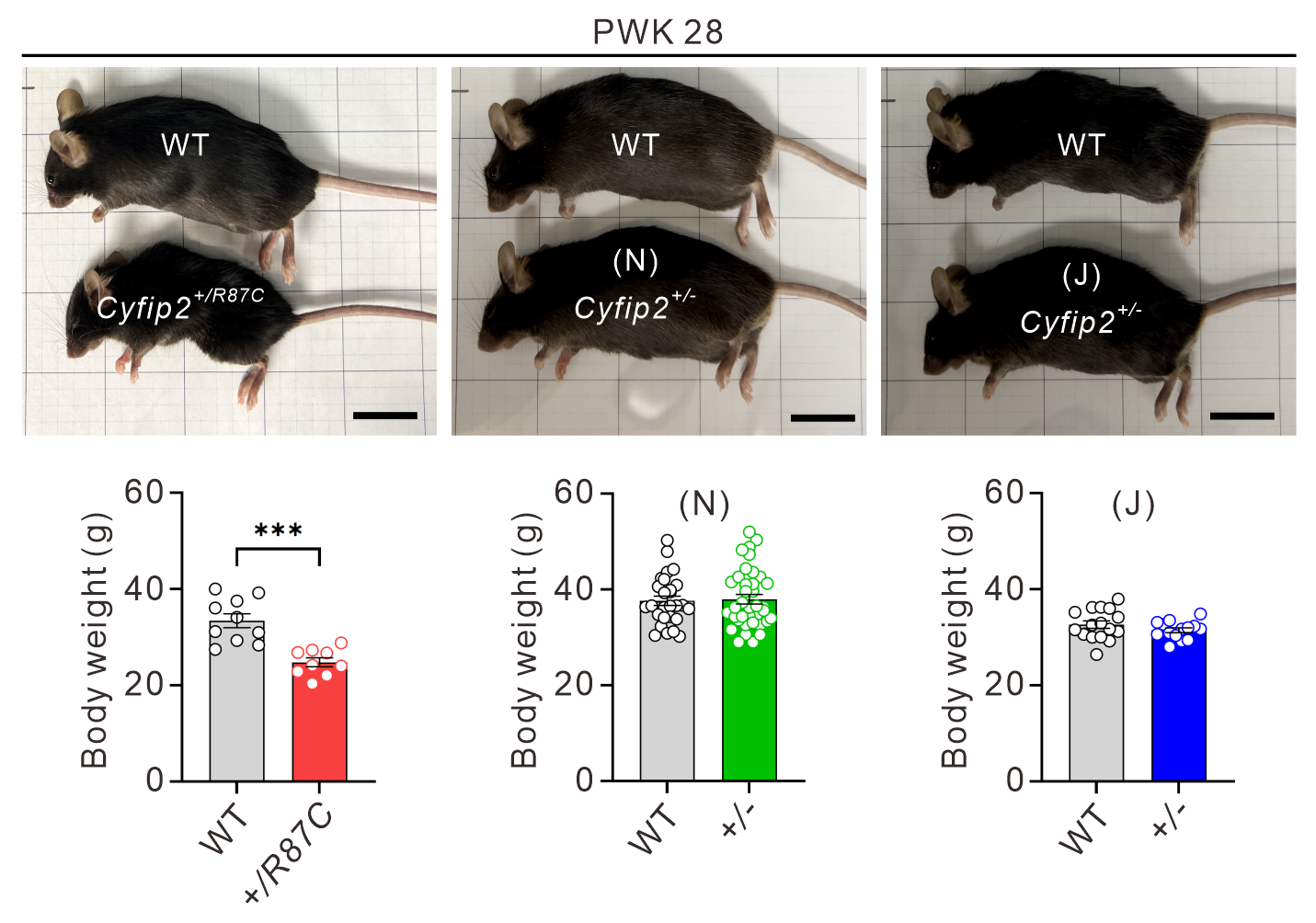

Supplement: S1 Fig — Scale bar, 2 cm (n = 9–32 mice per genotype, unpaired two-tailed Student t test). N = C57BL/6N background, J = C57BL/6J background. ***P < 0.001. Data are represented as mean ± standard error of the mean. The data underlying this Figure can be found in S1 Data. (TIF) [file pbio.3003192.s001.tif]

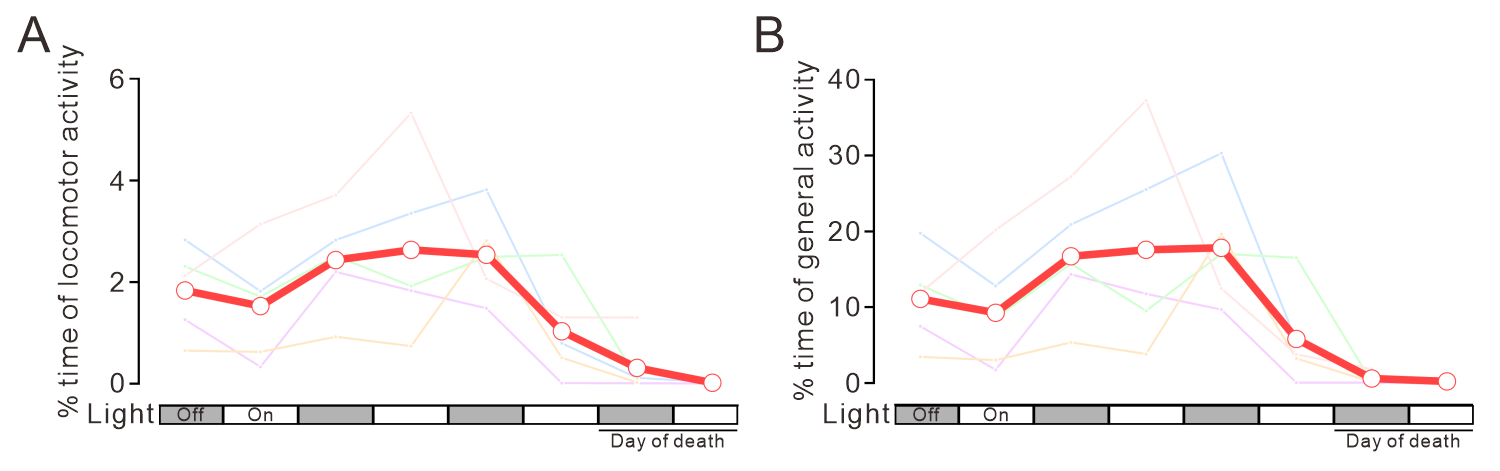

Supplement: S2 Fig — (A) Percentage of time spent in locomotor activity for five Cyfip2+/R87C mice (thin lines) and their average (thick red line) over the final 3–4 days before death. On the day of death, the exact time of death differed among individual mice. (B) Percentage of time spent in general activity of Cyfip2+/R87C mice during the final 3–4 days before death. The data underlying this Figure can be found in S1 Data. (TIF) [file pbio.3003192.s002.tif]

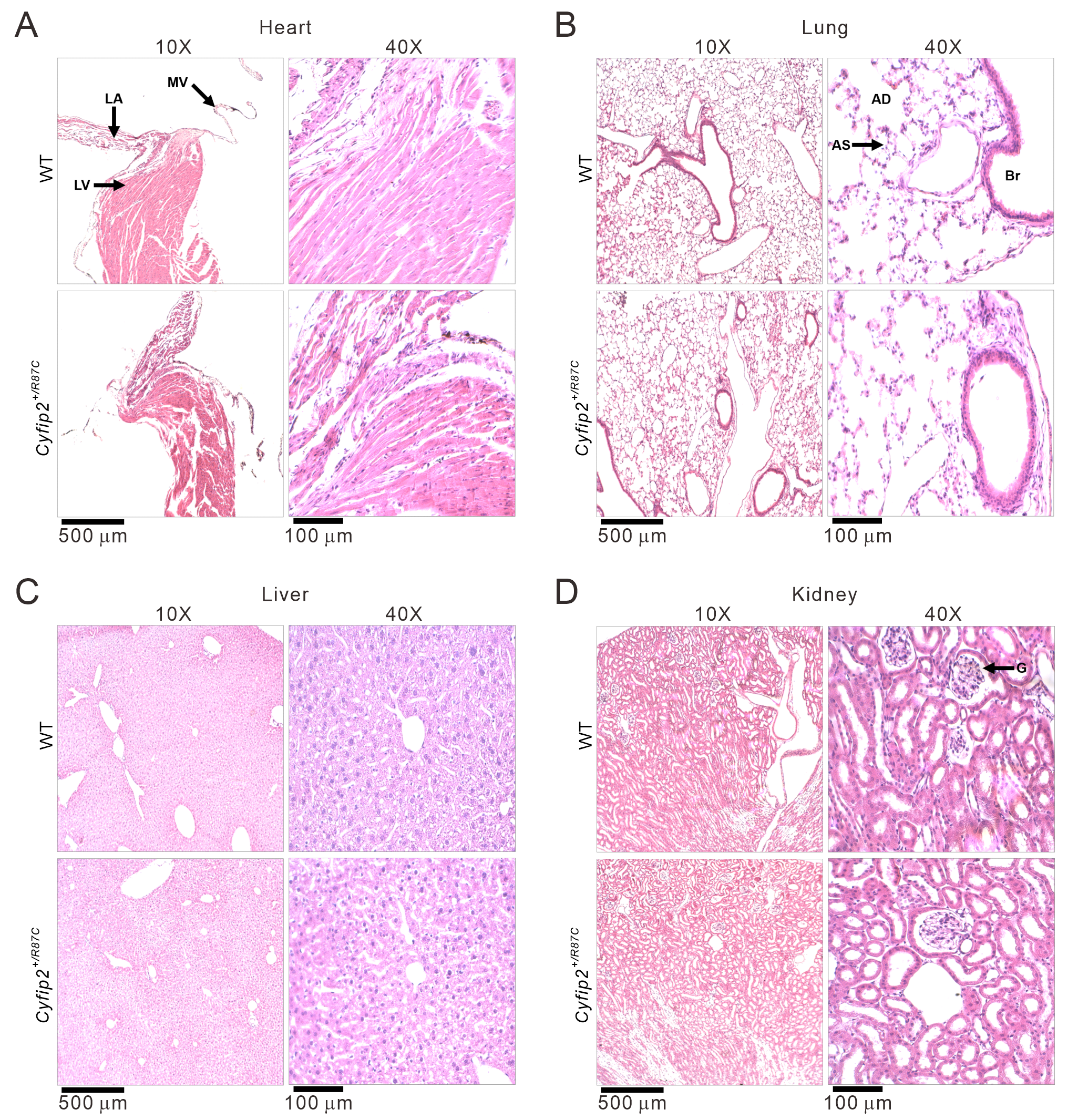

Supplement: S3 Fig — (A) Representative histological images of WT (upper panels) and Cyfip2+/R87C (lower panels) mouse hearts show intact myocardial architecture with well-organized cardiac muscle fibers and normal myocyte morphology in both genotypes. The left ventricular (LV) and atrial (LA) walls, mitral valve (MV), and intramyocardial vessels appear unremarkable. No pathological changes, including but not limited to, inflammatory cell infiltration, cardiomyocyte hypertrophy or degeneration, fibrosis, necrosis, calcification, or valve thickening, are observed in either WT or Cyfip2+/R87C mice. Scale bars, 500 µm (10× magnification, left panels) and 100 µm (40× magnification, right panels). (B) Representative histological images of lungs show normal pulmonary architecture in both genotypes. Alveolar spaces are well-expanded with thin alveolar septa (AS), and bronchioles (Br) and alveolar ducts (AD) appear unremarkable. No pathological changes such as infection, inflammation, hemorrhage, septal thickening, edema, or fibrosis are observed in either genotype. (C) Representative liver histology shows well-preserved hepatic architecture in both genotypes. Hepatocytes are arranged in uniform plates radiating from central veins, with clear cytoplasm and round, centrally located nuclei. No pathological changes such as inflammatory cell infiltration, steatosis, hepatocellular degeneration, necrosis, fibrosis, or cholestasis are observed in either genotype. (D) Representative kidney histology shows normal renal architecture in both genotypes. The cortex exhibits well-defined glomeruli (G) with intact capillary loops and surrounding Bowman’s capsules, along with normal proximal and distal tubules. The medulla shows preserved tubular structures and collecting ducts without signs of congestion or degeneration. No pathological changes such as glomerular hypercellularity, tubular atrophy, interstitial inflammation, fibrosis, or vascular abnormalities are observed in either genotype. (TIF) [file pbio.3003192.s003.tif]

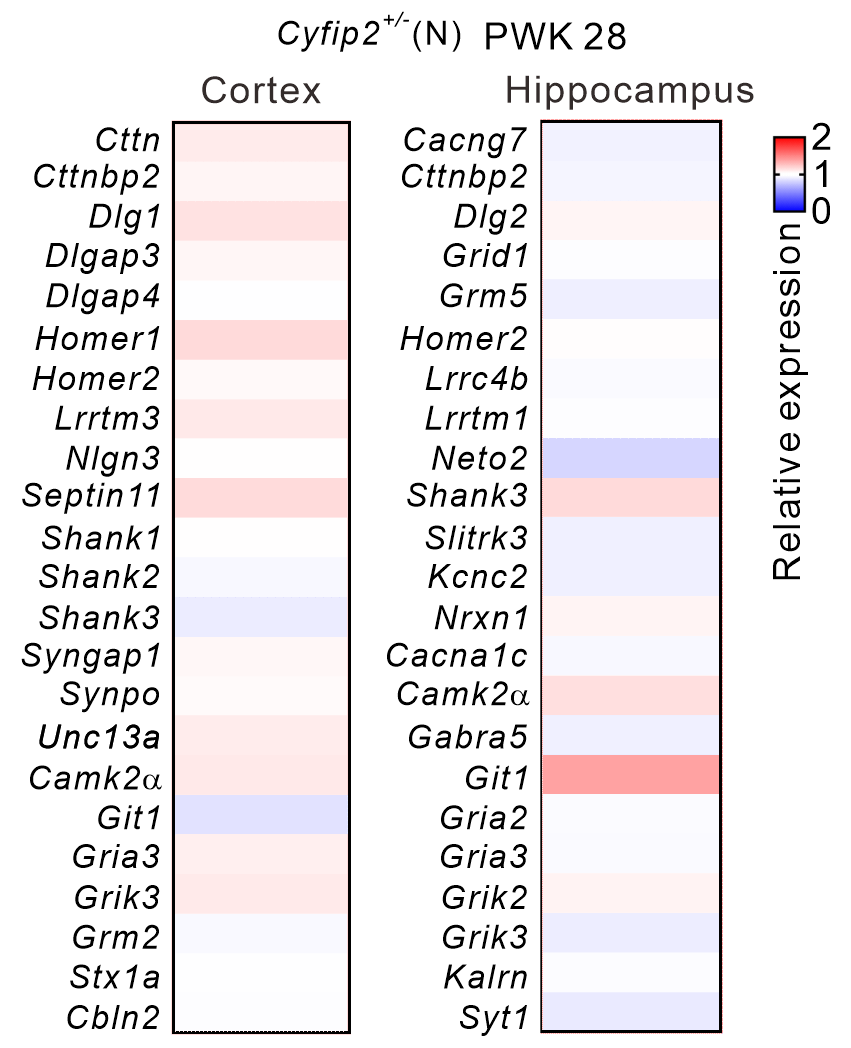

Supplement: S4 Fig — No significant differences were observed between genotypes (n = 6 mice per genotype, unpaired two-tailed Student t test). N = C57BL/6N background. The data underlying this Figure can be found in S1 Data. (TIF) [file pbio.3003192.s004.tif]

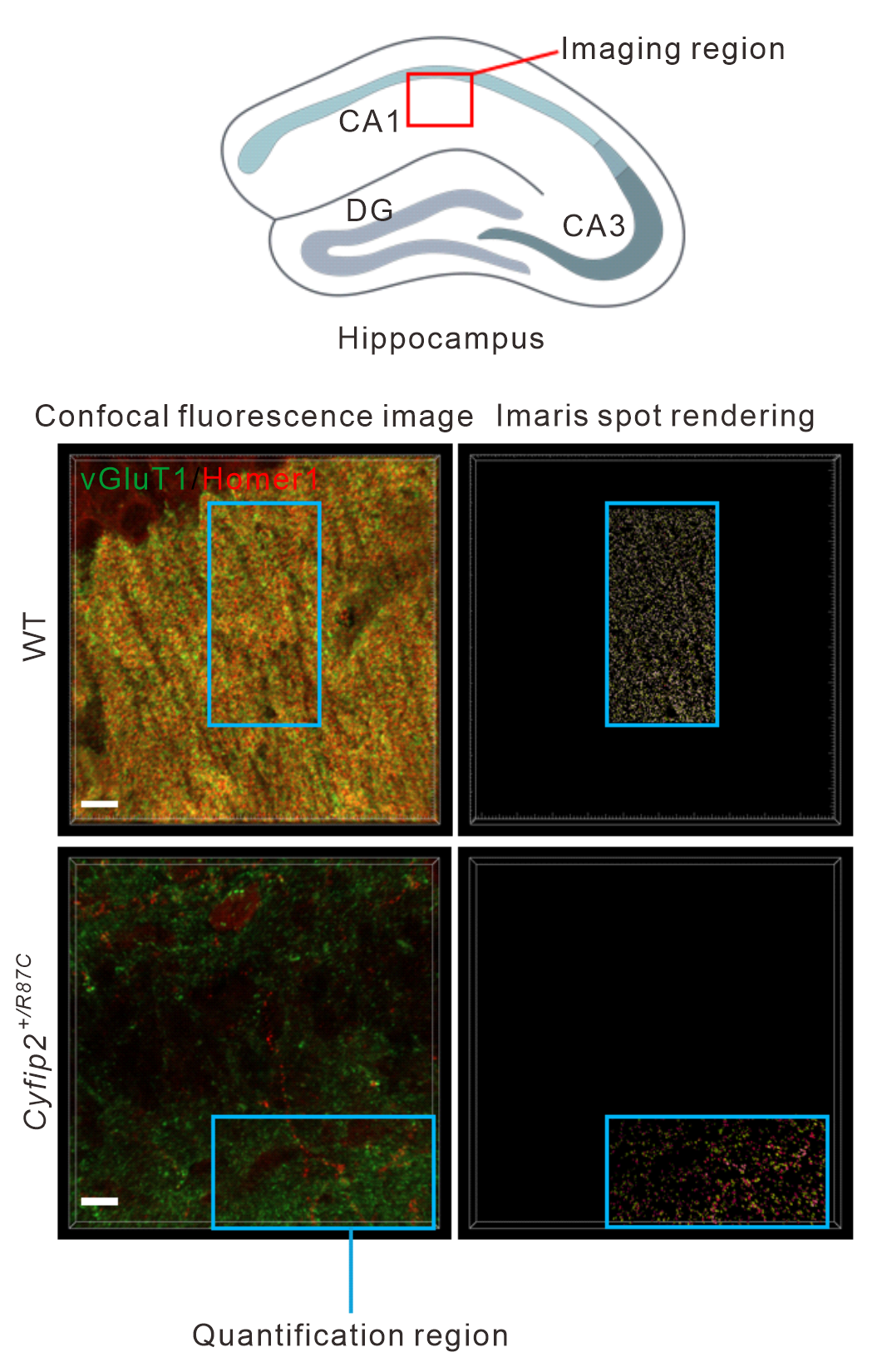

Supplement: S5 Fig — A schematic diagram illustrating the hippocampal CA1 region, where the confocal images were acquired. The images on the bottom show examples of converting the hippocampal confocal fluorescence images into spot images using Imaris rendering for WT and Cyfip2+/R87C mice. Scale bar, 10 μm. (TIF) [file pbio.3003192.s005.tif]

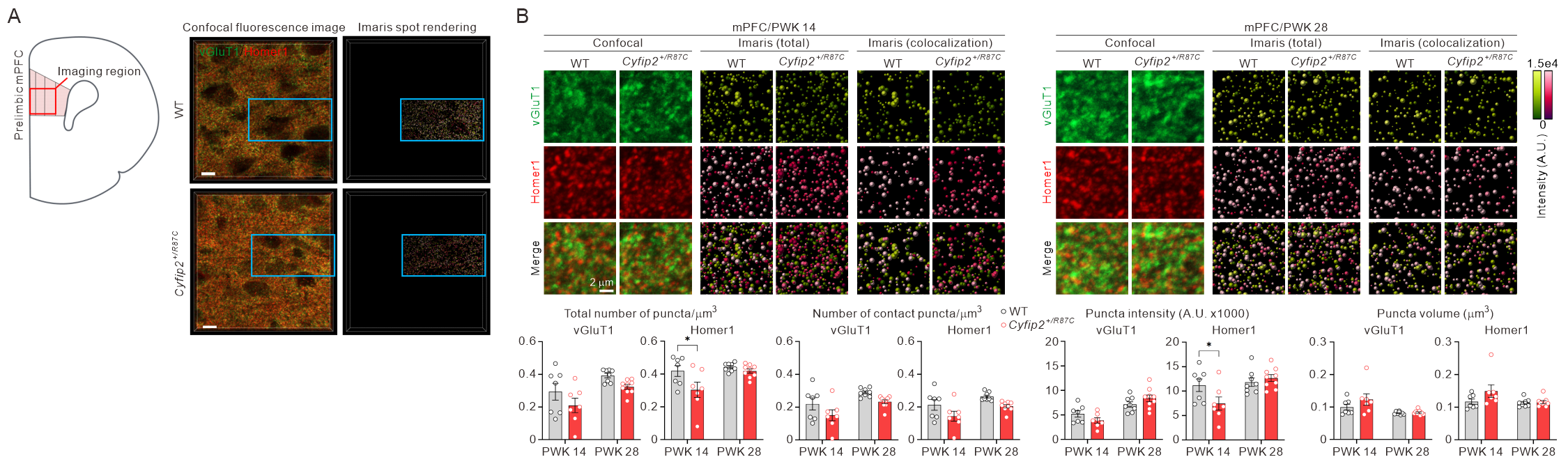

Supplement: S6 Fig — (A) Schematic diagram illustrating the prelimbic medial prefrontal cortex (mPFC), where the confocal images were acquired. The images on the right show examples of converting the mPFC confocal fluorescence images into spot images using Imaris rendering for WT and Cyfip2+/R87C mice. Scale bar, 10 μm. (B) Representative fluorescence immunohistochemistry images and quantification show changes in excitatory presynaptic (vGluT1) and postsynaptic (Homer1) markers in the prelimbic mPFC of Cyfip2+/R87C mice compared to WT mice at PWK 14 and 28 (n = 7–9 mice per genotype, two-way ANOVA with Šídák’s multiple comparisons test). Images for automated foci counting, obtained using Imaris software, are also shown. A.U. = arbitrary units. *P < 0.05. Data are represented as mean ± standard error of the mean. The data underlying this Figure can be found in S1 Data. (TIF) [file pbio.3003192.s006.tif]

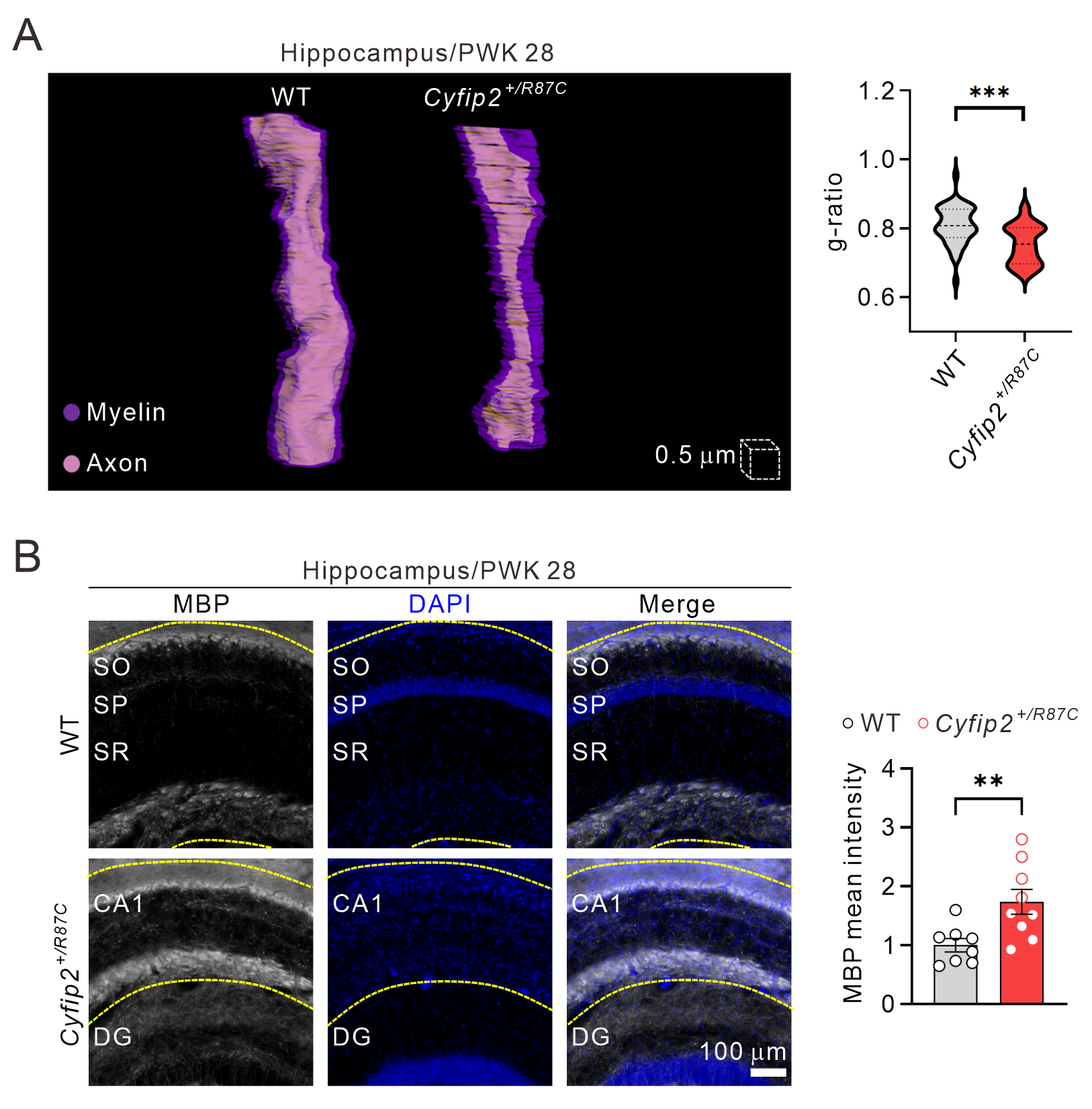

Supplement: S7 Fig — (A) Representative 3D electron microscopy images of axonal segments in the hippocampal CA1 region from WT and Cyfip2+/R87C mice at PWK 28. The graph displays the g-ratio (axon diameter/total myelinated fiber diameter) for WT and Cyfip2+/R87C mice (n = 36–41 axons per genotype, unpaired two-tailed Student t test). (B) Representative fluorescence immunohistochemistry images and corresponding quantification show increased myelin basic protein (MBP) mean intensity in the hippocampus of Cyfip2+/R87C mice compared to WT mice at PWK 28 (n = 8–9 mice per genotype, unpaired two-tailed Student t test). DG = dentate gyrus, SO = stratum oriens, SP = stratum pyramidale, SR = stratum radiatum. **P < 0.01; ***P < 0.001. Data are represented as mean ± standard error of the mean. The data underlying this Figure can be found in S1 Data. (TIF) [file pbio.3003192.s007.tif]

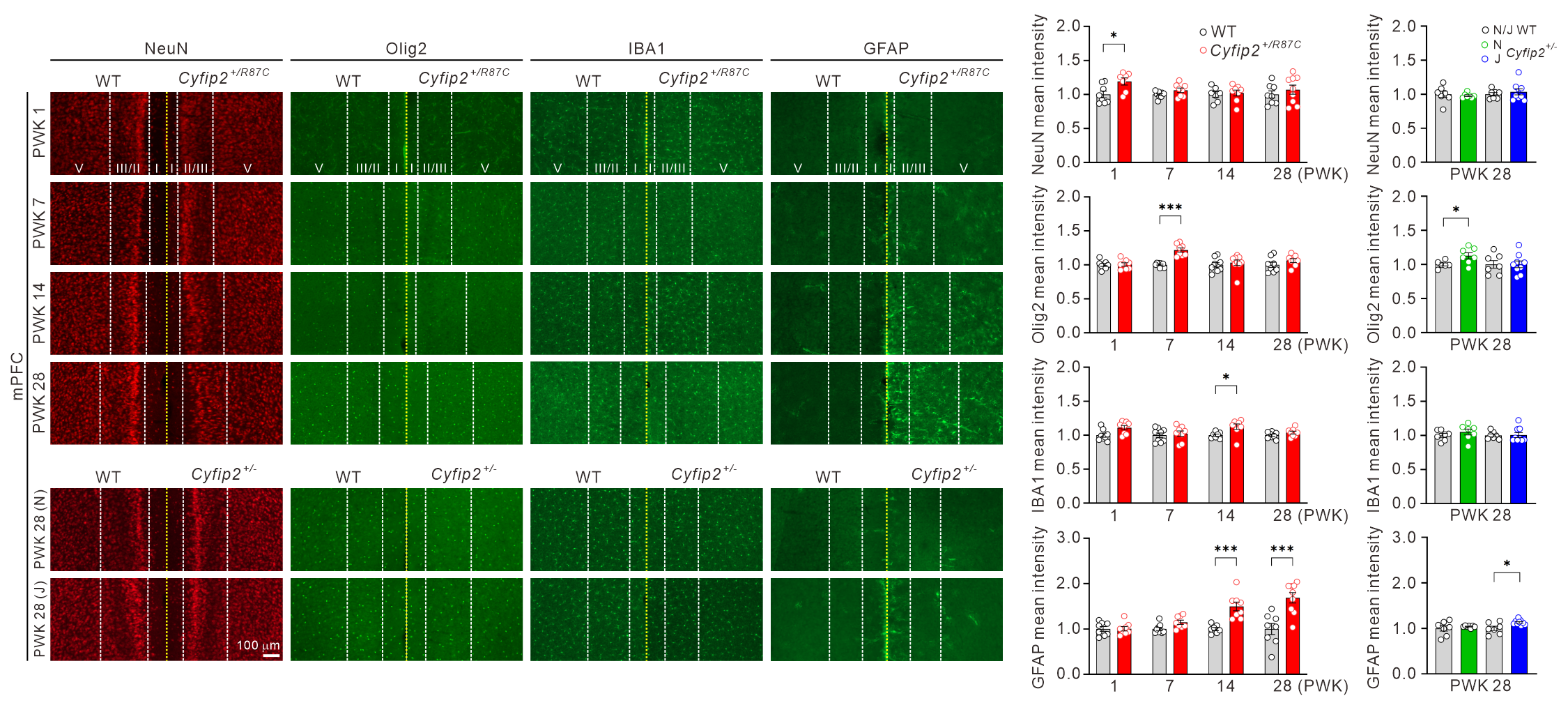

Supplement: S8 Fig — Representative fluorescence immunohistochemistry images and quantifications show age-dependent changes in the intensities of neuronal and glial cell markers in the prelimbic medial prefrontal cortex (mPFC) of Cyfip2+/R87C mice compared to WT mice (n = 7–9 mice per genotype, two-way ANOVA with Šídák’s multiple comparisons test). The results of the same analysis for Cyfip2+/− mice (on either the C57BL/6N (N) or C57BL/6J (J) backgrounds) at PWK 28 are also presented (n = 6–9 mice per genotype, two-way ANOVA with Šídák’s multiple comparisons test). *P < 0.05; ***P < 0.001. Data are represented as mean ± standard error of the mean. The data underlying this Figure can be found in S1 Data. (TIF) [file pbio.3003192.s008.tif]

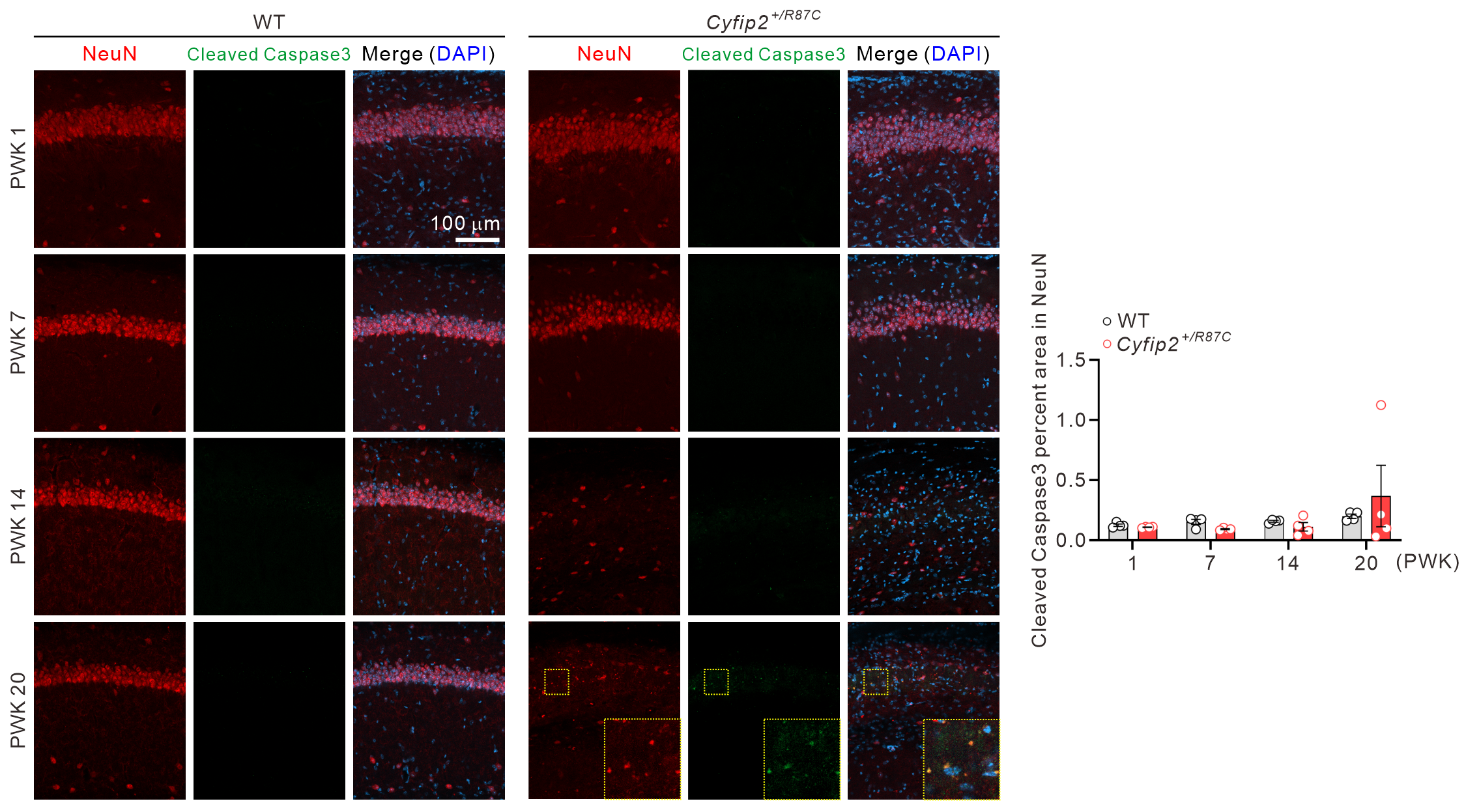

Supplement: S9 Fig — Co-staining for NeuN and cleaved Caspase3 was performed at PWK 1, 7, 14, and 20. Quantitative analysis of the fraction of cleaved Caspase3-positive area within the NeuN-positive region showed no significant differences between WT and Cyfip2+/R87C mice. At PWK 20, however, small NeuN- and cleaved Caspase3-double-positive spots were frequently observed in Cyfip2+/R87C mice but not in WT mice, potentially representing debris from dead NeuN-positive neurons. The data underlying this Figure can be found in S1 Data. (TIF) [file pbio.3003192.s009.tif]

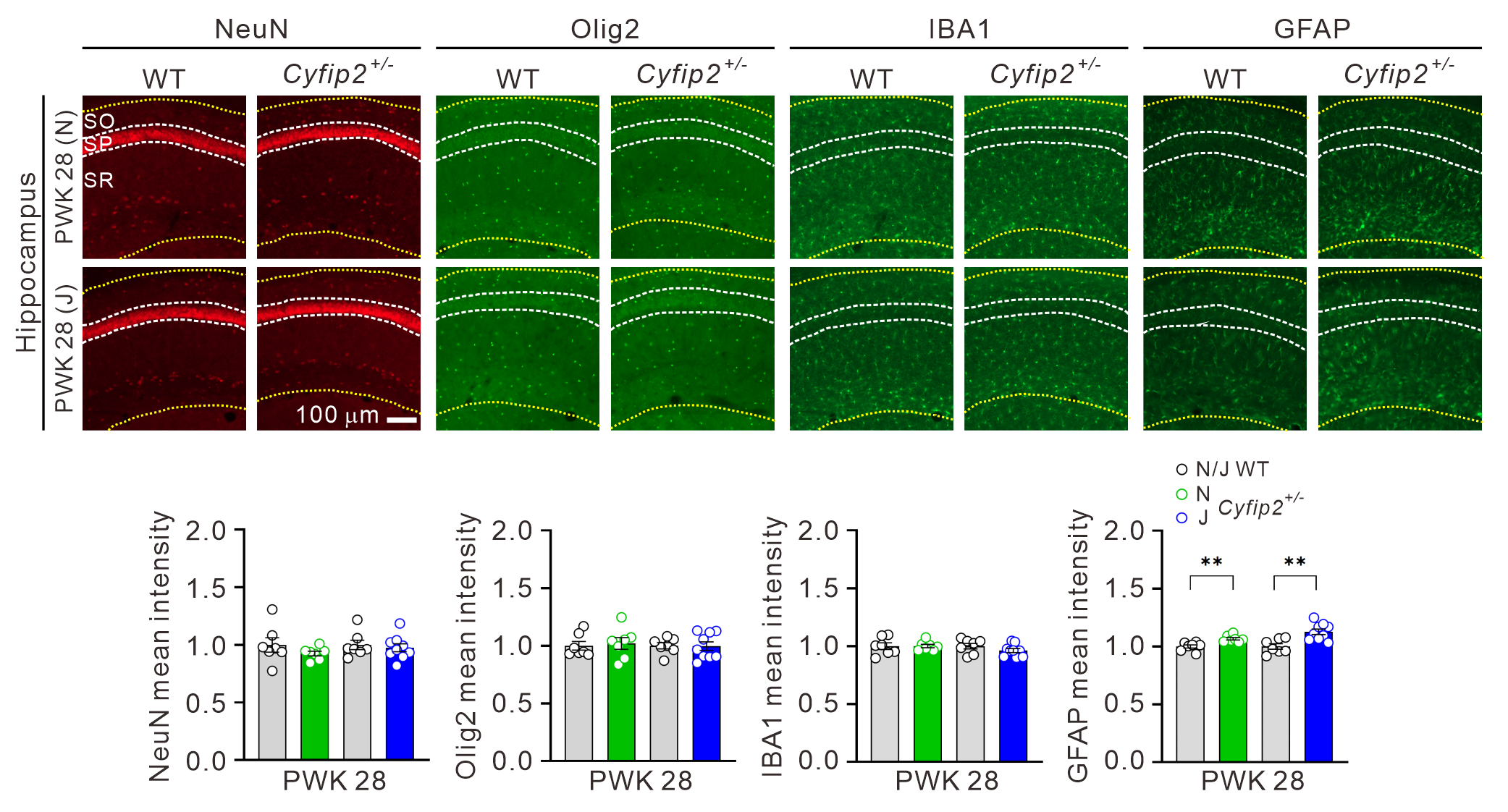

Supplement: S10 Fig — Changes in the intensities of neuronal and glial cell markers in the hippocampal CA1 region of Cyfip2+/− mice at PWK 28 (n = 7–9 mice per genotype, two-way ANOVA with Šídák’s multiple comparisons test). SO = stratum oriens, SP = stratum pyramidale, SR = stratum radiatum. **P < 0.01. Data are represented as mean ± standard error of the mean. The data underlying this Figure can be found in S1 Data. (TIF) [file pbio.3003192.s010.tif]

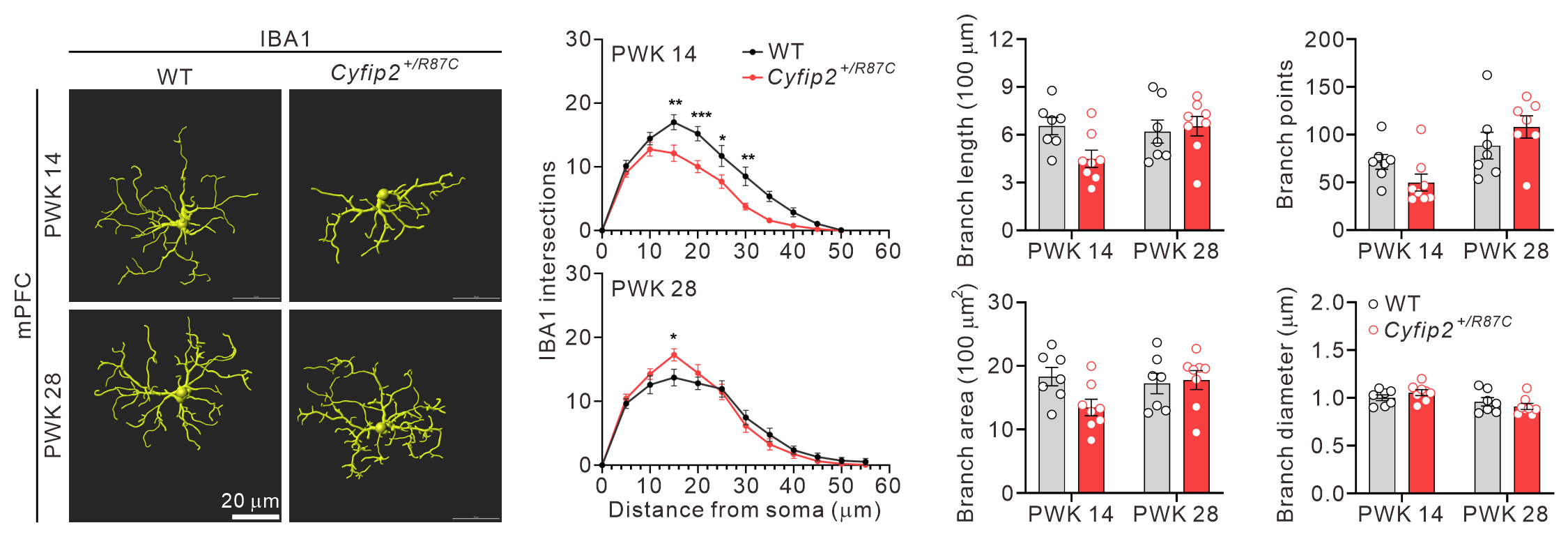

Supplement: S11 Fig — Representative 3D images of microglia in the prelimbic mPFC of WT and Cyfip2+/R87C mice at PWK 14 and 28, processed using Imaris software. Graphs show quantifications from Sholl analysis as well as measurements of branch length, area, points, and diameter (n = 7–8 mice per genotype, two-way ANOVA with Šídák’s multiple comparisons test). *P < 0.05; **P < 0.01; ***P < 0.001. Data are represented as mean ± standard error of the mean. The data underlying this Figure can be found in S1 Data. (TIF) [file pbio.3003192.s011.tif]

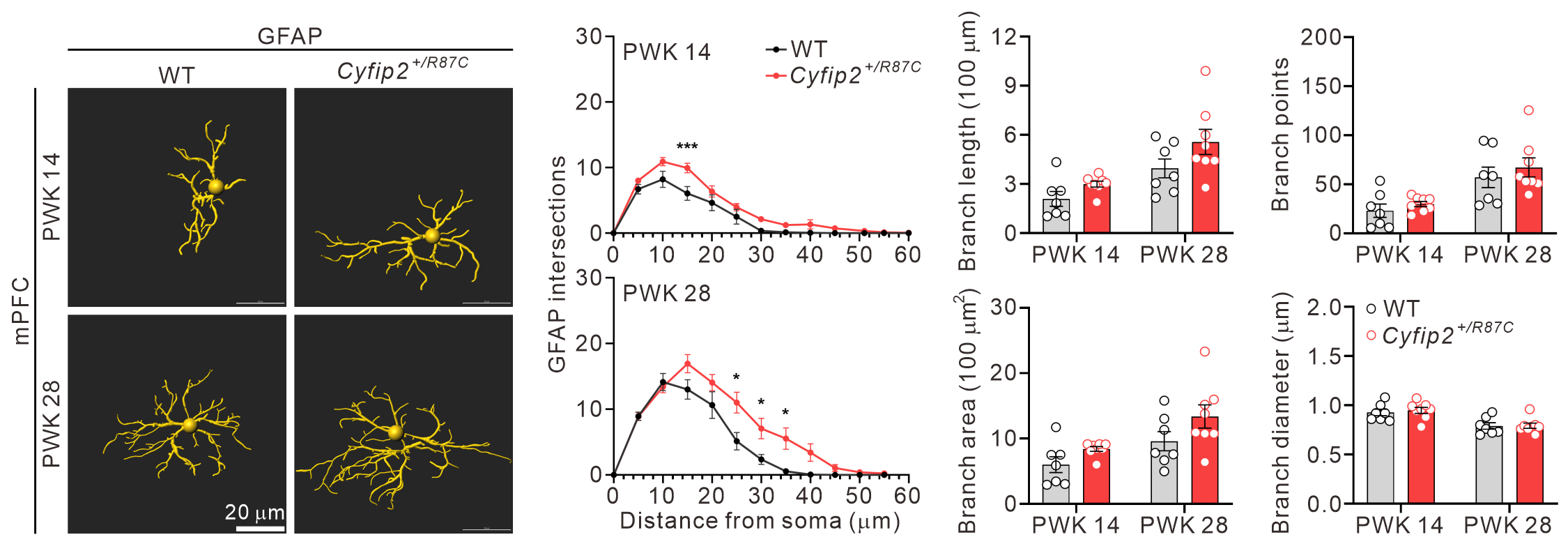

Supplement: S12 Fig — Representative 3D images of astrocytes in the mPFC prelimbic region of WT and Cyfip2+/R87C mice at PWK 14 and 28, processed using Imaris software. Graphs show quantifications from Sholl analysis as well as measurements of branch length, area, points, and diameter (n = 7–8 mice per genotype, two-way ANOVA with Šídák’s multiple comparisons test). *P < 0.05; ***P < 0.001. Data are represented as mean ± standard error of the mean. The data underlying this Figure can be found in S1 Data. (TIF) [file pbio.3003192.s012.tif]

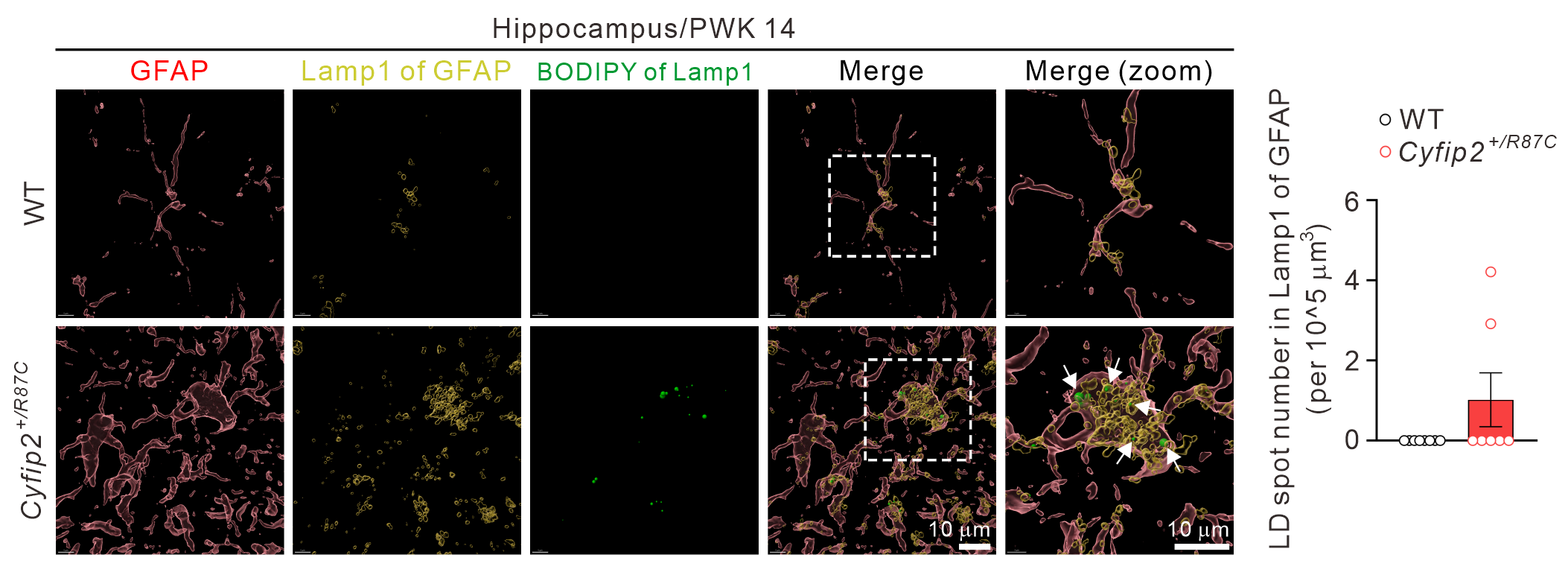

Supplement: S13 Fig — Representative 3D images show astrocytes (GFAP), lysosome (Lamp1) within the astrocytes, and lipid droplets (LDs, BODIPY) within astrocytic lysosome (indicated by white arrows in merged images) in the hippocampal CA1 region of WT and Cyfip2+/R87C mice at PWK 14, analyzed using Imaris software. The Graph shows quantification of the number of LDs within astrocytic lysosomes in WT and Cyfip2+/R87C mice (n = 7–8 mice per genotype, unpaired two-tailed Student t test). The data underlying this Figure can be found in S1 Data. (TIF) [file pbio.3003192.s013.tif]

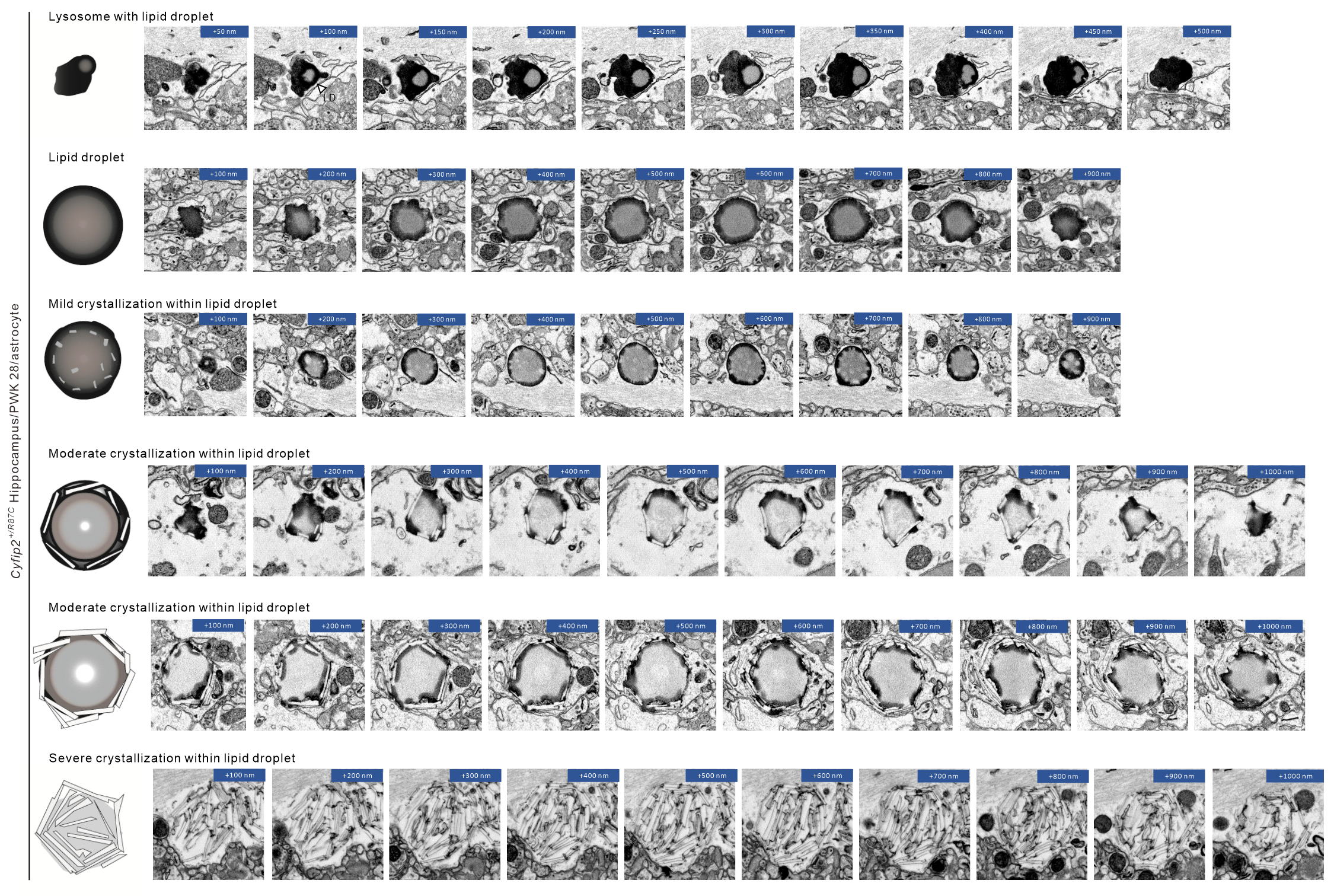

Supplement: S14 Fig — Serial electron microscopic images of various types of lipid droplets (within lysosome, with or without crystals) in the hippocampal astrocytes of Cyfip2+/R87C mice at PWK 28. Schematic diagrams for each type of lipid droplet are provided on the left side. (TIF) [file pbio.3003192.s014.tif]

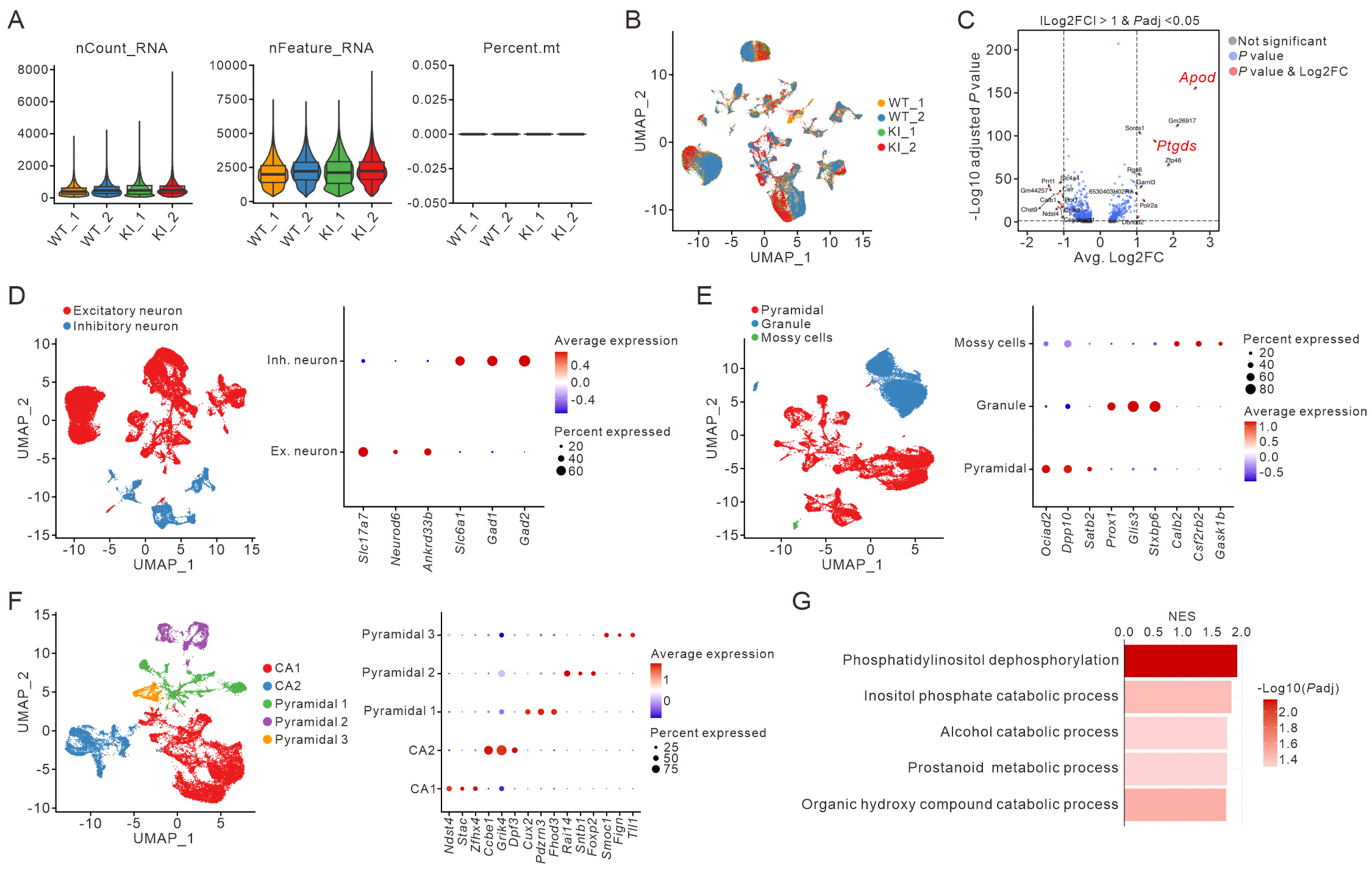

Supplement: S15 Fig — (A) Violin plots showing quality control metrics for each sample: the total number of Unique Molecular Identifier (UMI) counts (left), the number of observed genes (middle), and percent of mitochondrial genes (right). KI, knock-in. (B) UMAP visualization of all hippocampal cells, grouped by sample. Each sample is represented by a distinct color. (C) Volcano plot showing global differential expression (DE) analysis results for all hippocampal cells based on genotype. (D) Subclustering results of neurons. The left panel presents a UMAP visualization of neuronal subtypes, while the right panel shows a dot plot of cell-specific marker gene expression, with dot size representing the percentage of cells expressing the gene and color intensity indicating average expression levels. (E) Subclustering results of excitatory neurons. The left panel displays a UMAP visualization of excitatory neuronal subtypes, while the right panel features a dot plot highlighting cell-specific marker genes. (F) Subclustering results of pyramidal neurons. The left panel presents a UMAP visualization of neuronal subtypes, while the right panel shows a dot plot of cell-specific marker gene expression. (G) Bar plot showing significant GSEA results for CA1-1 compared to CA1-0. The Y-axis represents Gene Ontology Biological Process (GOBP) terms, while the X-axis represents Normalized Enrichment Scores (NES). Color intensity reflects the significance level, represented by −Log10 (adjusted P value). (TIF) [file pbio.3003192.s015.tif]

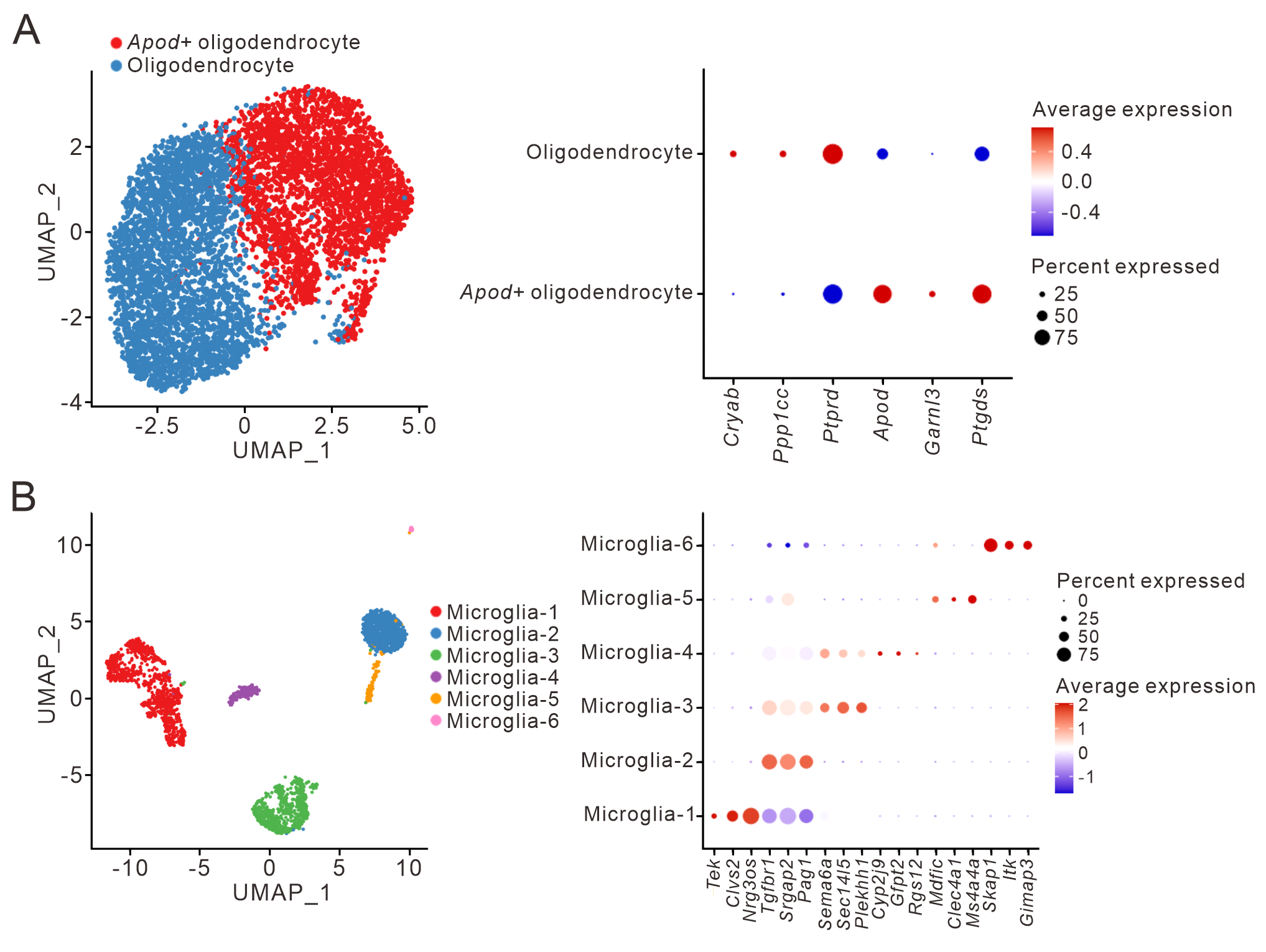

Supplement: S16 Fig — (A) Subclustering results of oligodendrocytes. The left panel presents a UMAP visualization of oligodendrocyte subtypes, while the right panel features a dot plot highlighting the top DEGs for each cluster. (B) Subclustering results of microglia. The left panel presents a UMAP visualization of microglia subtypes, while the right panel features a dot plot highlighting the top DEGs for each cluster. (TIF) [file pbio.3003192.s016.tif]

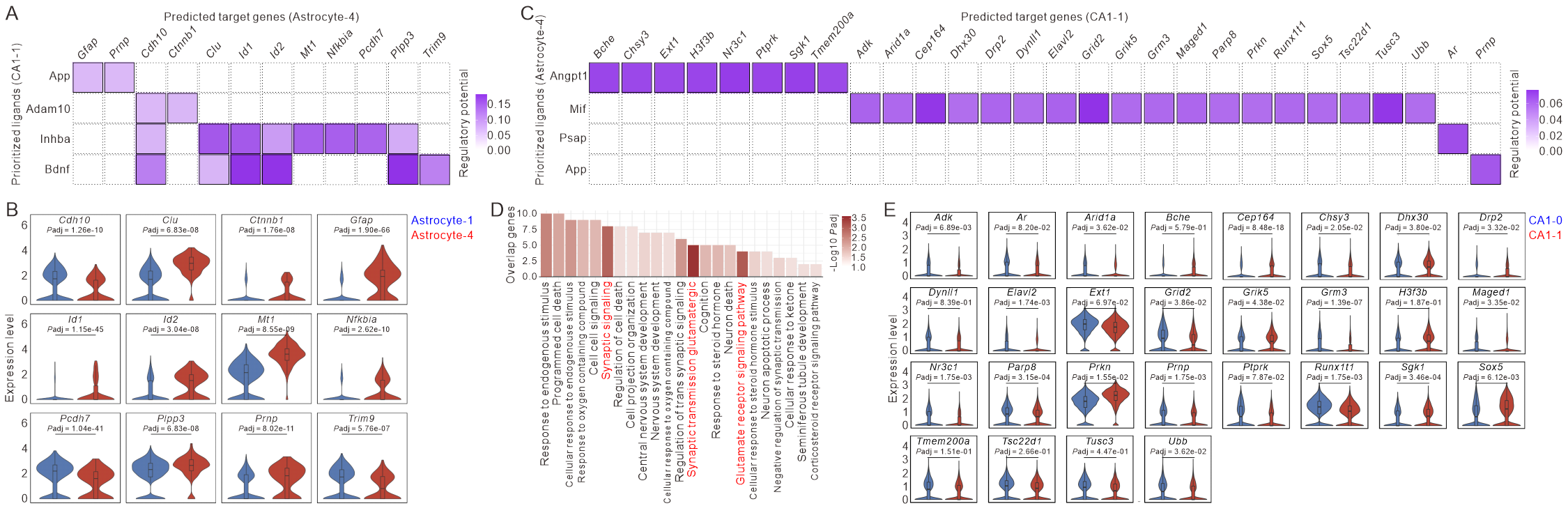

Supplement: S17 Fig — (A) Regulatory potential of ligands (Adam10, App, Bdnf, and Inhba) from CA1-1 on 12 predicted target genes expressed in Astrocyte-4. (B) Expression level comparisons of the 12 predicted target genes between Astrocyte-1 and Astrocyte-4. (C) Regulatory potential of Angpt1, App, Mif, and Psap from Astrocyte-4 on their 28 target genes expressed in CA1-1. (D) Over-Representation Analysis (ORA) result for the 28 target genes. (E) Expression level comparisons of the 28 target genes between CA1-0 and CA1-1. (TIF) [file pbio.3003192.s017.tif]

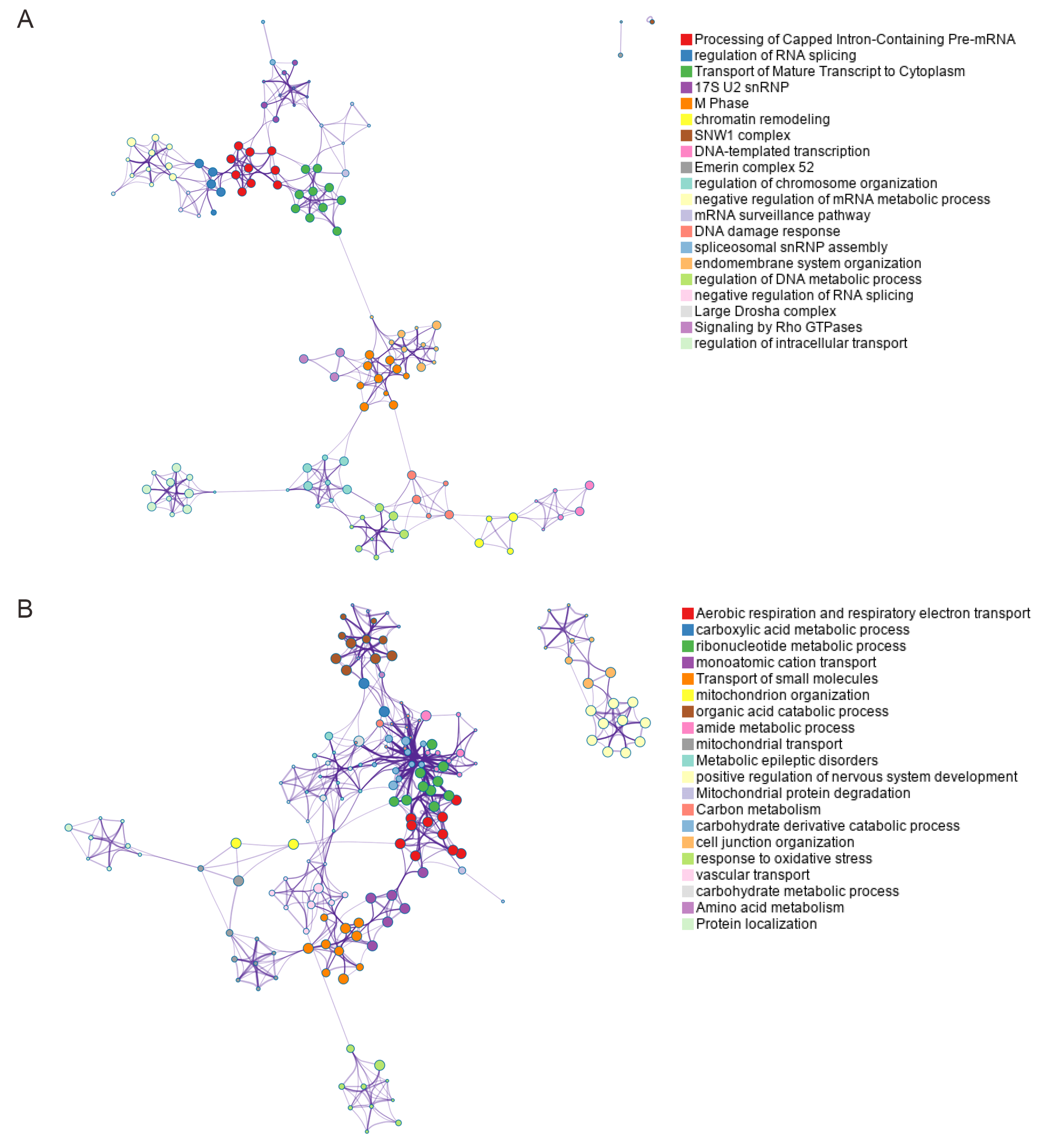

Supplement: S18 Fig — (A) Metascape (https://metascape.org/gp/index.html#/main/step1) network of enrichment terms for group B proteins, with nodes sharing the same cluster ID typically positioned close to one another. (B) Metascape network of enrichment terms for group D proteins. (TIF) [file pbio.3003192.s018.tif]

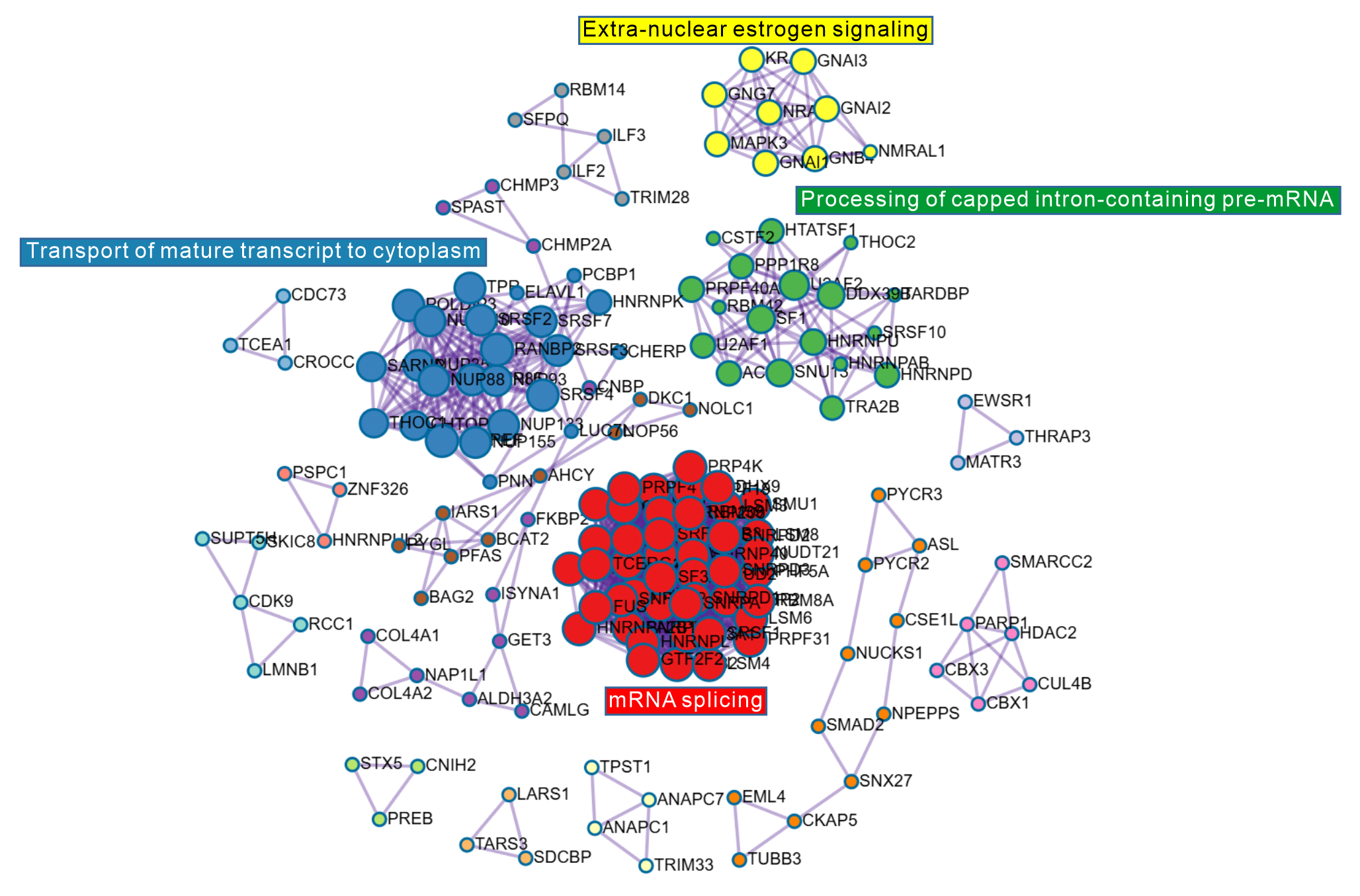

Supplement: S19 Fig — (TIF) [file pbio.3003192.s019.tif]

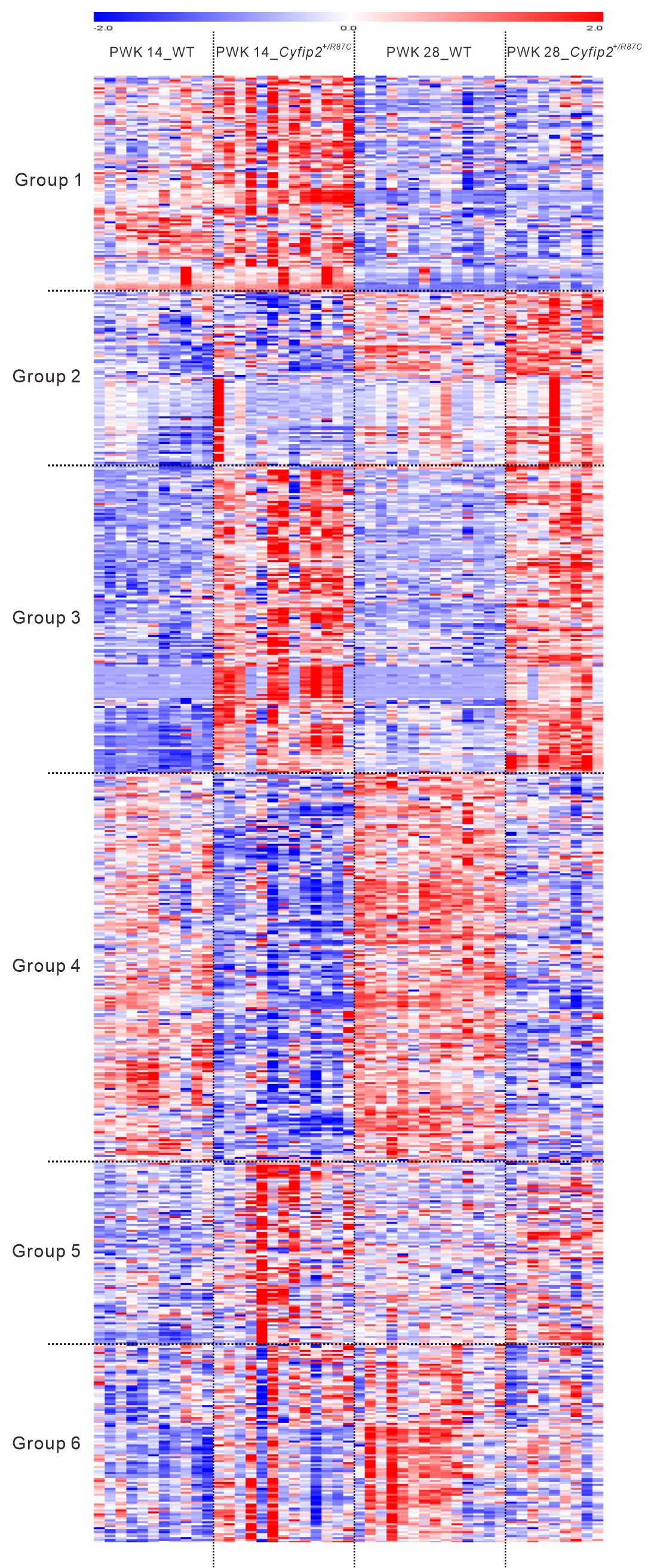

Supplement: S20 Fig — Z-scores are significantly different among different groups (n = 9–14 mice per genotype, Kruskal–Wallis test). (TIF) [file pbio.3003192.s020.tif]
