# Supplementary material for: Seizure evolution in a mouse model of West syndrome involves complex and time-dependent synapse remodeling, gliosis and alterations in lipid metabolism
Source: PLoS Biol. 2025 Oct 9;23(10):e3003192. doi: 10.1371/journal.pbio.3003192 (PMC12520403; doi:10.1371/journal.pbio.3003192)

1wk_CTX

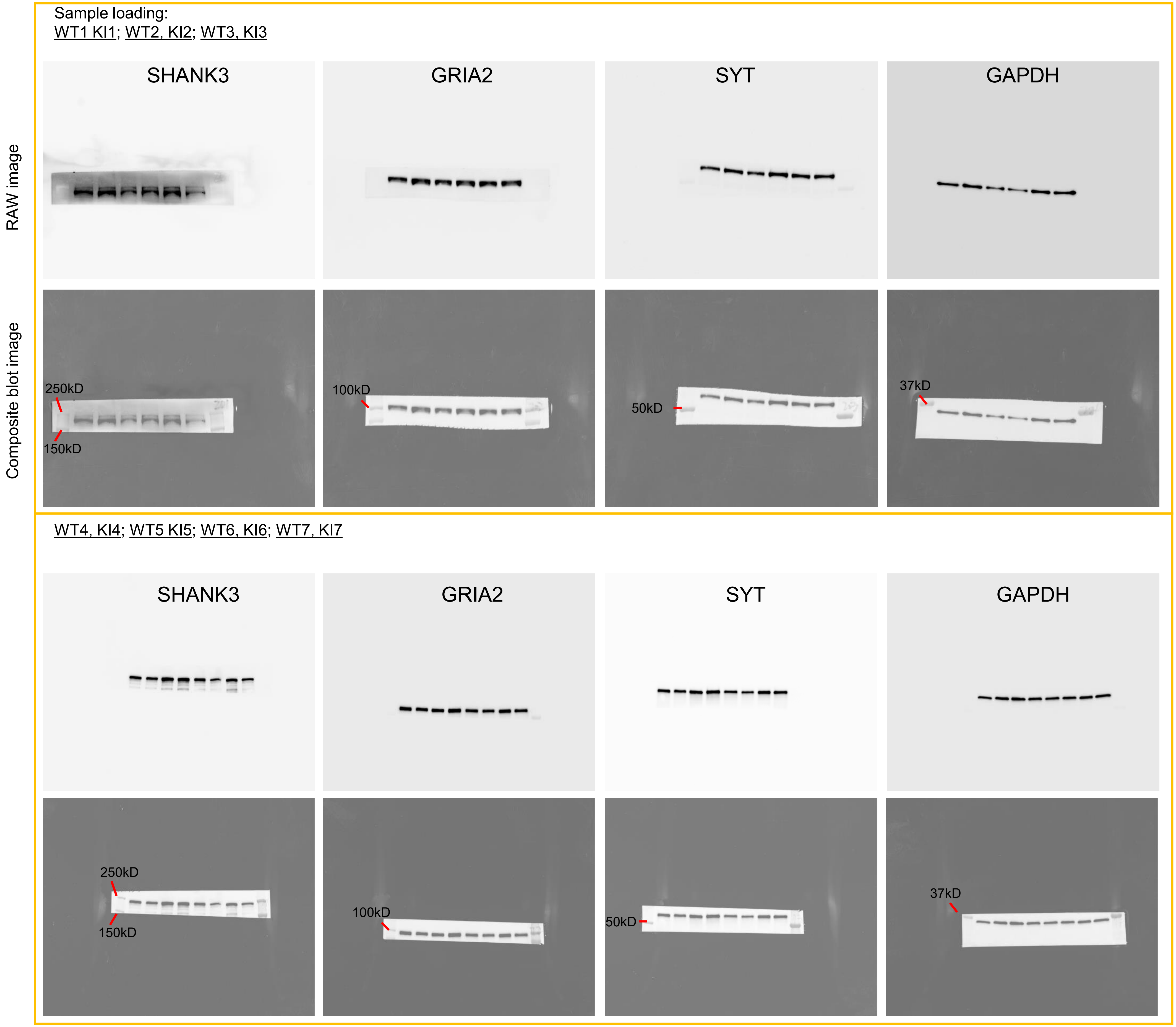
Sample loading:
WT1 KI1; WT2, KI2; WT3, KI3
SHANK3
GRIA2
SYT
GAPDH
RAW image
Composite blot image
250kD
150kD
100kD
50kD
37kD
WT4, KI4; WT5 KI5; WT6, KI6; WT7, KI7
SHANK3
GRIA2
SYT
GAPDH
250kD
150kD
100kD
50kD
37kD

1wk_CTX

Sample loading:
WT1 KI1; WT2, KI2; WT3, KI3

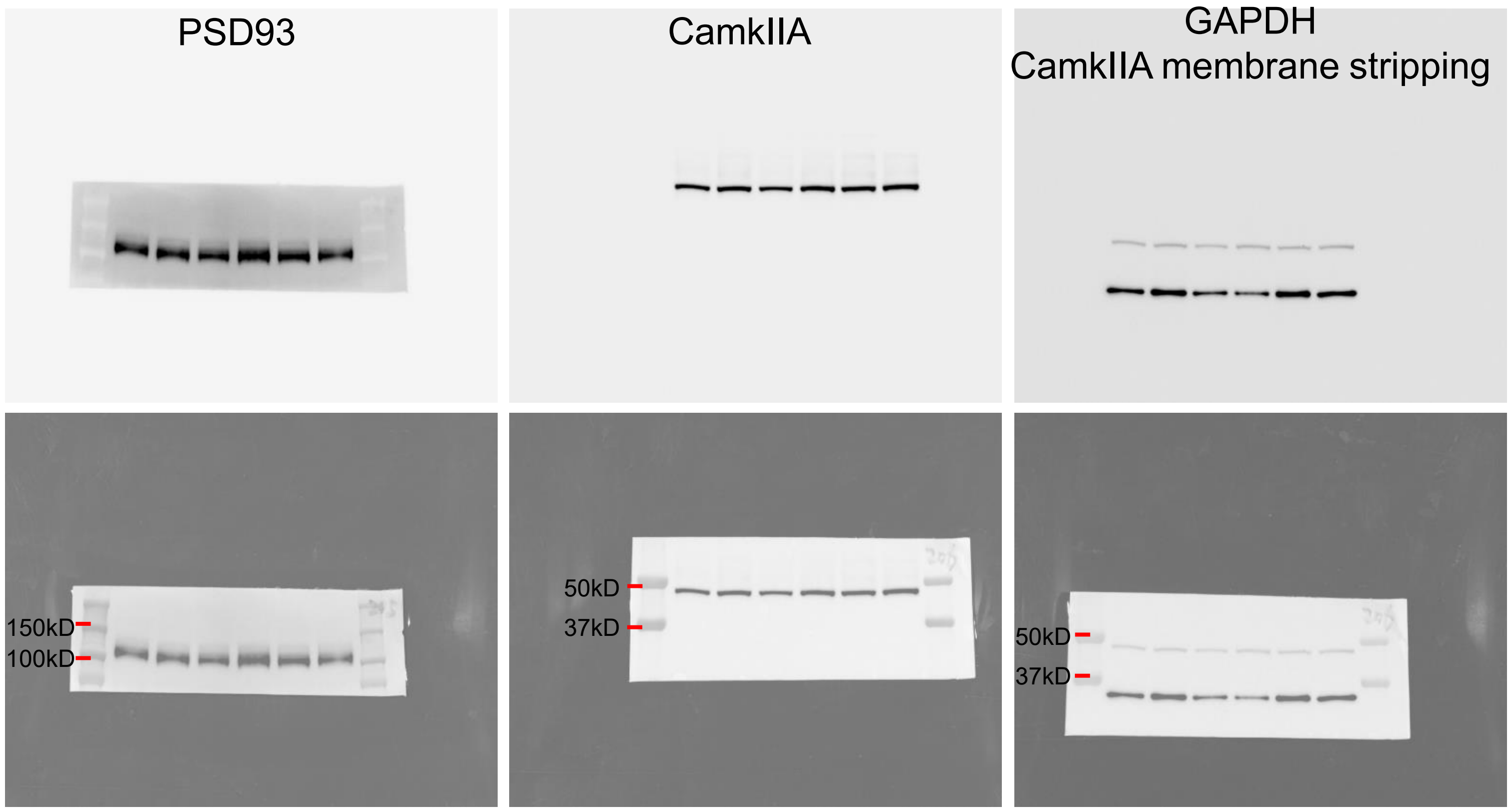

WT4, KI4; WT5 KI5; WT6, KI6; WT7, KI7

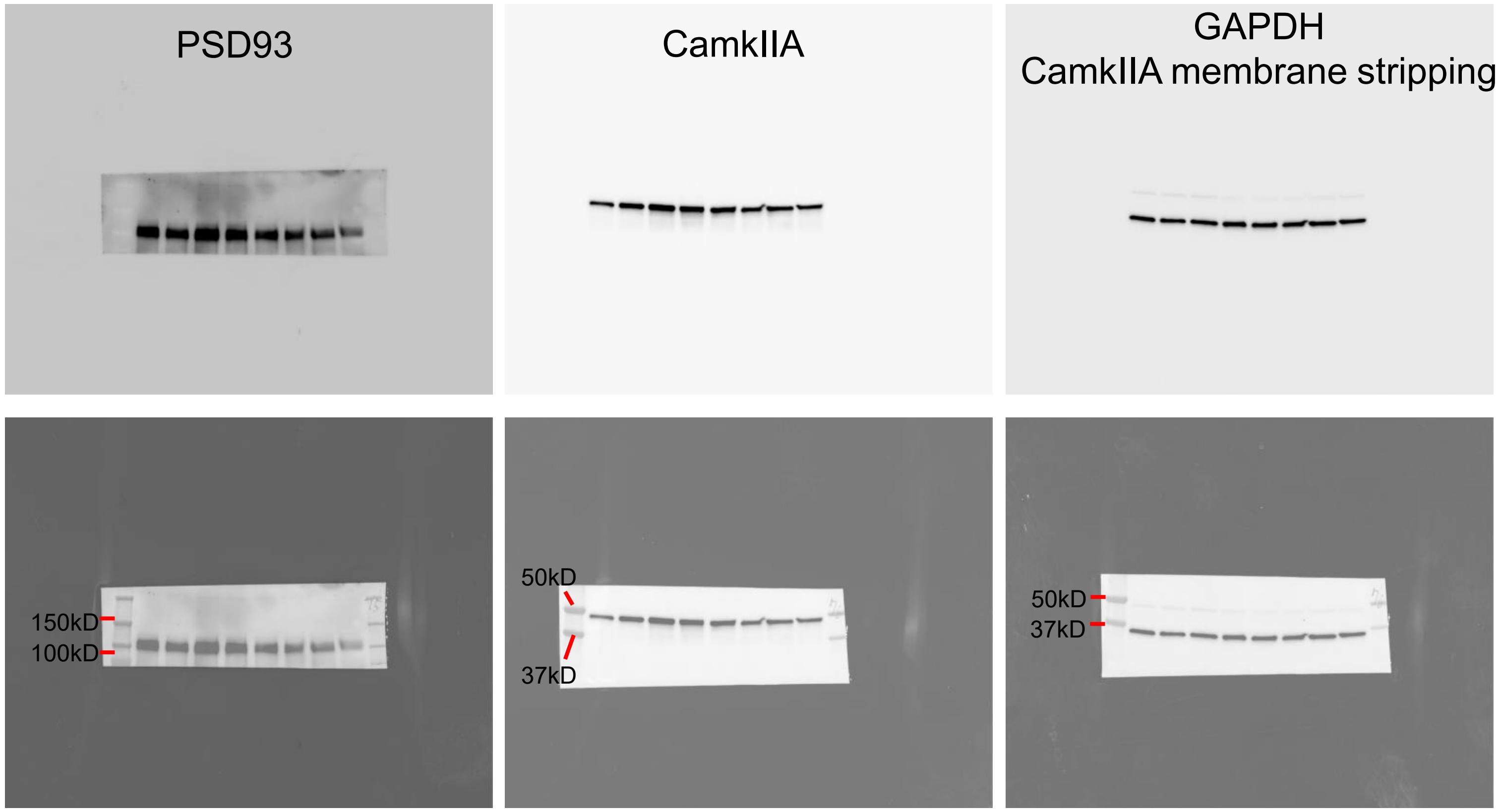

1wk_CTX

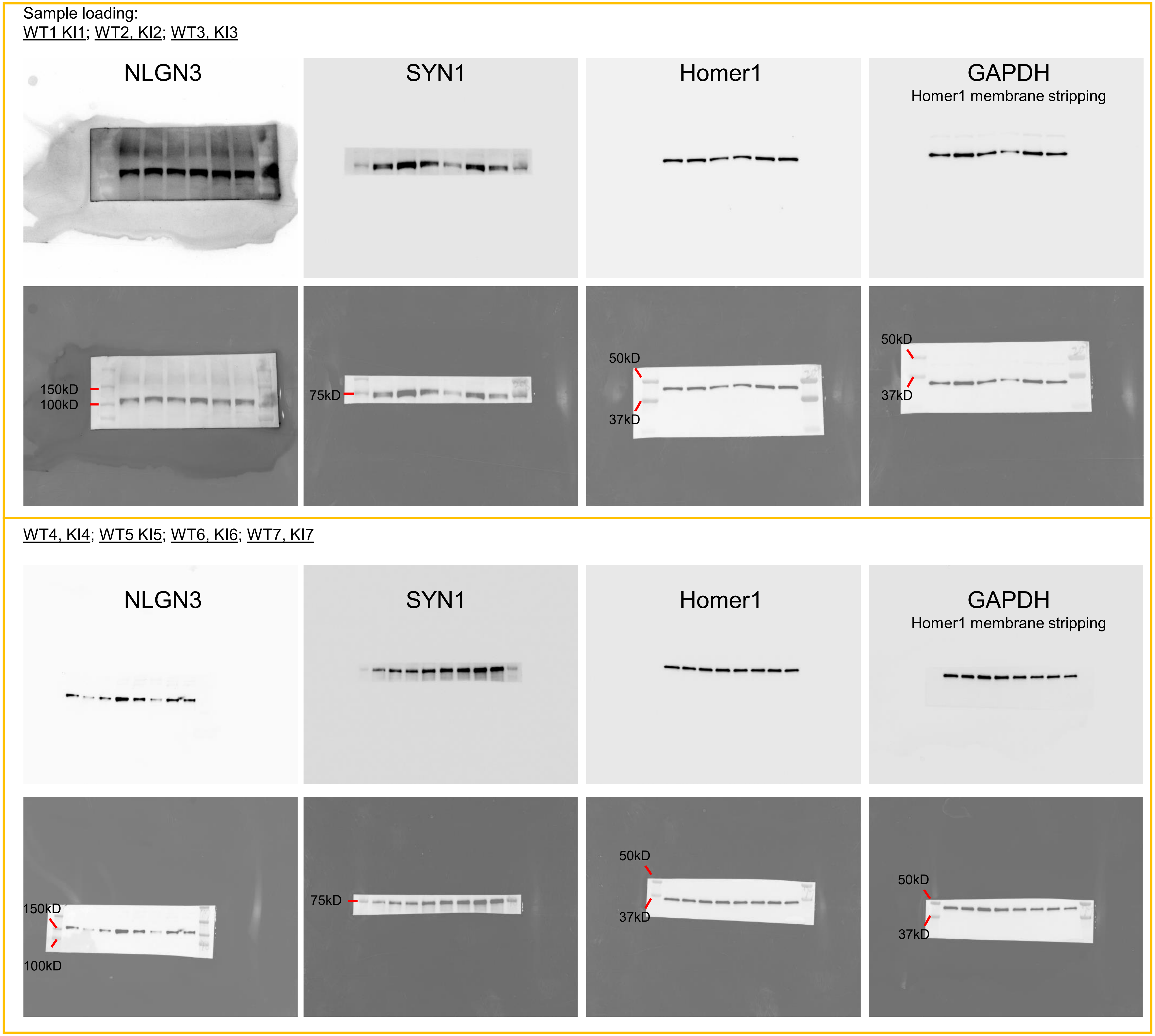

Sample loading:
WT1 KI1; WT2, KI2; WT3, KI3
NLGN3
SYN1
Homer1
GAPDH
Homer1 membrane stripping
150kD
100kD
75kD
50kD
37kD
50kD
37kD
WT4, KI4; WT5 KI5; WT6, KI6; WT7, KI7
NLGN3
SYN1
Homer1
GAPDH
Homer1 membrane stripping
150kD
100kD
75kD
50kD
37kD
50kD
37kD

1wk_CTX

Sample loading:
WT1 KI1; WT2, KI2; WT3, KI3

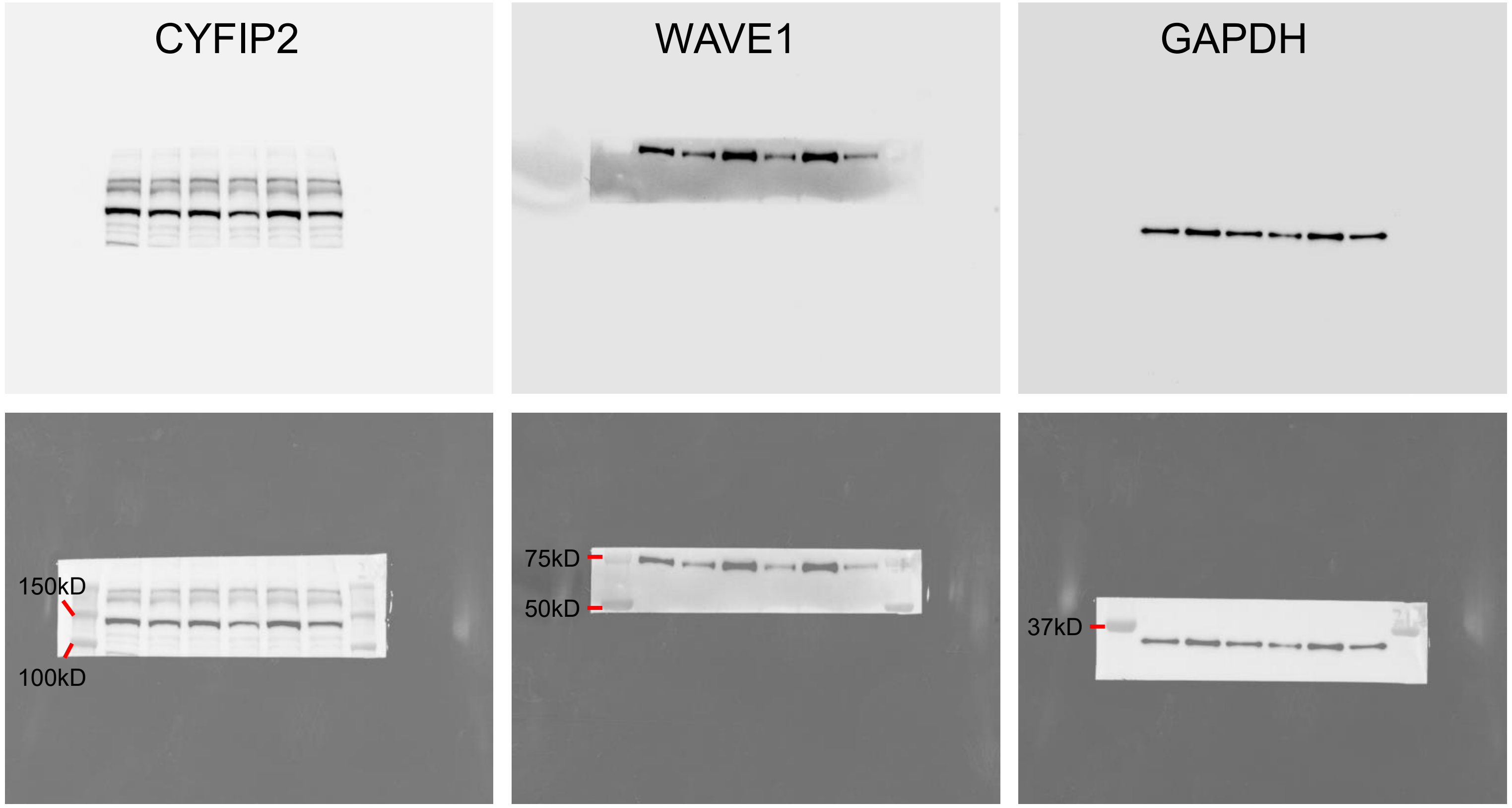

WT4, KI4; WT5 KI5; WT6, KI6; WT7, KI7

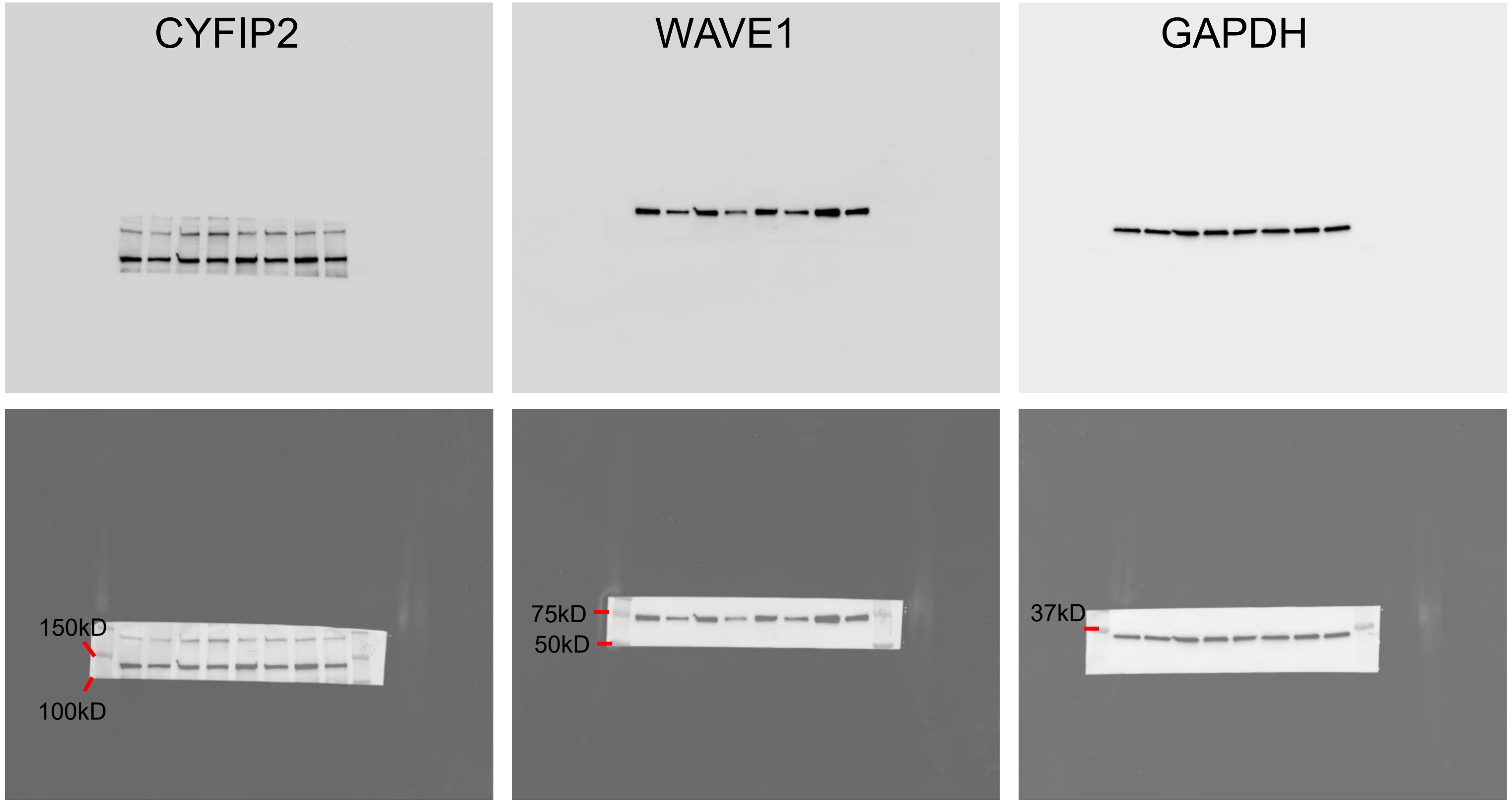

7wk_CTX

Sample loading:
WT1 KI1; WT2, KI2; WT3, KI3; WT4, KI4

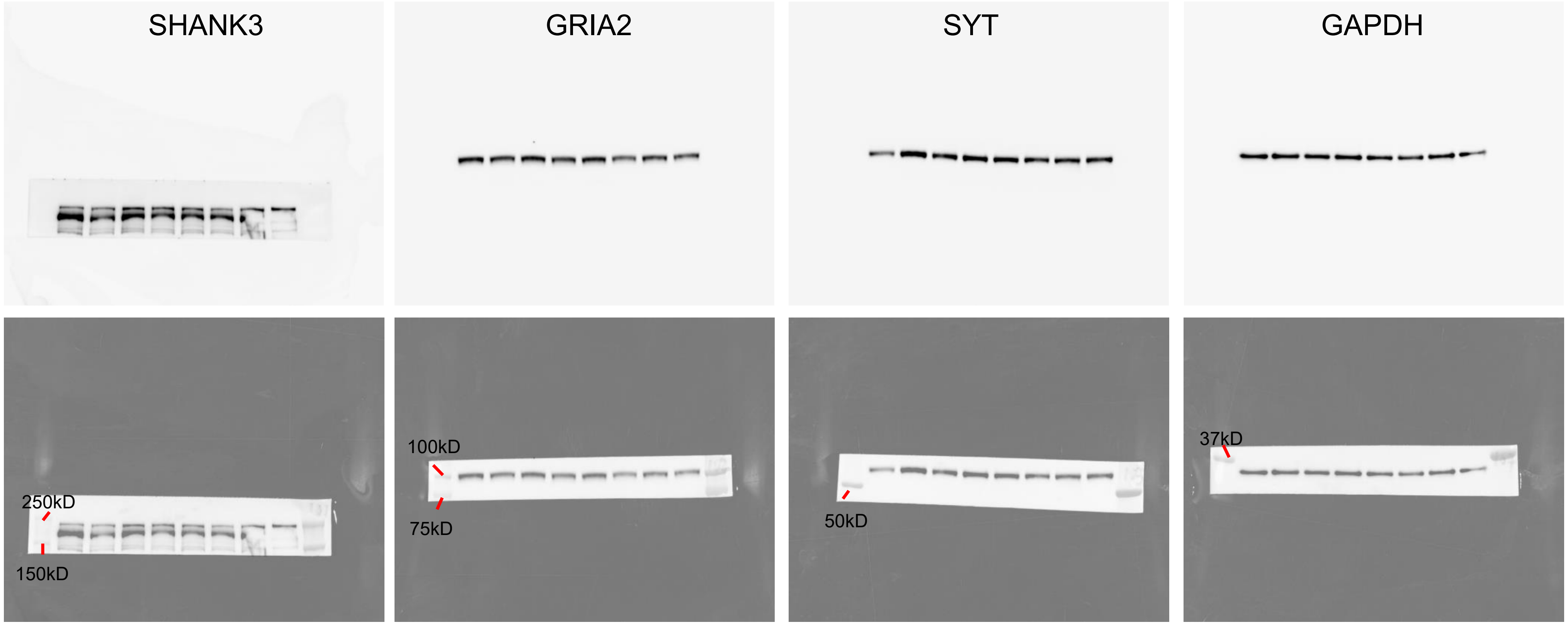

WT5 KI5; WT6, KI6; WT7, KI7; KI8

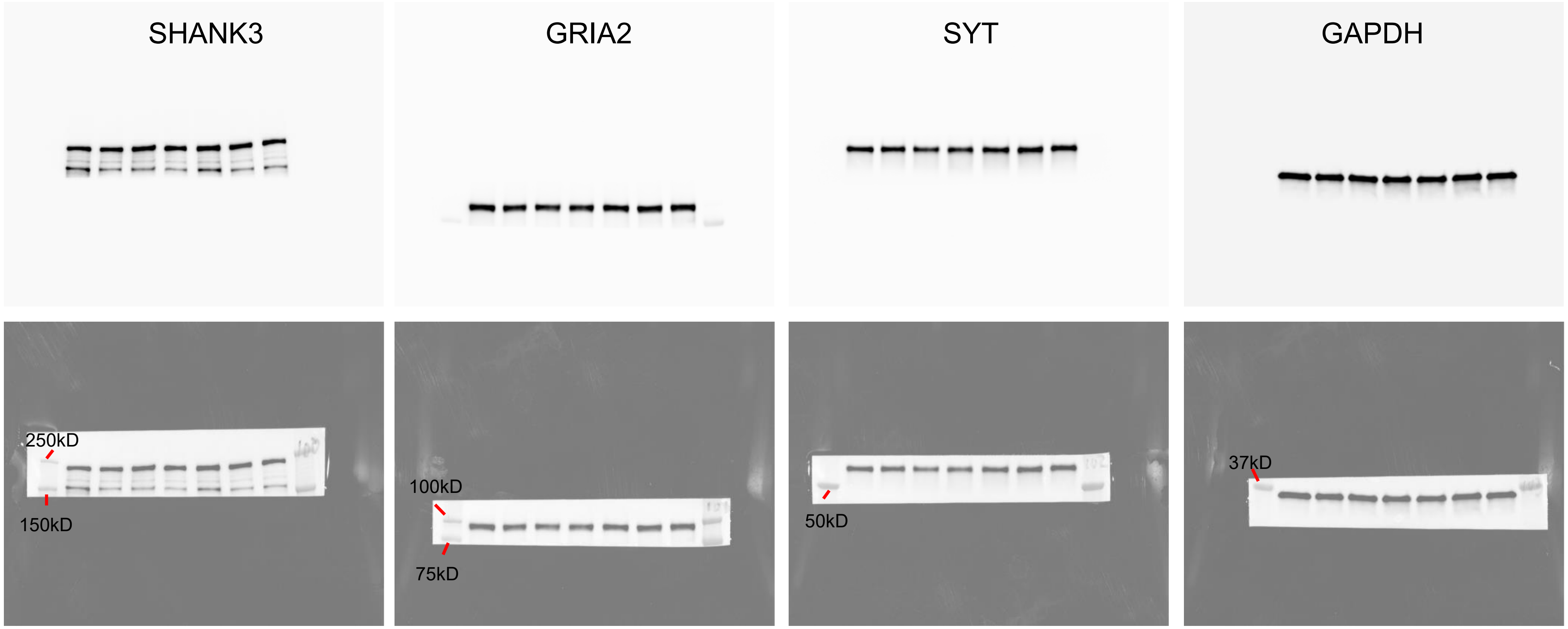

7wk_CTX

Sample loading:

WT1 KI1; WT2, KI2; WT3, KI3; WT4, KI4

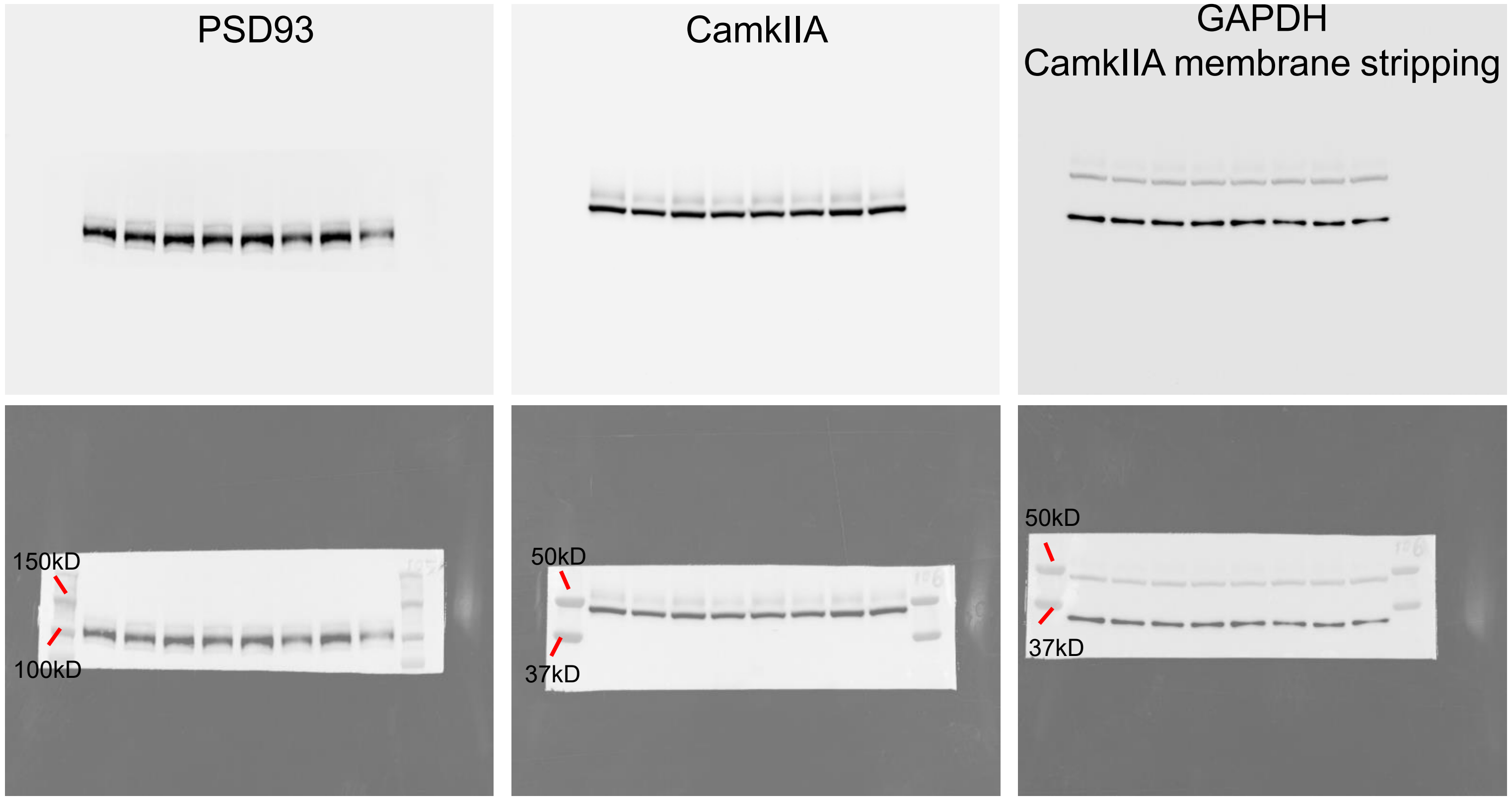

WT5 KI5; WT6, KI6; WT7, KI7; KI8

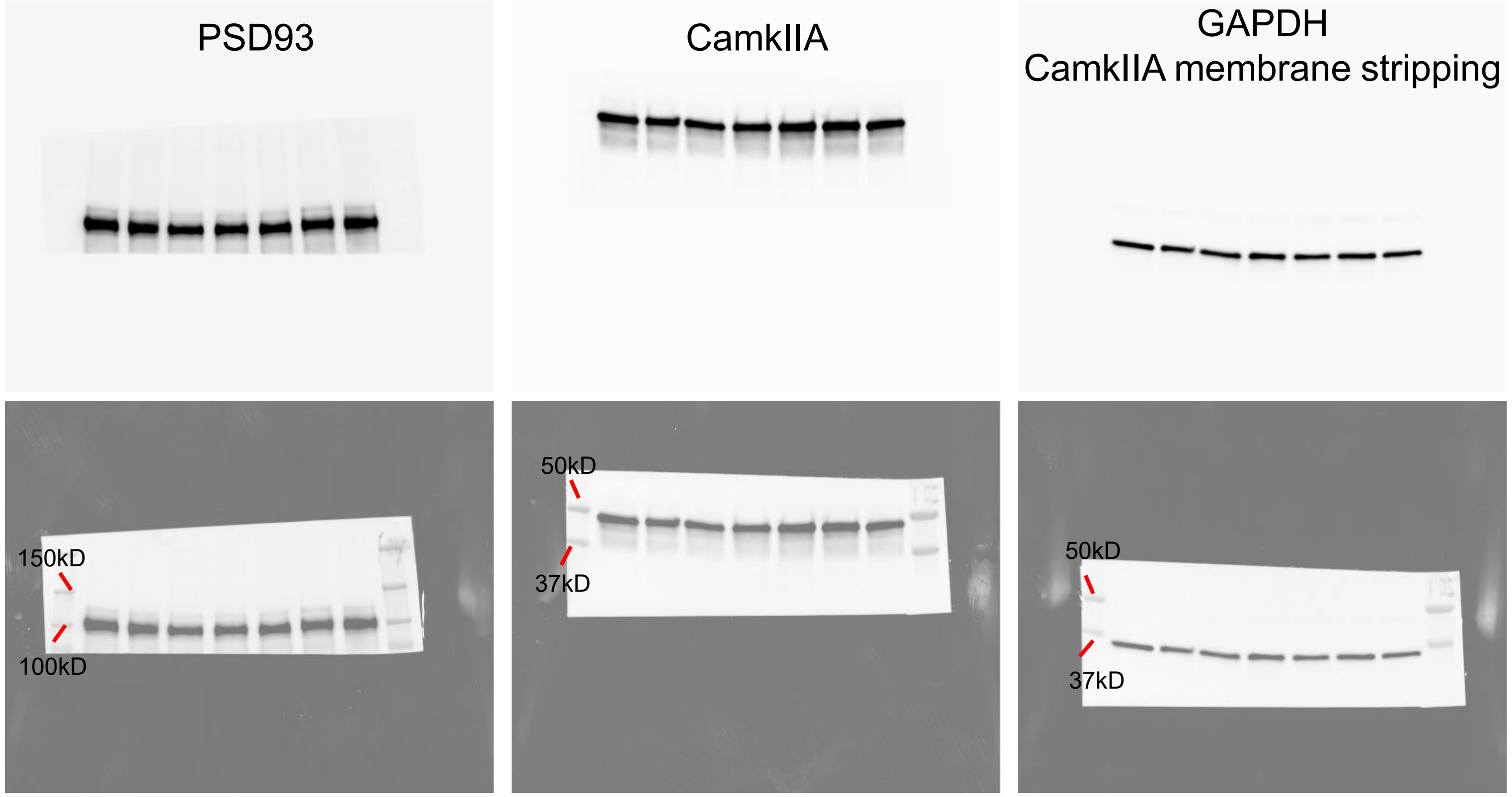

7wk_CTX

Sample loading:
WT1 KI1; WT2, KI2; WT3, KI3; WT4, KI4

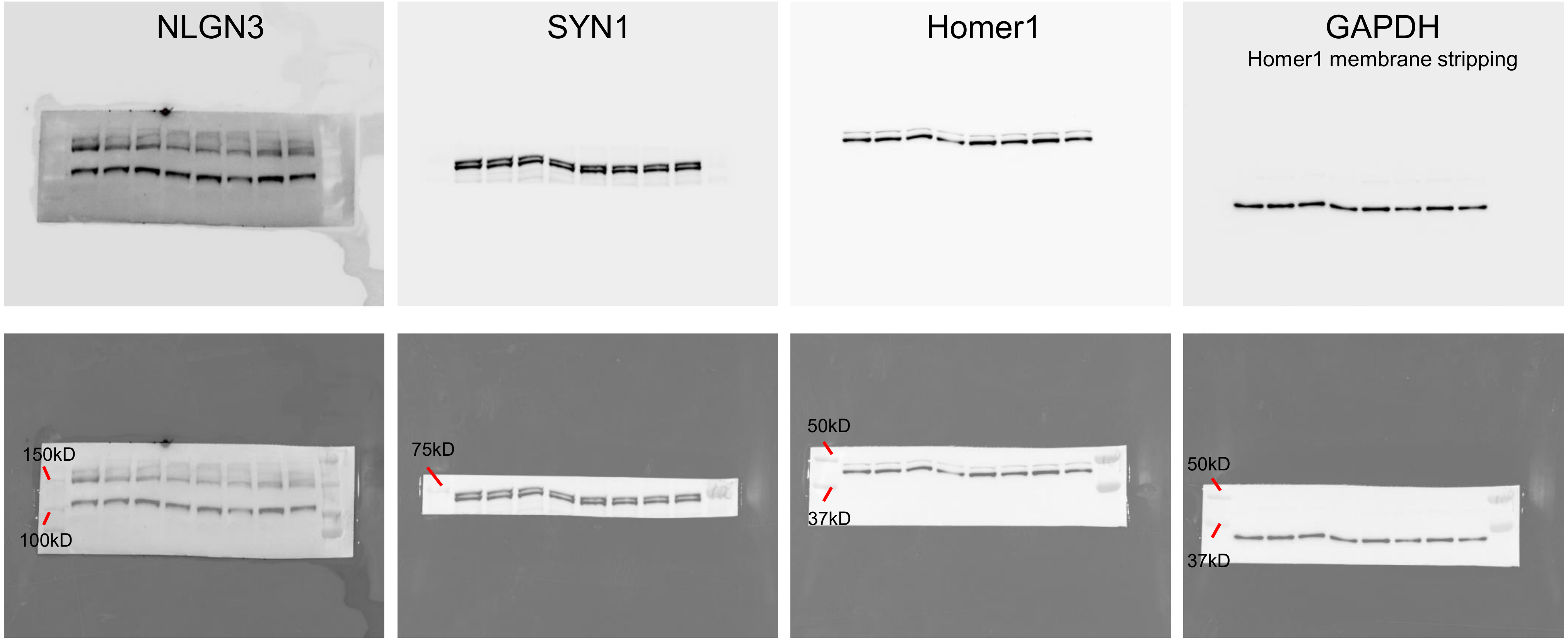

WT5 KI5; WT6, KI6; WT7, KI7; KI8

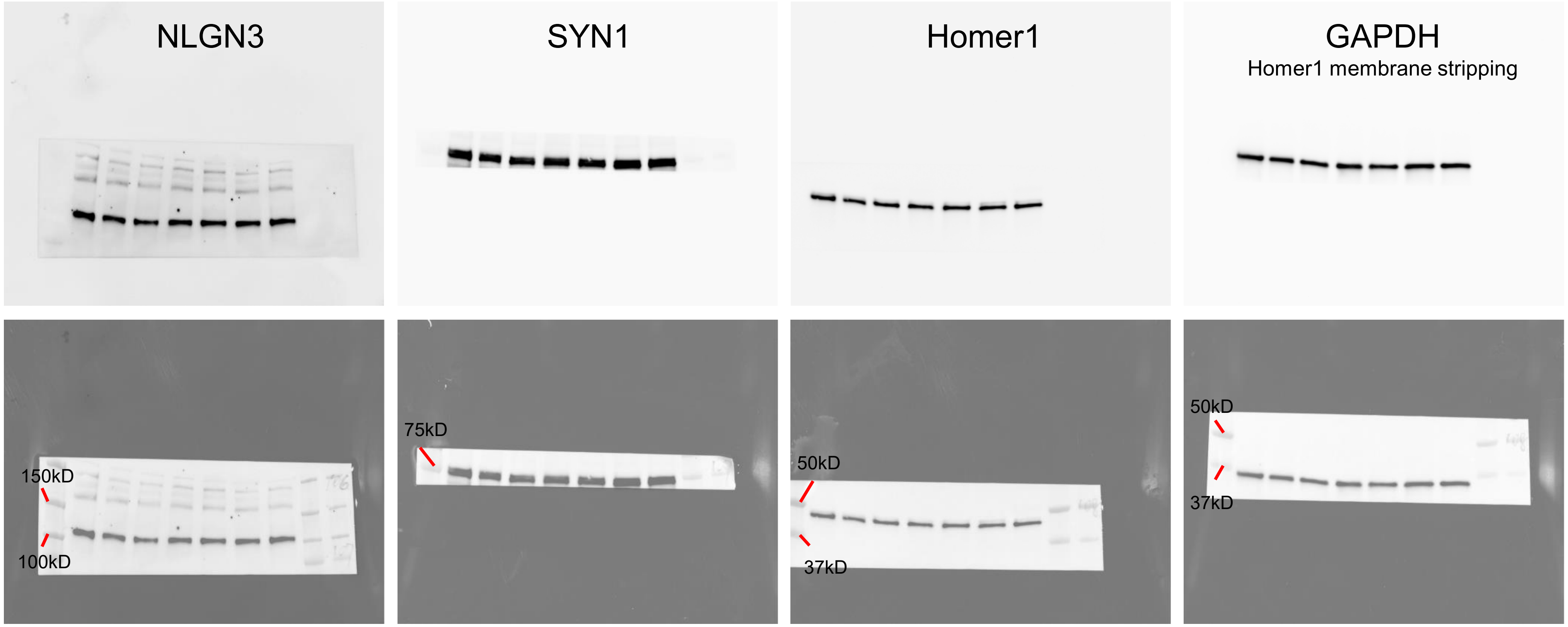

7wk_CTX

Sample loading:
WT1 KI1; WT2, KI2; WT3, KI3; WT4, KI4

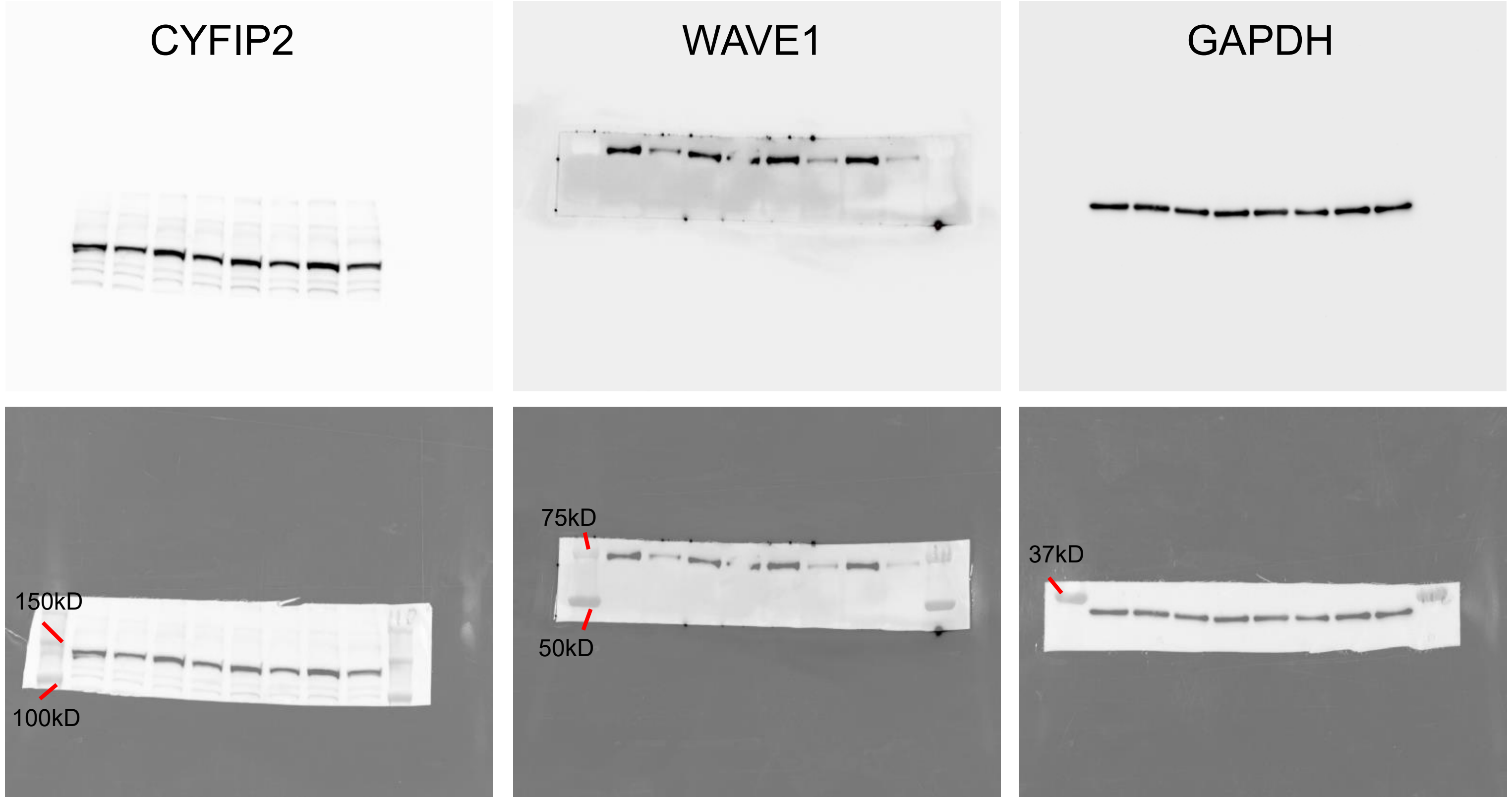

WT5 KI5; WT6, KI6; WT7, KI7; KI8

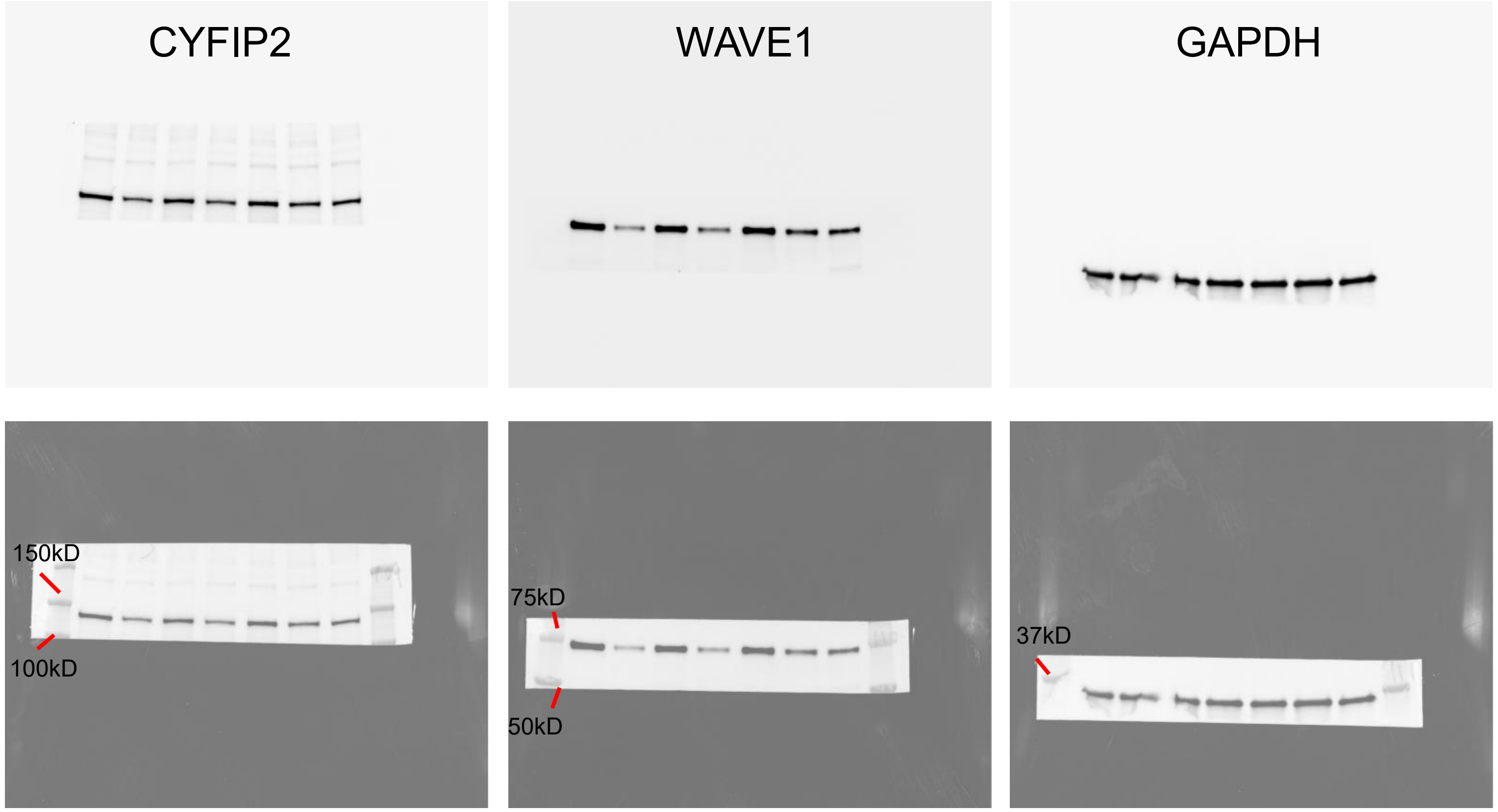

14wk_CTX

Sample loading:
WT1 KI1; WT2, KI2; WT3, KI3; WT4, KI4

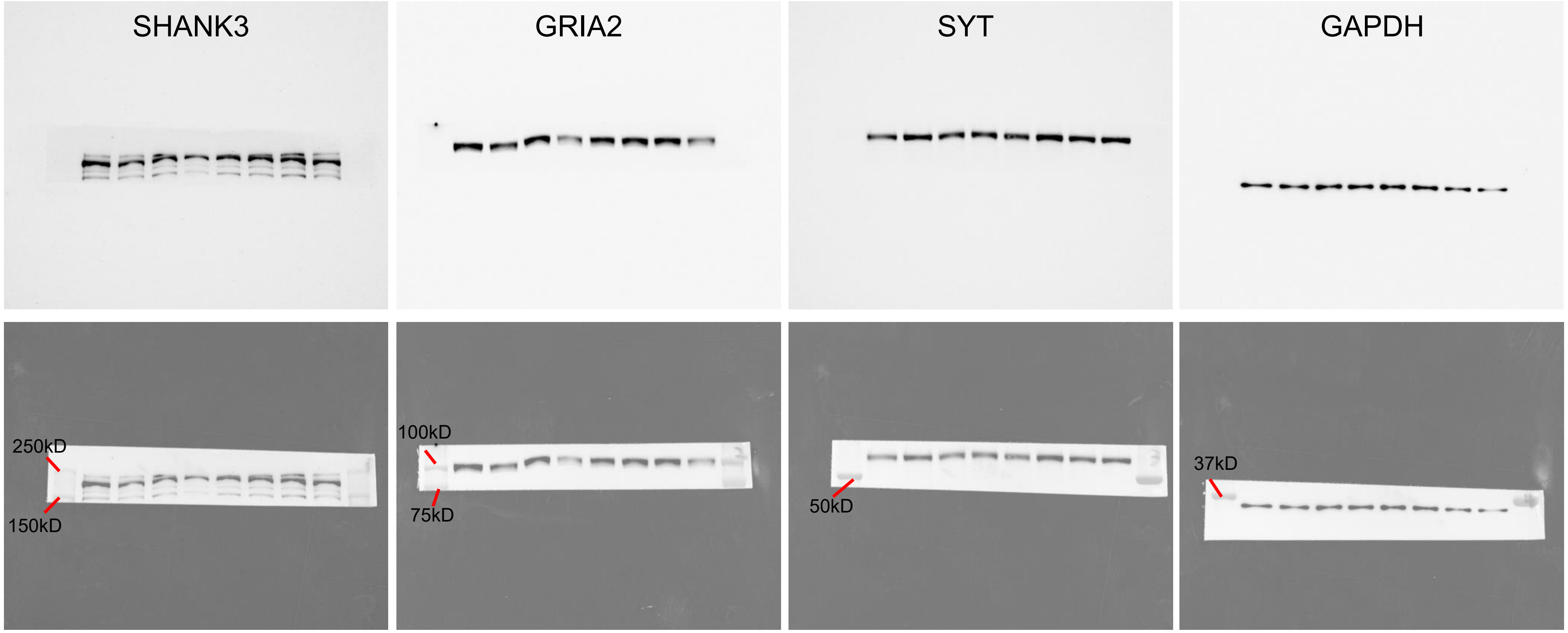

14wk_CTX

Sample loading:
WT3 KI3; X; WT4, KI4; WT5, KI5;

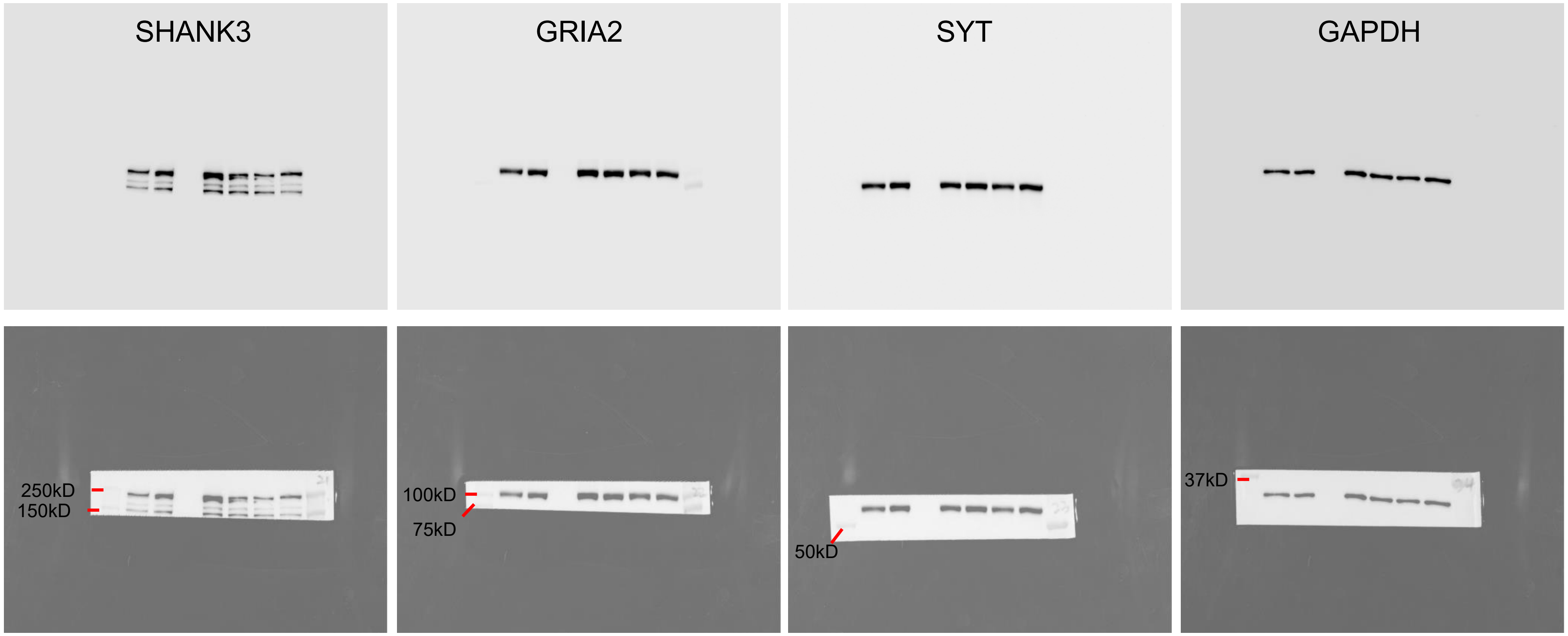

WT6, KI6; X; WT7, KI7; WT8, KI8

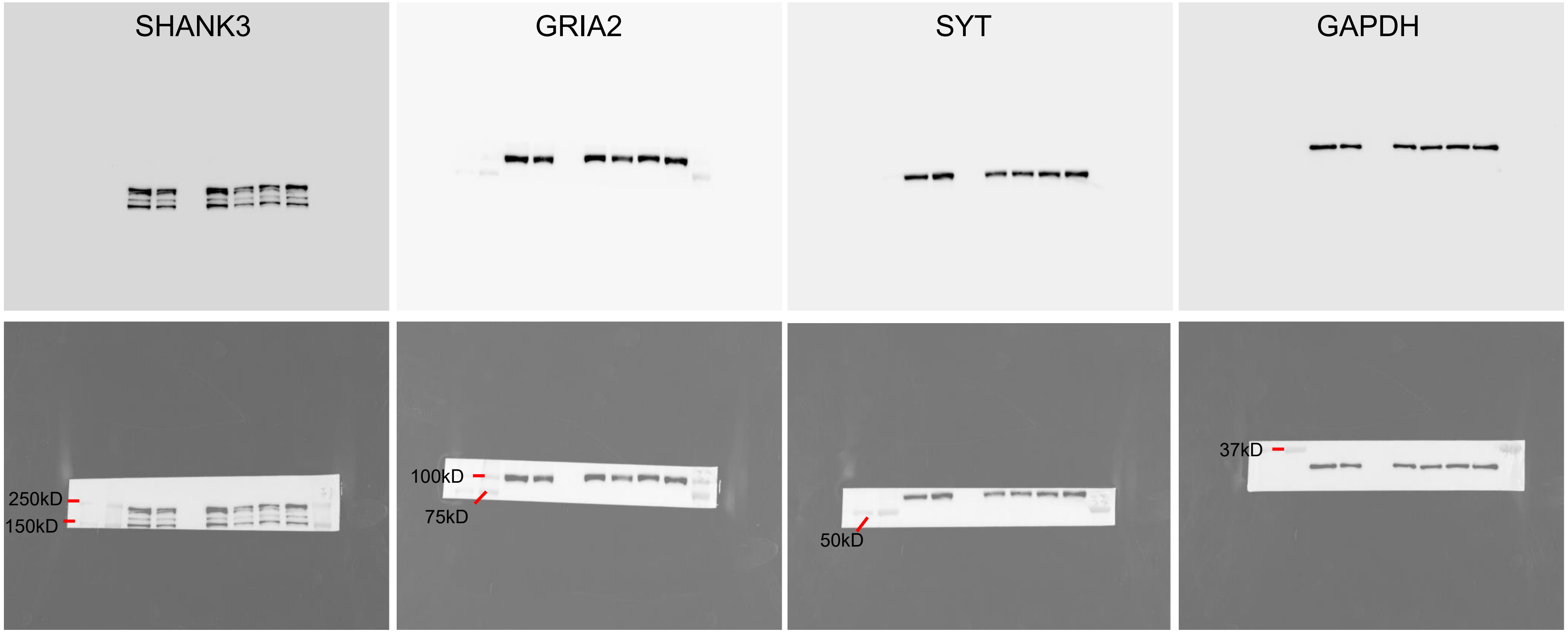

14wk_CTX

Sample loading:
WT1 KI1; WT2, KI2; WT3, KI3; WT4, KI4

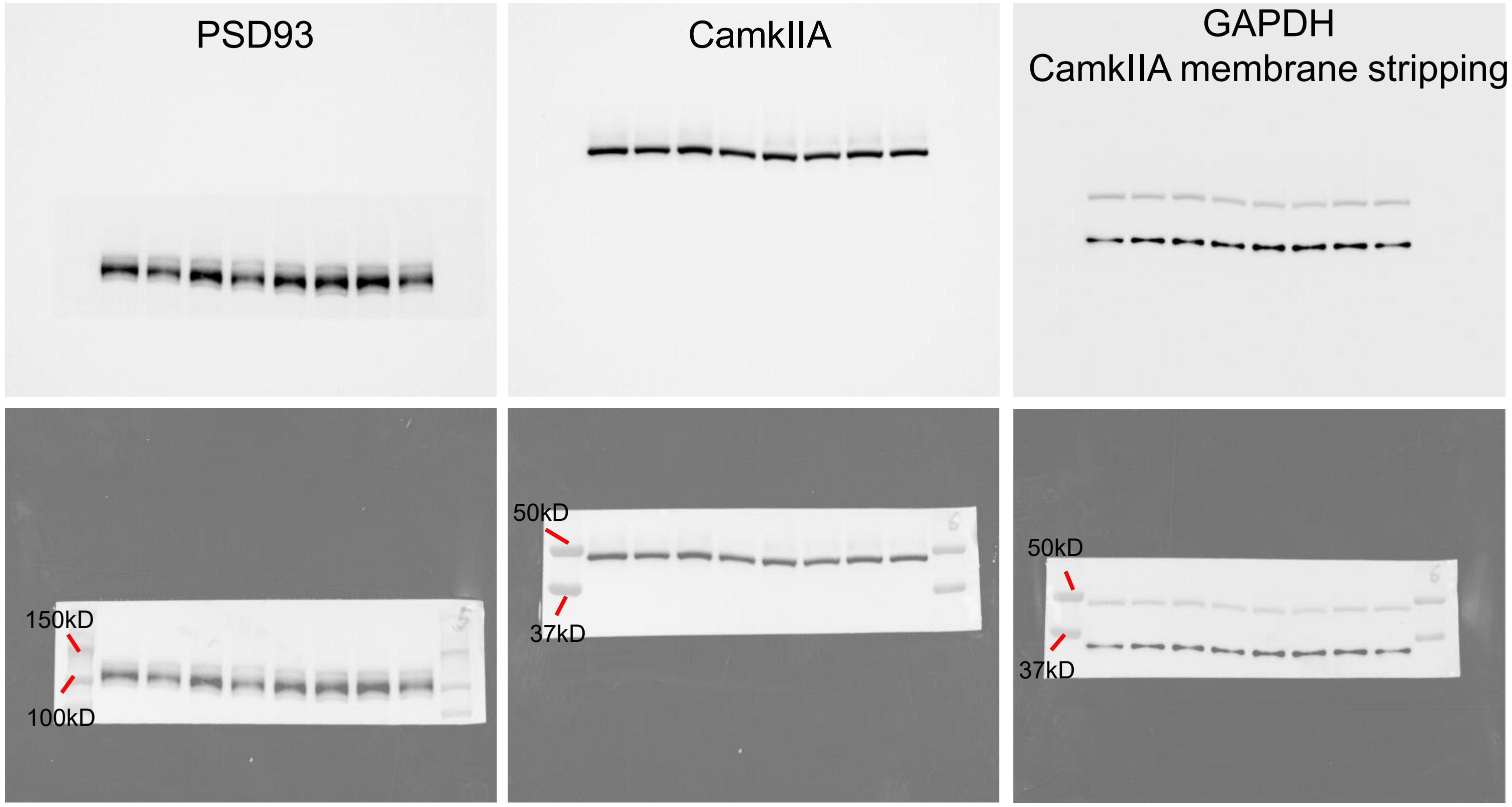

14wk_CTX

Sample loading:

WT3 KI3; X; WT4, KI4; WT5, KI5;

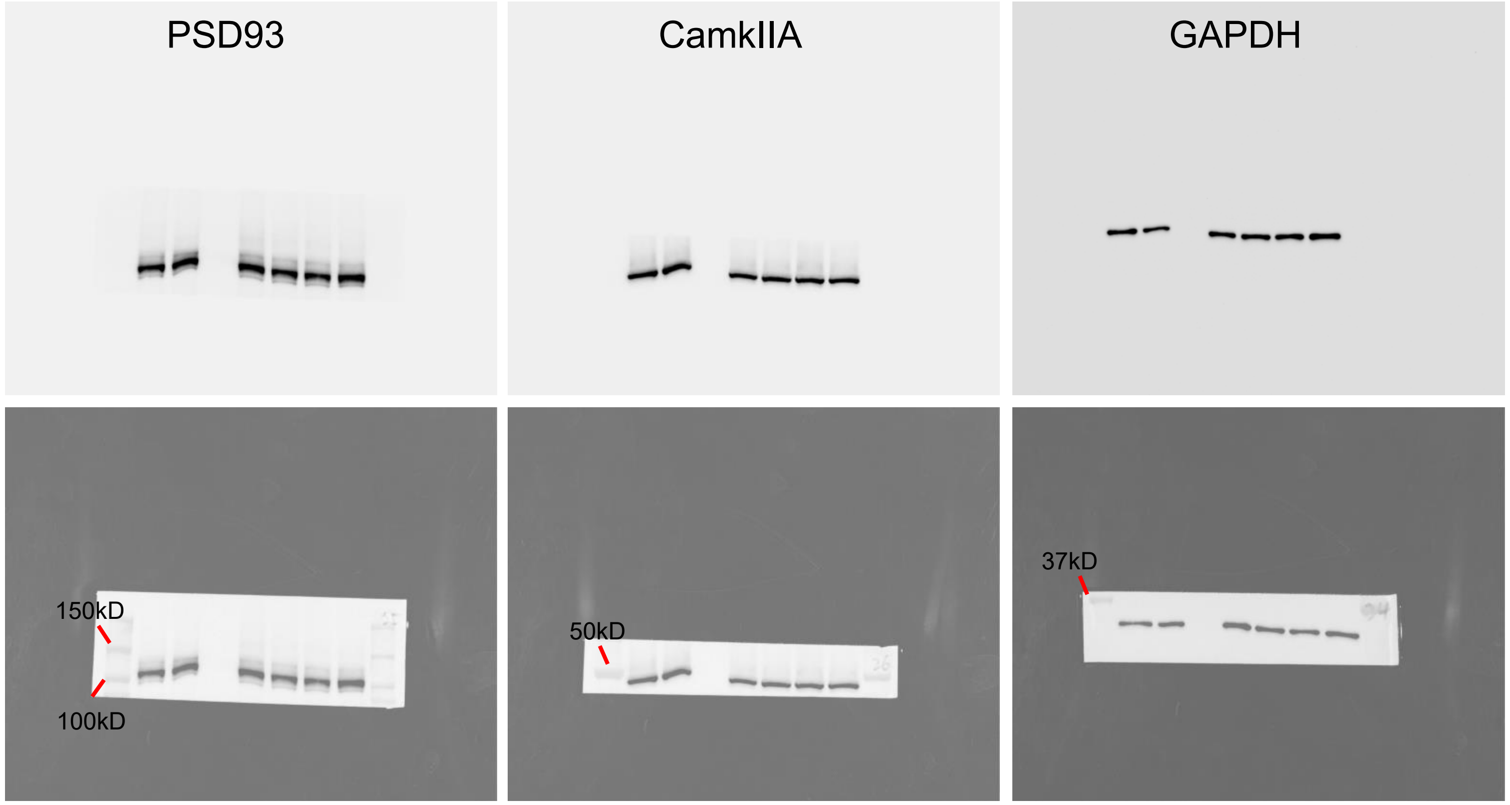

WT6, KI6; X; WT7, KI7; WT8, KI8

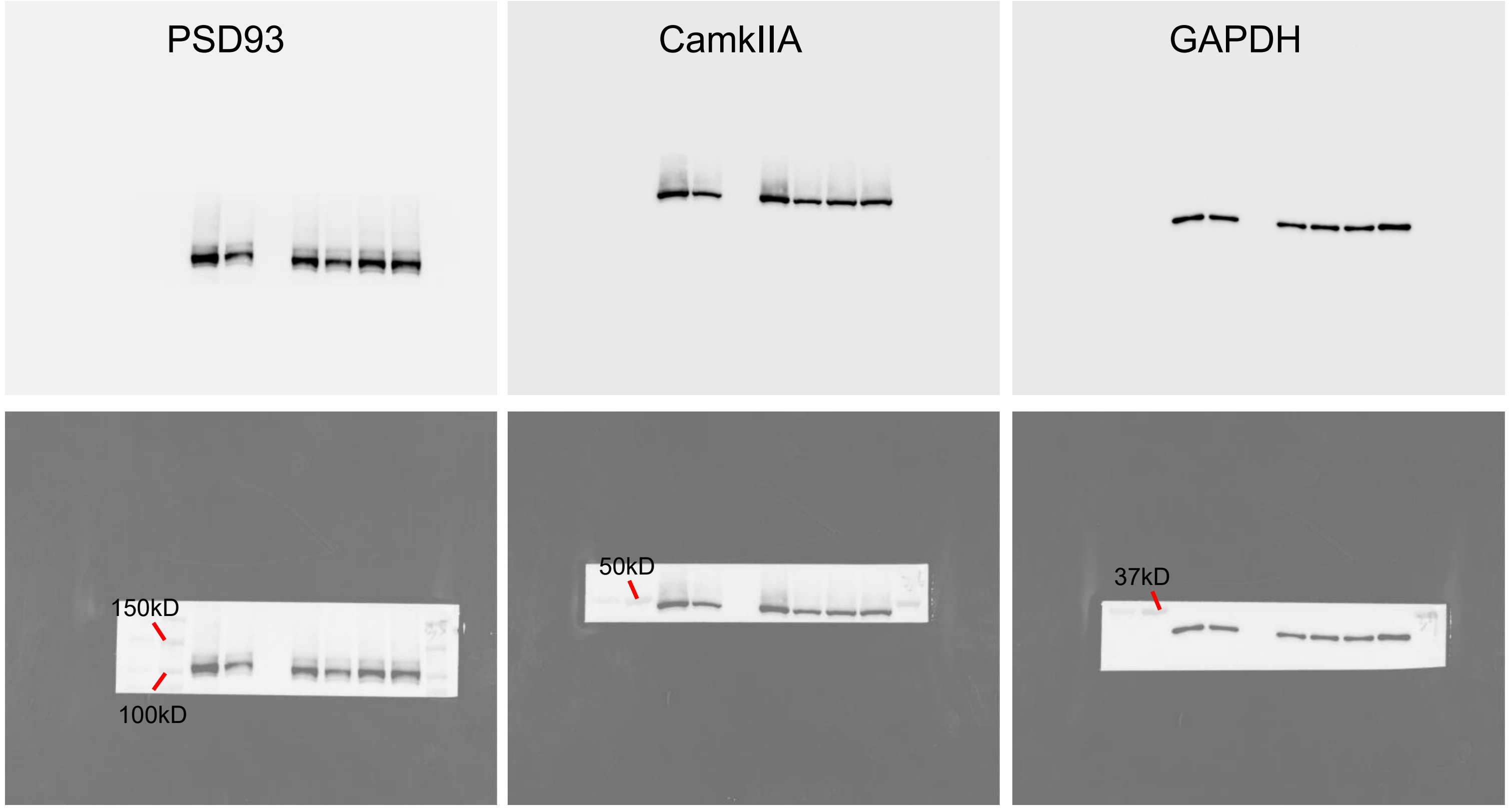

14wk_CTX

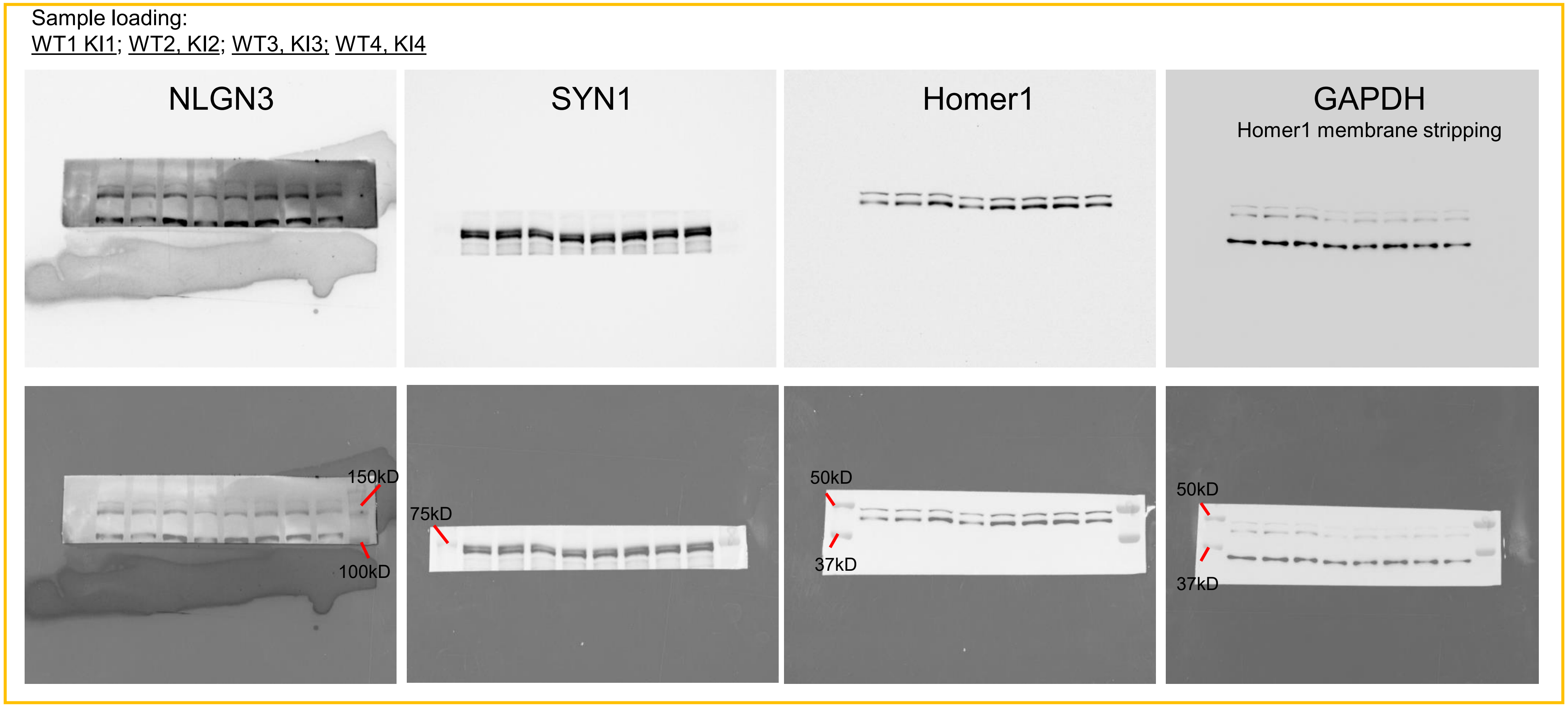
Sample loading:
WT1 KI1; WT2, KI2; WT3, KI3; WT4, KI4
NLGN3
SYN1
Homer1
GAPDH
Homer1 membrane stripping
150kD
100kD
75kD
50kD
37kD
50kD
37kD

14wk_CTX

Sample loading:

WT3 KI3; X; WT4, KI4; WT5, KI5;

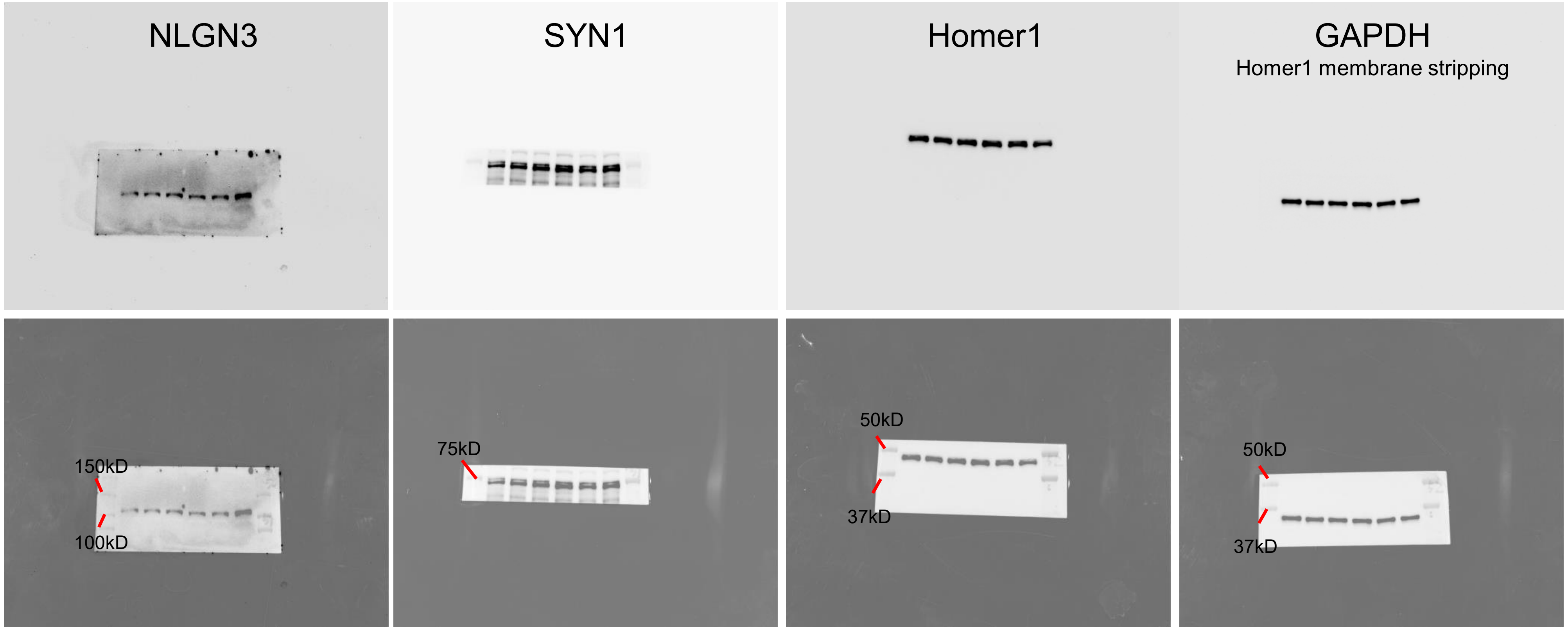

WT6, KI6; X; WT7, KI7; WT8, KI8

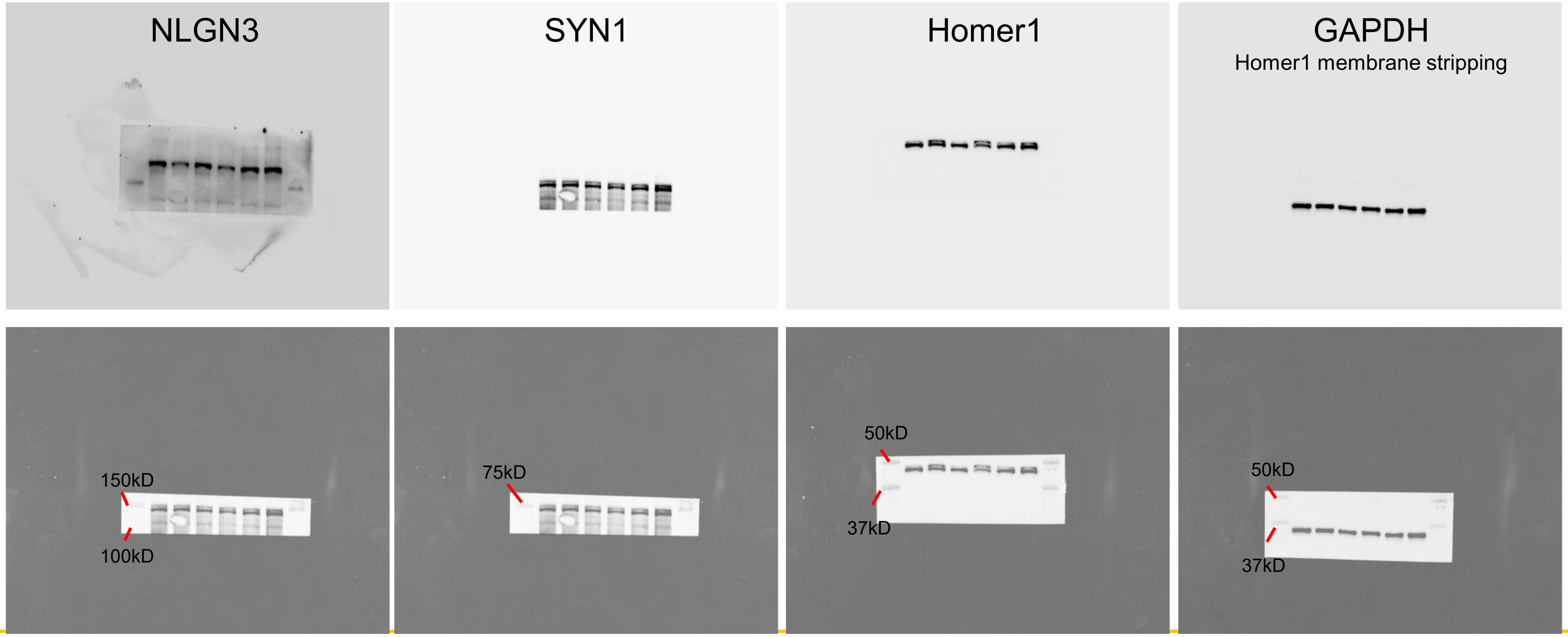

14wk_CTX

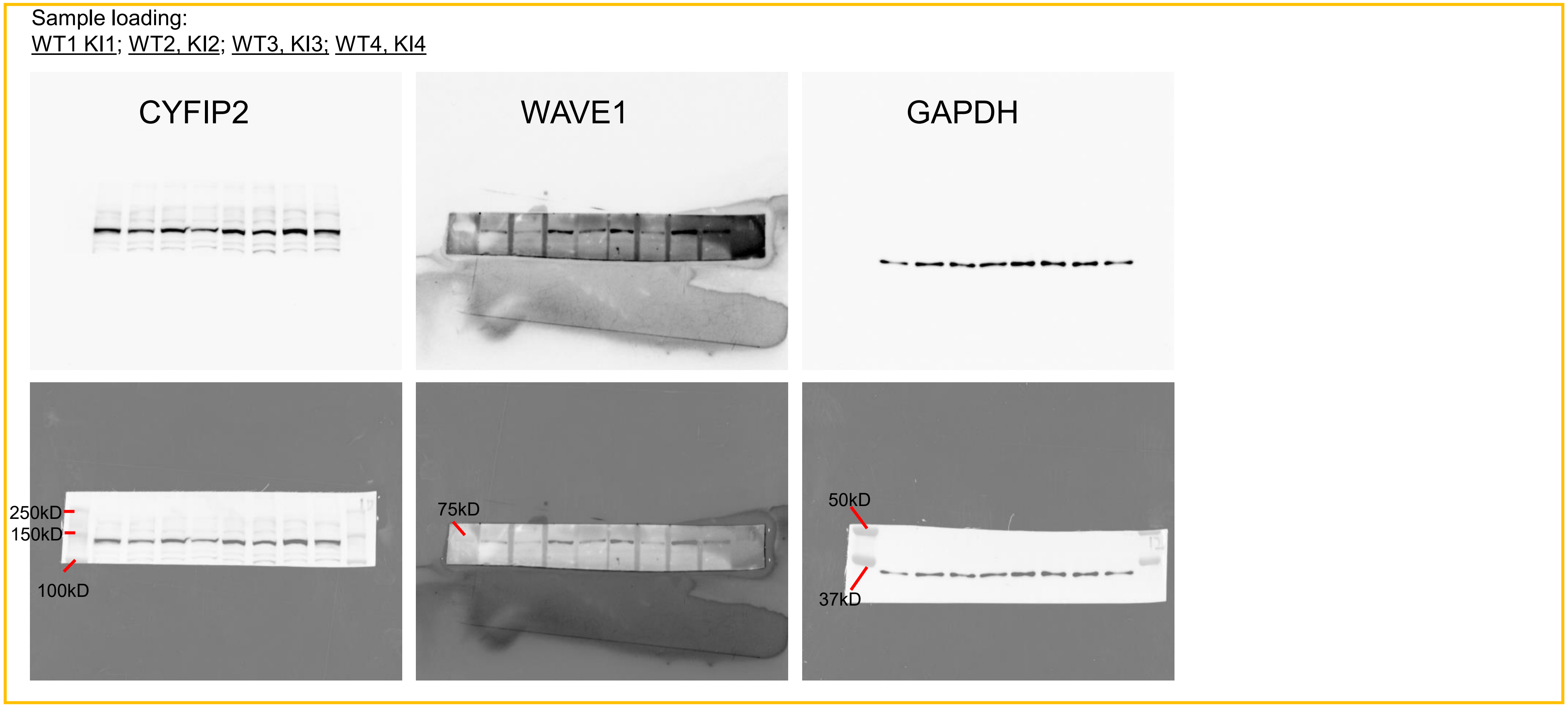
Sample loading:
WT1 KI1; WT2, KI2; WT3, KI3; WT4, KI4
CYFIP2
WAVE1
GAPDH
250kD
150kD
100kD
75kD
50kD
37kD

14wk_CTX

Sample loading:
WT3 KI3; X; WT4, KI4; WT5, KI5;

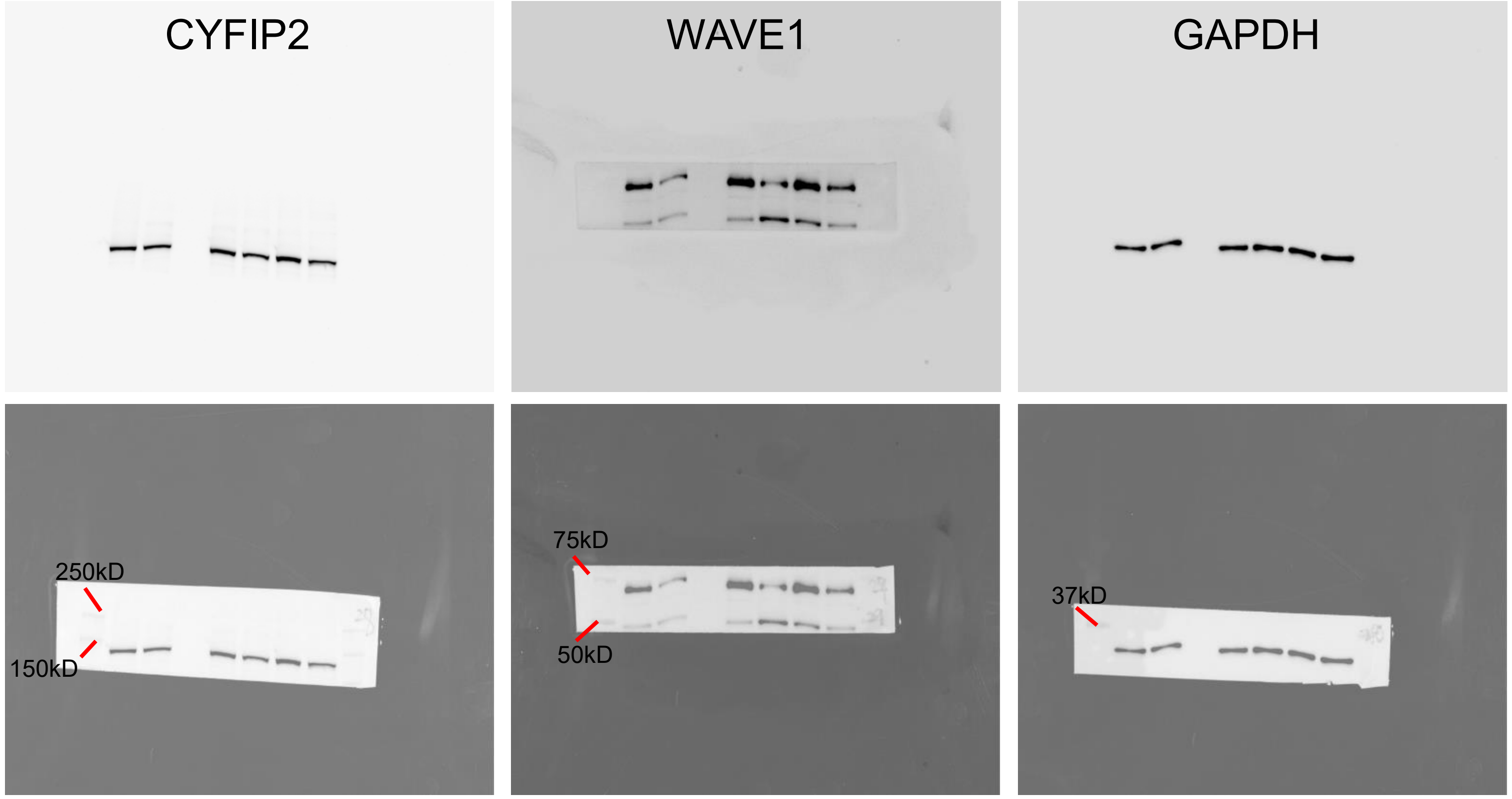

WT6, KI6; X; WT7, KI7; WT8, KI8

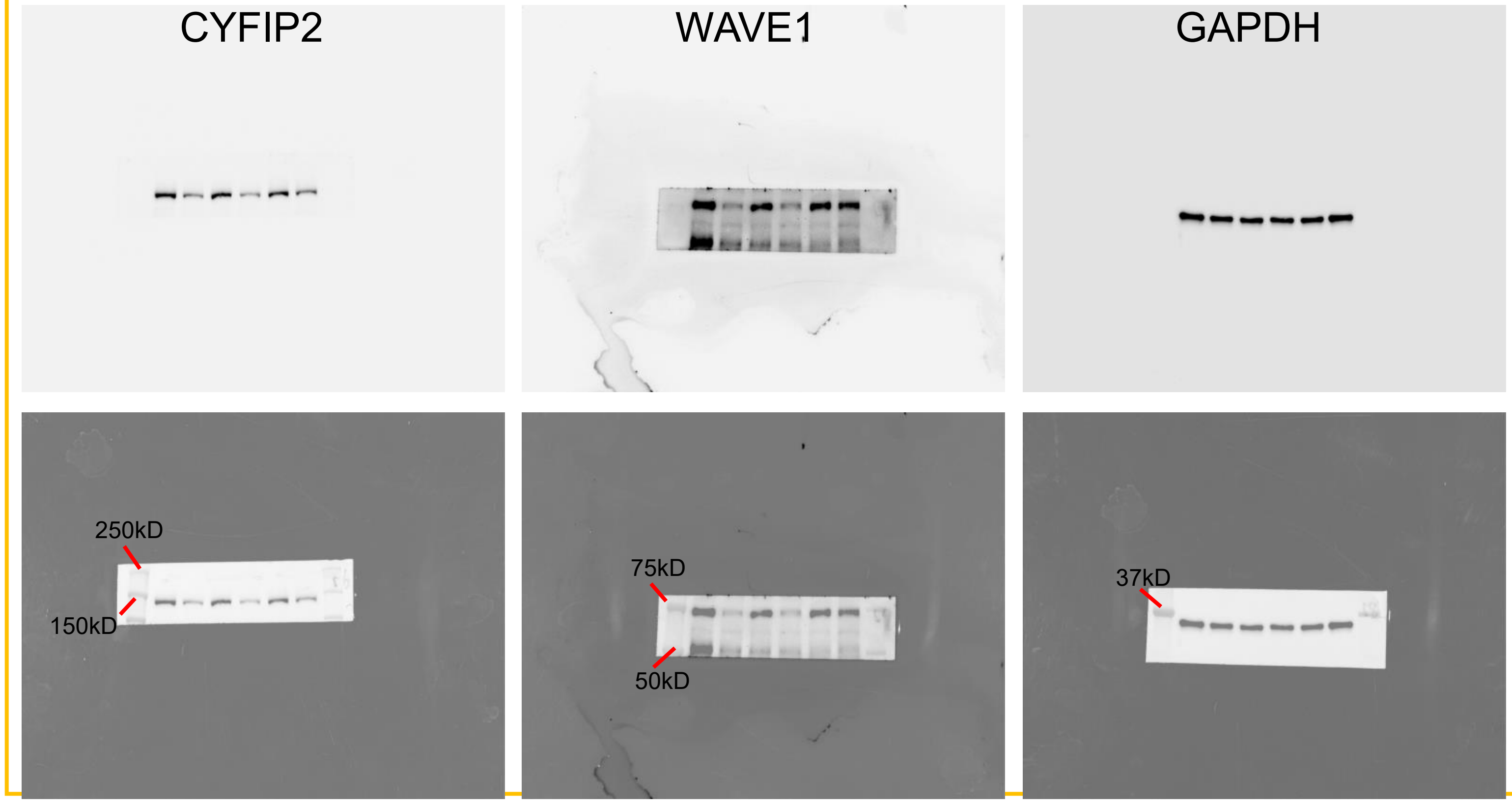

28wk_CTX

Sample loading:
WT1 KI1; WT2, KI2; WT3, KI3; WT4, KI4

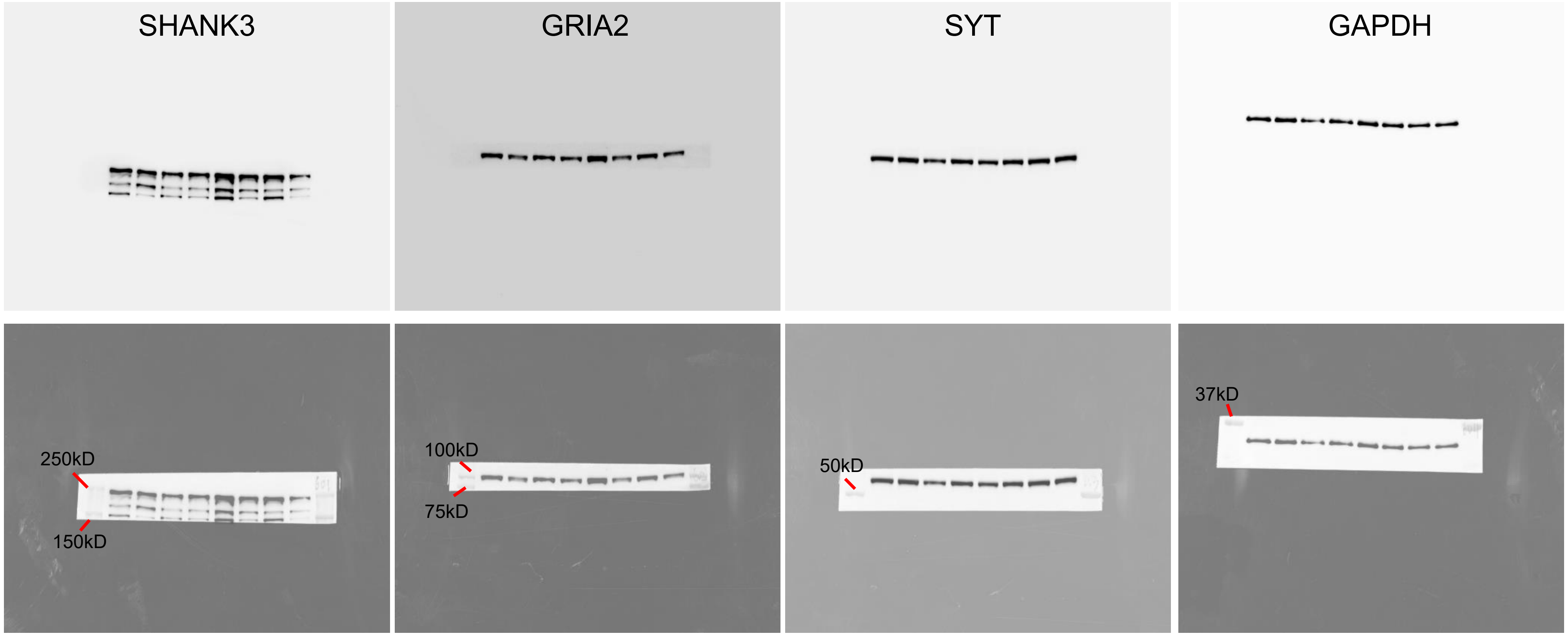

WT5, WT6, KI5; X, WT7, KI6; WT8, KI7

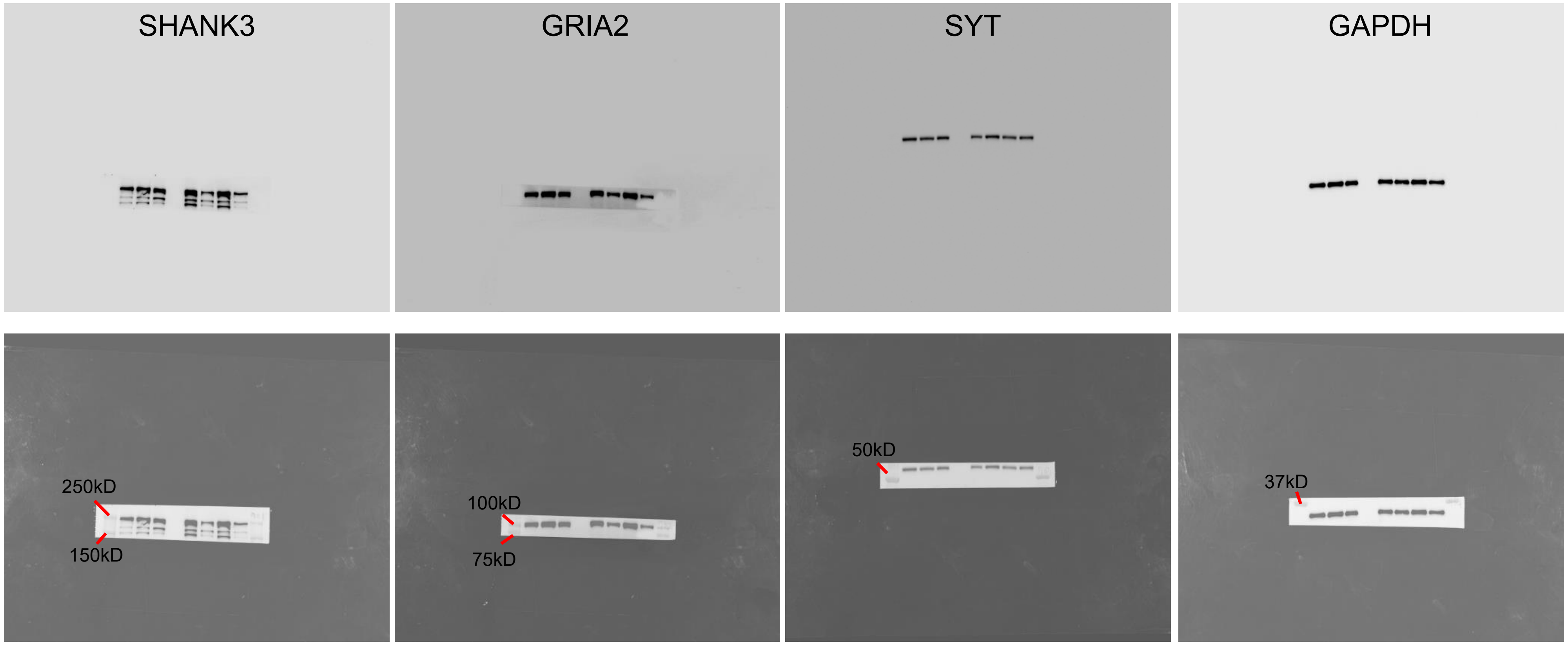

28wk_CTX

Sample loading:

WT1 KI1; WT2, KI2; WT3, KI3; WT4, KI4

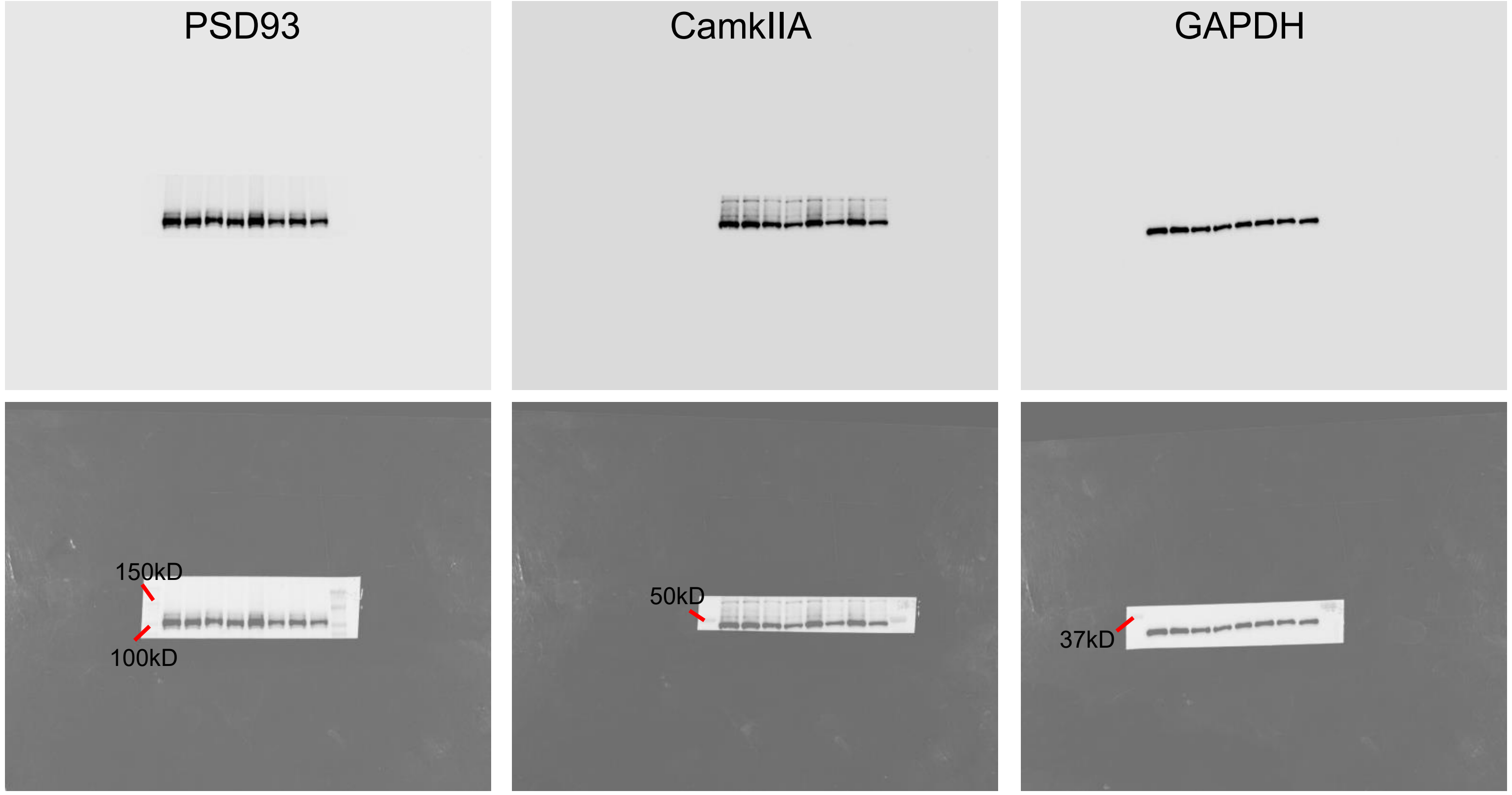

WT5, WT6, KI5; X, WT7, KI6; WT8, KI7

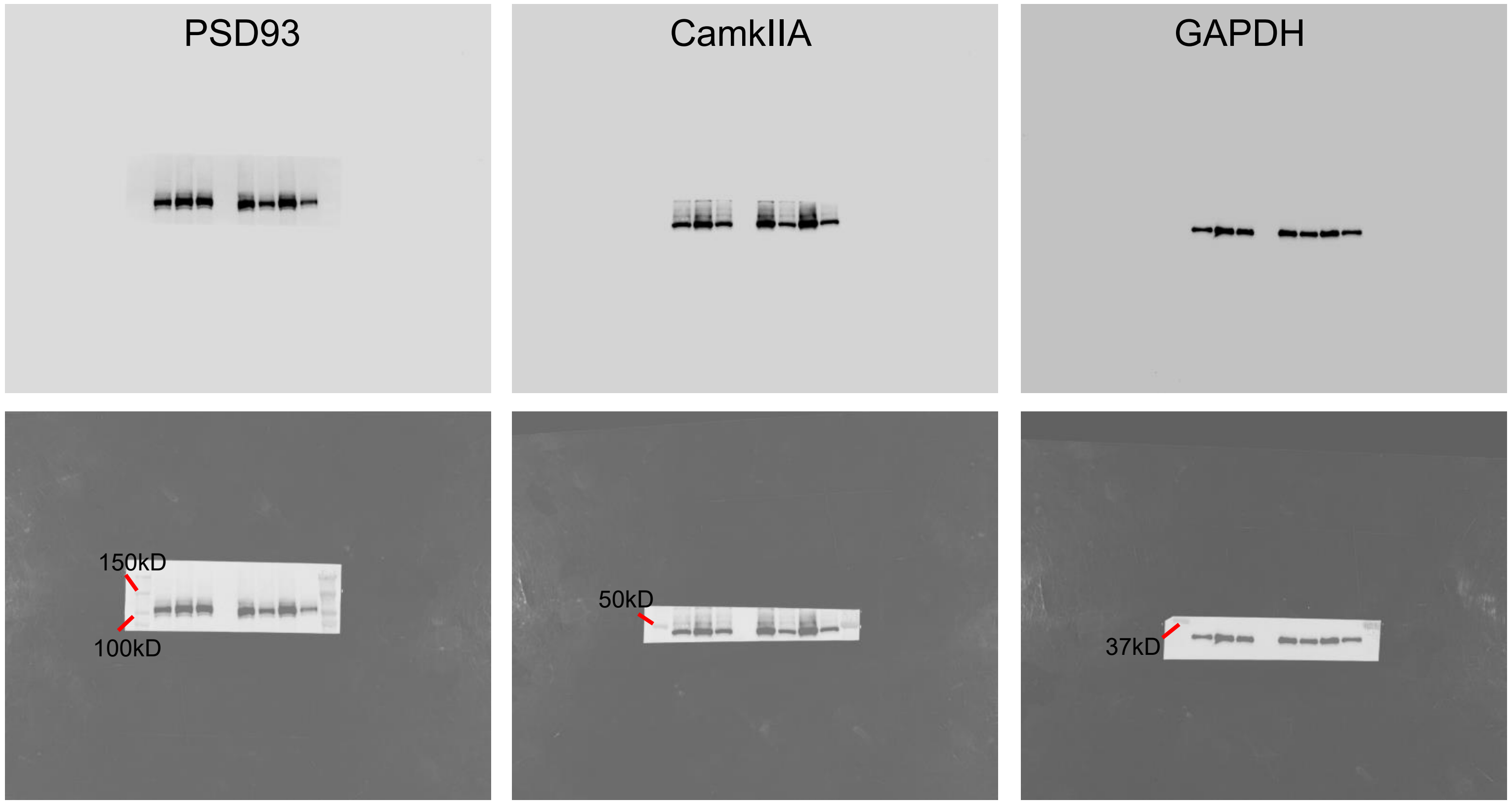

28wk_CTX

Sample loading:

WT1 KI1; WT2, KI2; WT3, KI3; WT4, KI4

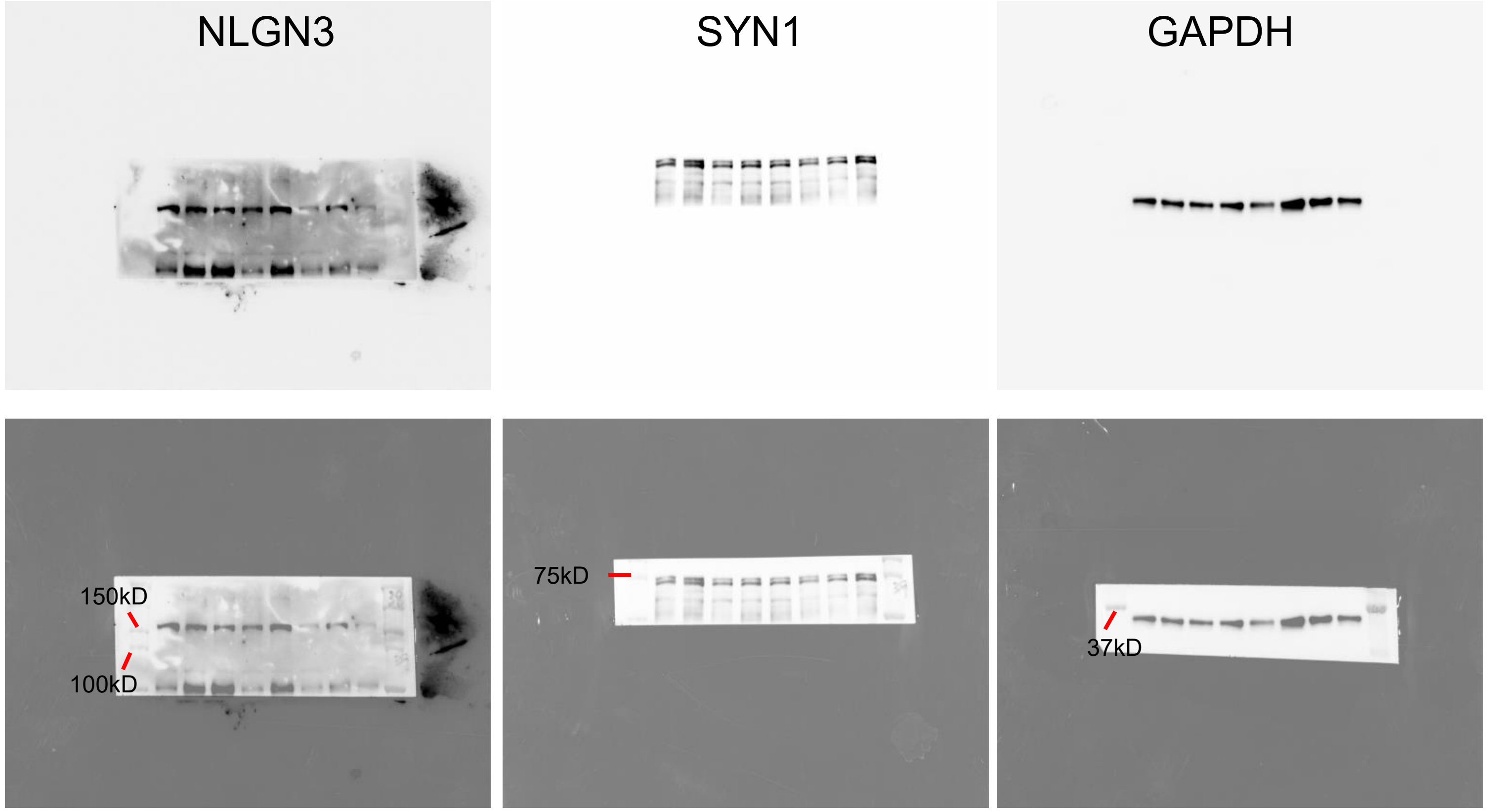

WT5, WT6, KI5; WT7, KI6; WT8, KI7

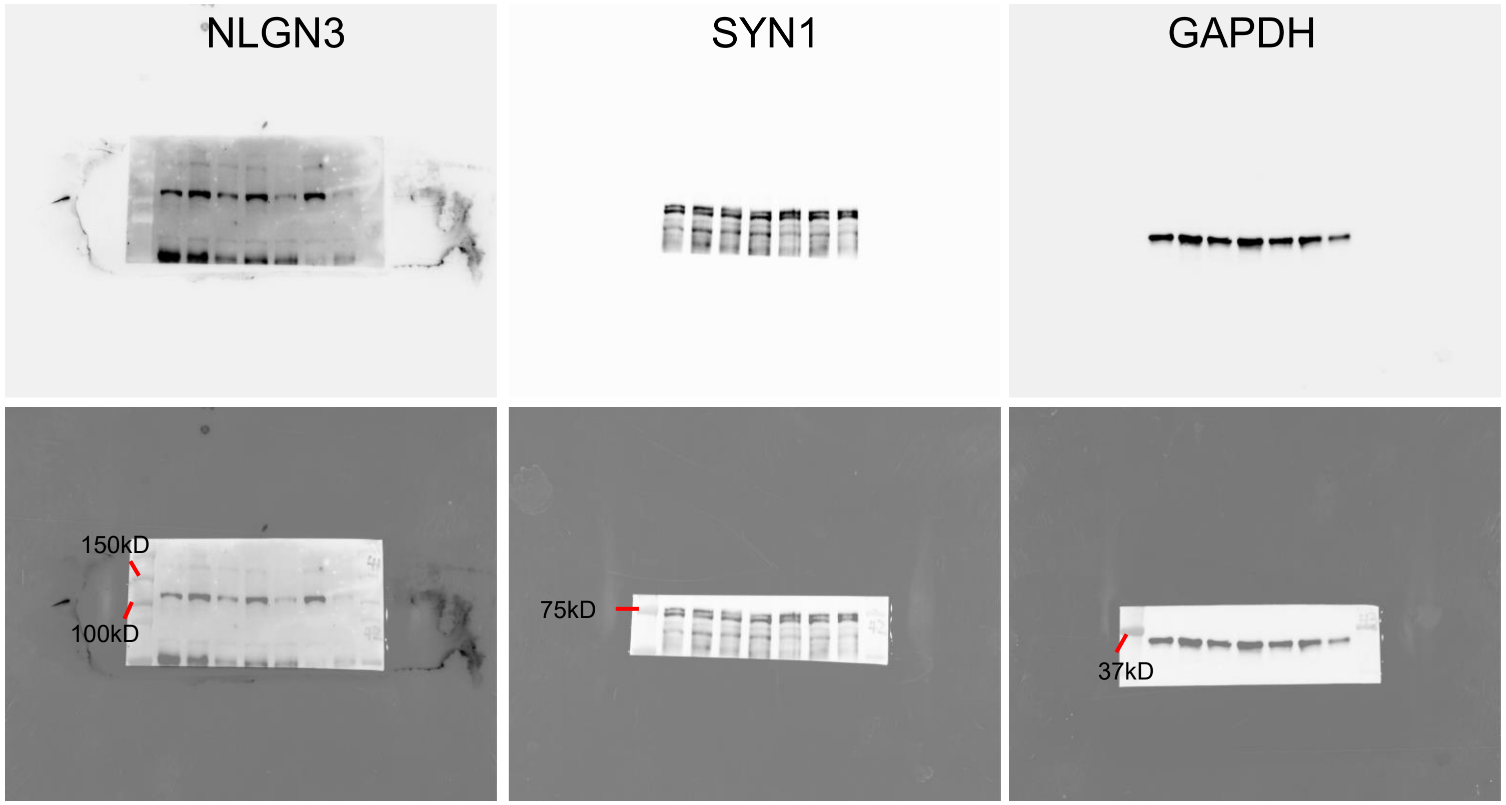

28wk_CTX

Sample loading:
WT1 KI1; WT2, KI2; WT3, KI3; WT4, KI4

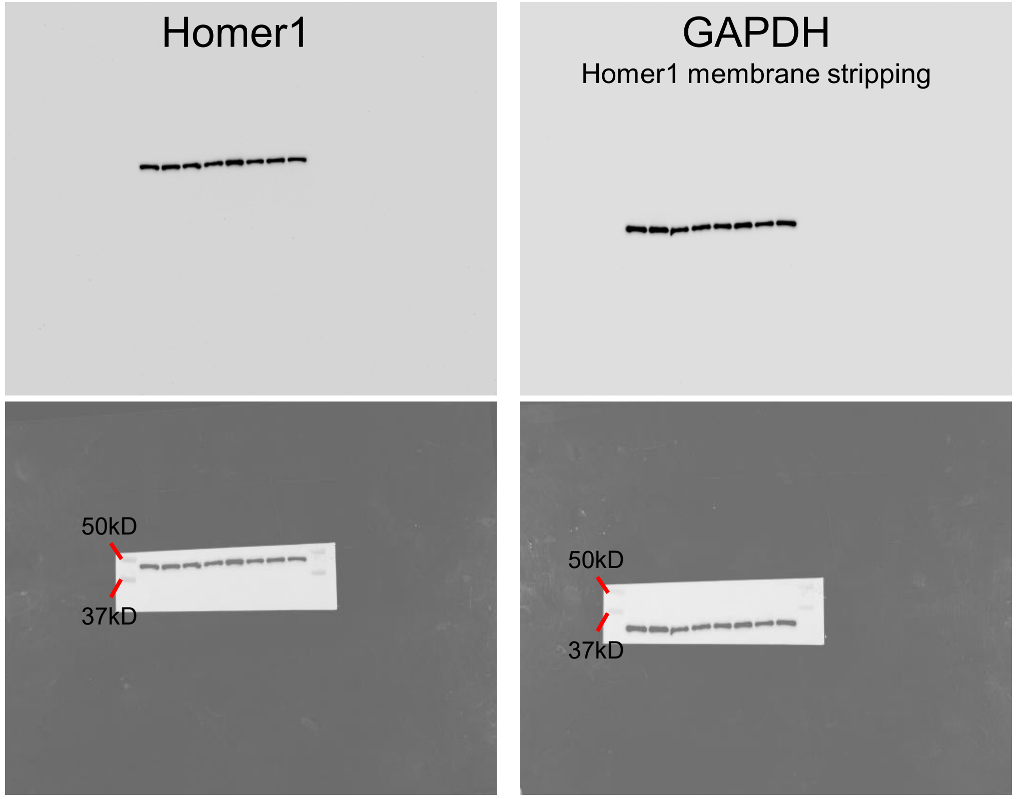

WT5, WT6, KI5; X, WT7, KI6; WT8, KI7

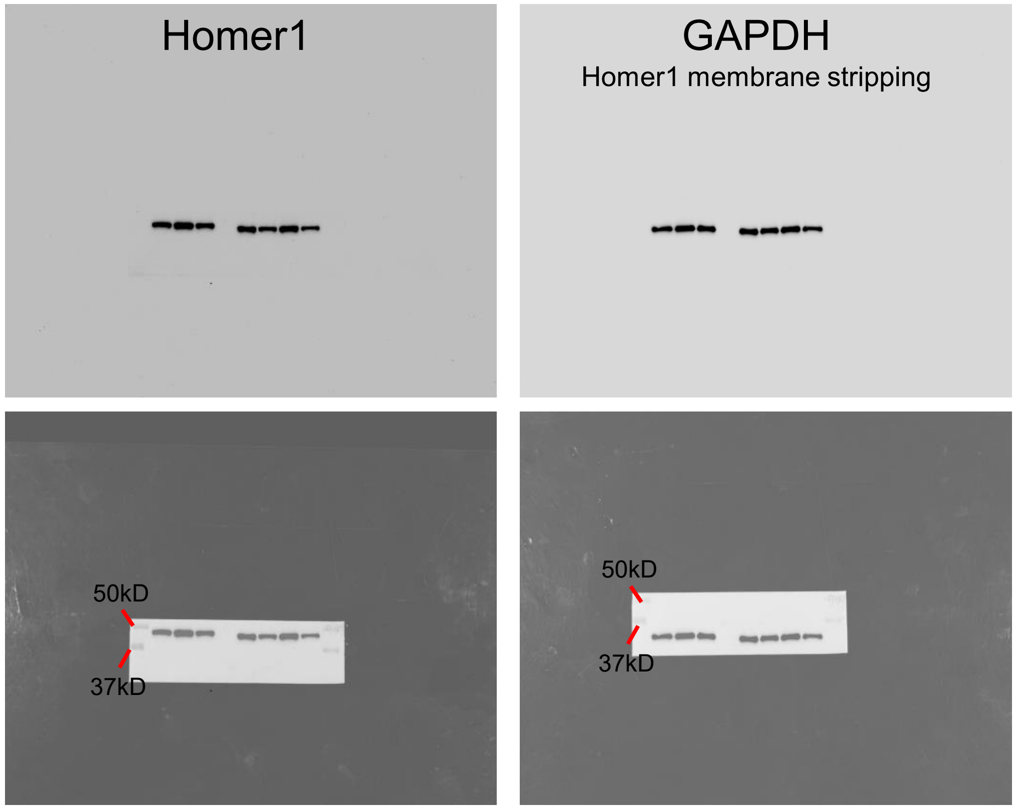

28wk_CTX

Sample loading:
WT1 KI1; WT2, KI2; WT3, KI3; WT4, KI4

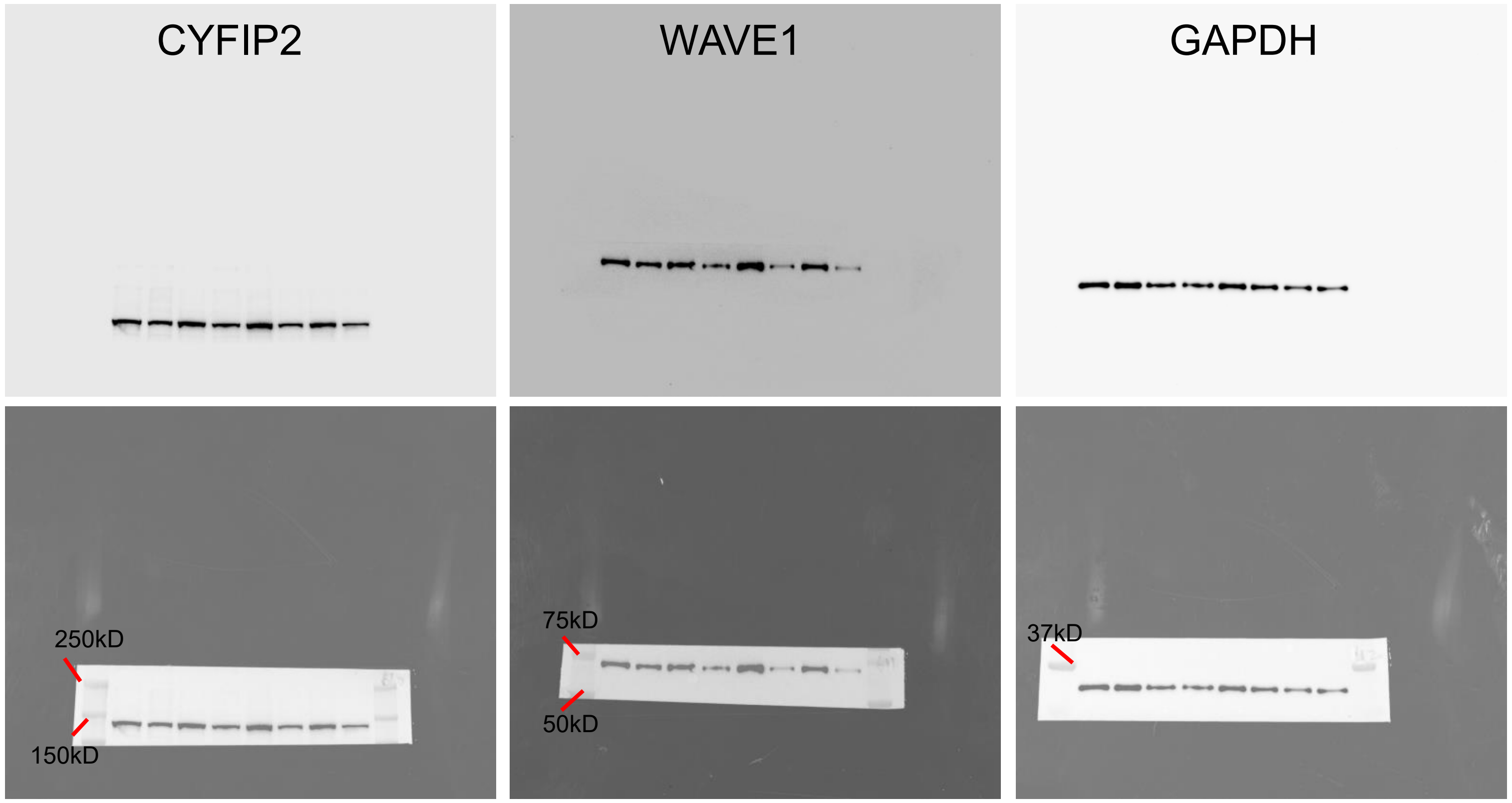

WT5, WT6, KI5; X, WT7, KI6; WT8, KI7

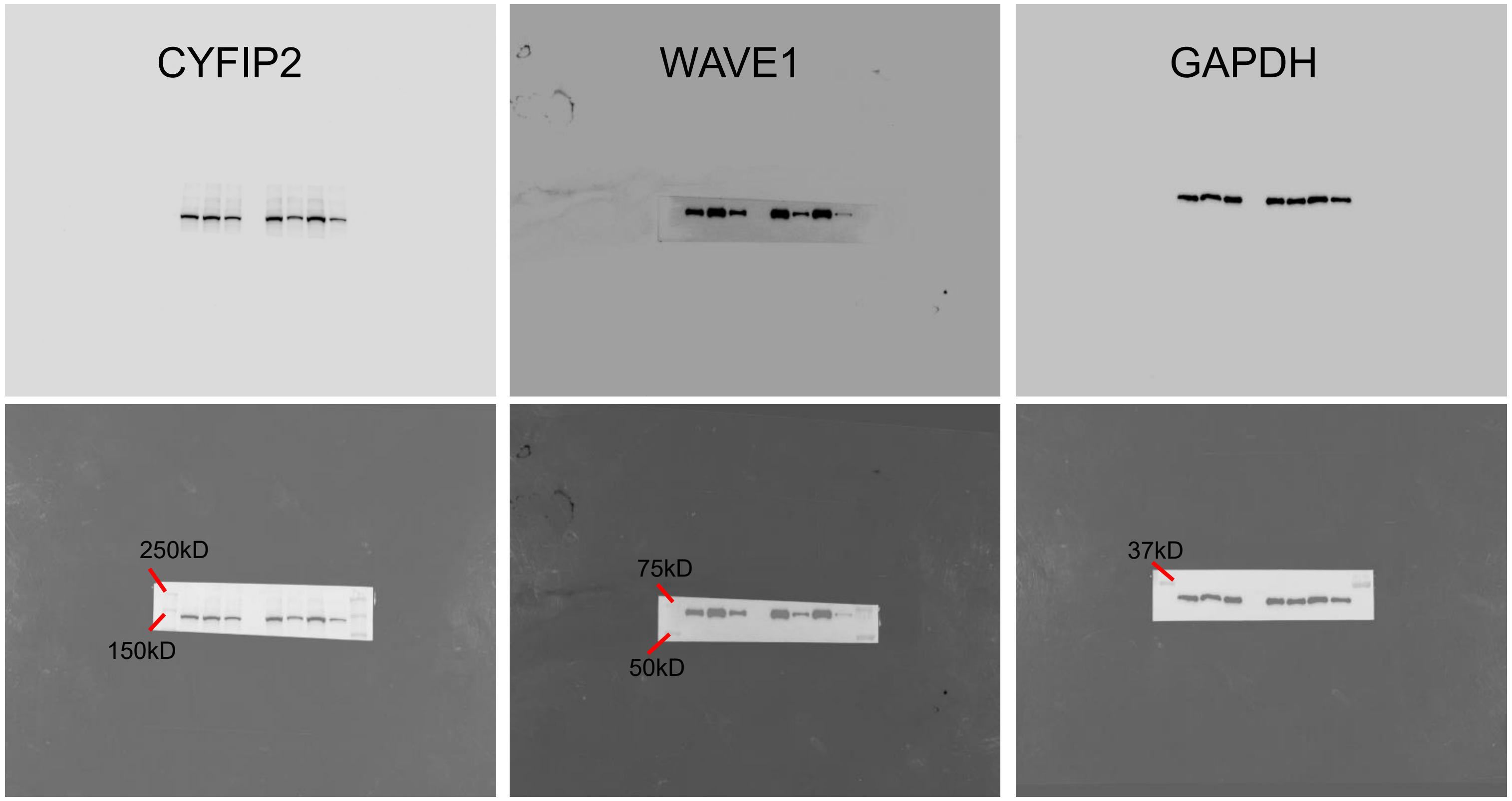

1wk_HIP

Sample loading:
WT1 KI1; WT2, KI2; WT3, KI3

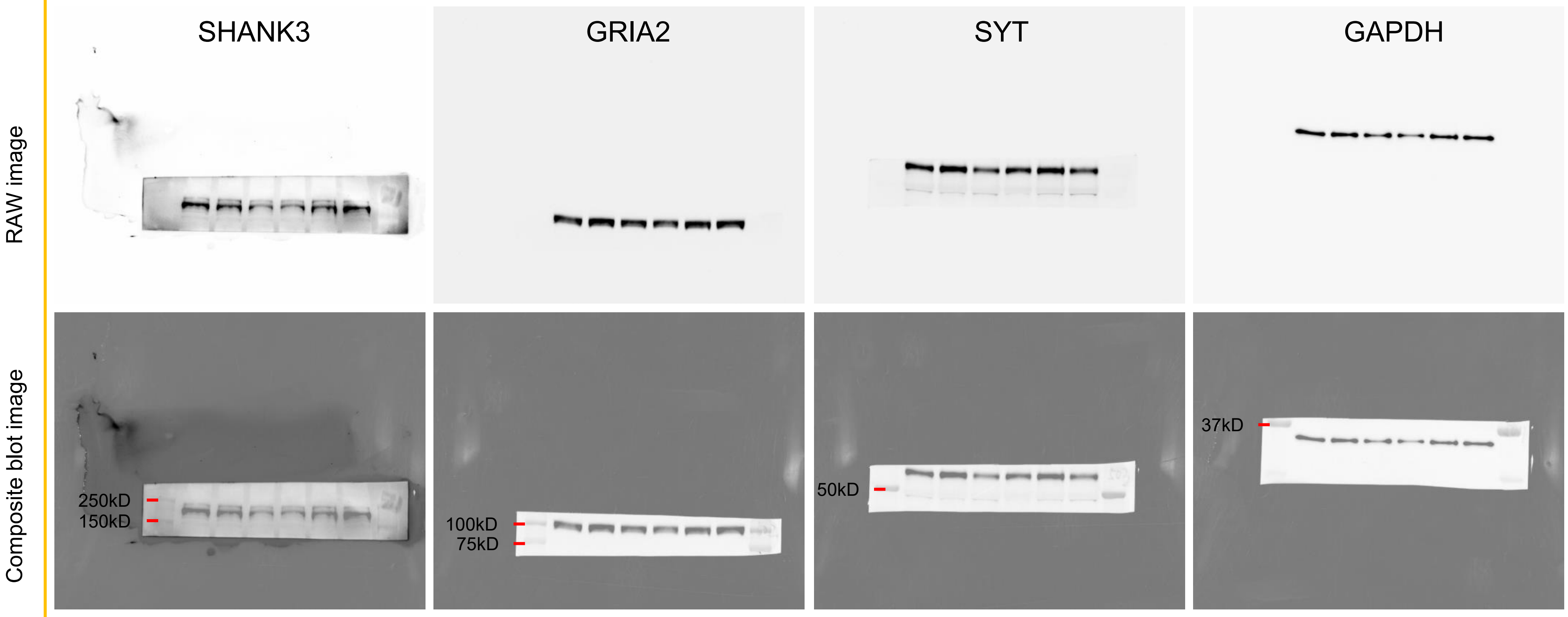

WT4, KI4; WT5 KI5; WT6, KI6; WT7, KI7

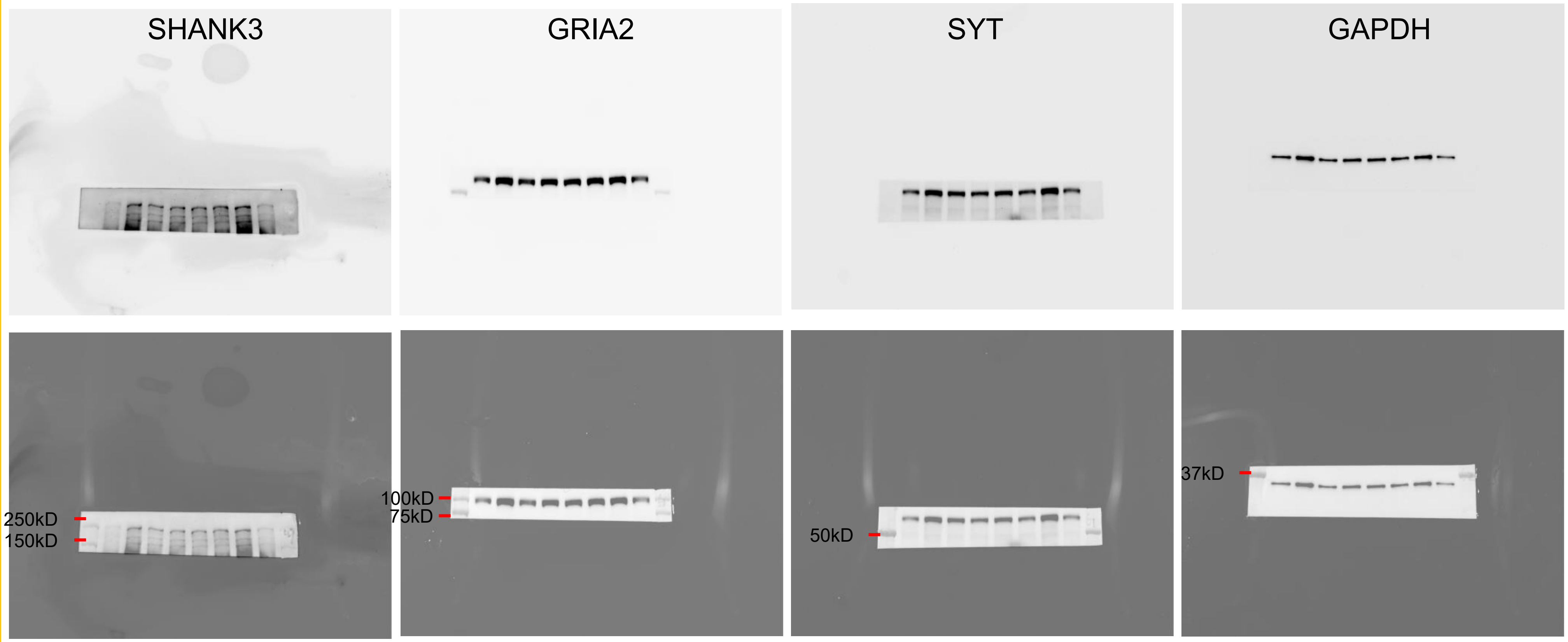

1wk_HIP

Sample loading:
WT1 KI1; WT2, KI2; WT3, KI3

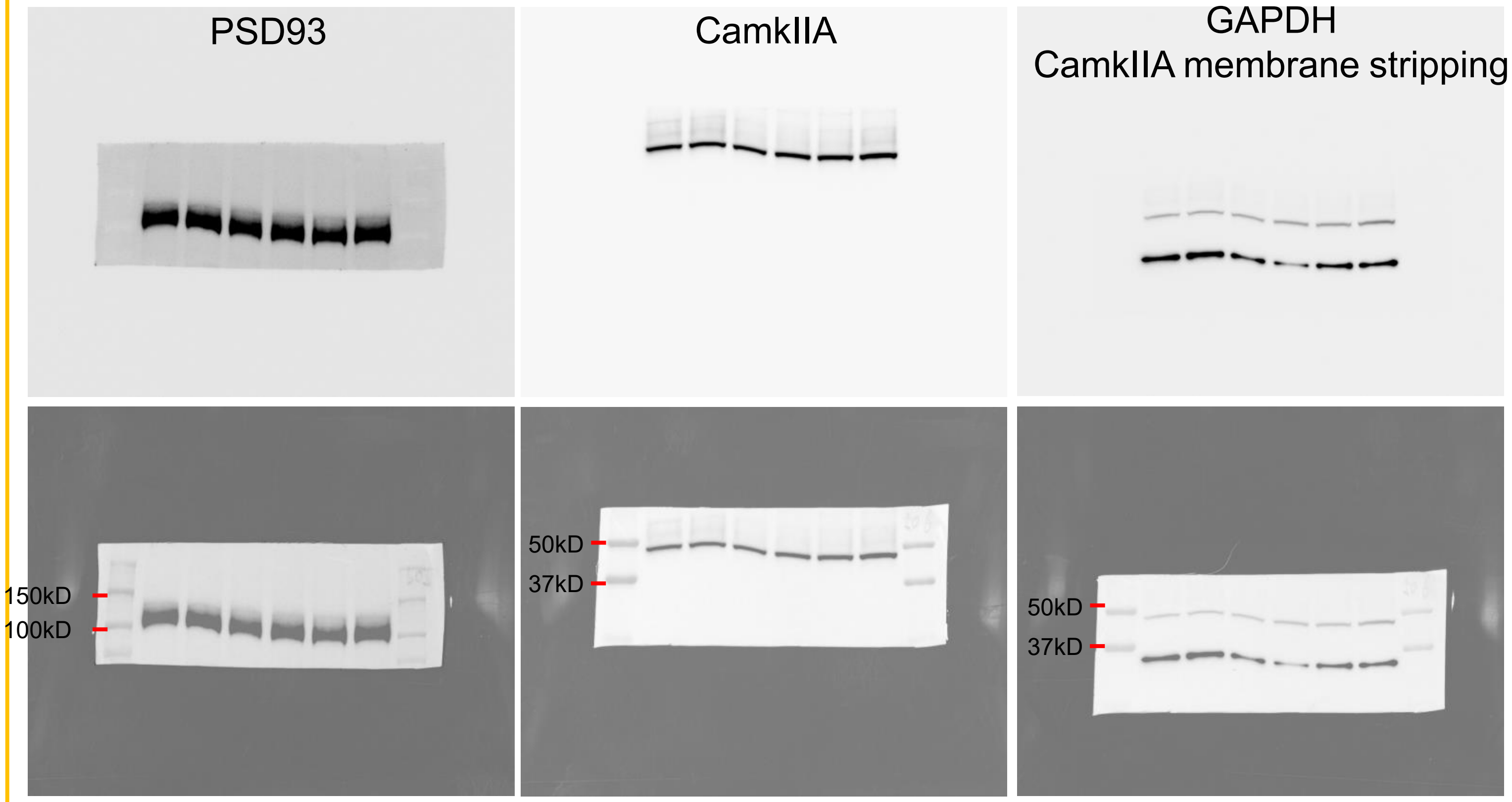

WT4, KI4; WT5 KI5; WT6, KI6; WT7, KI7

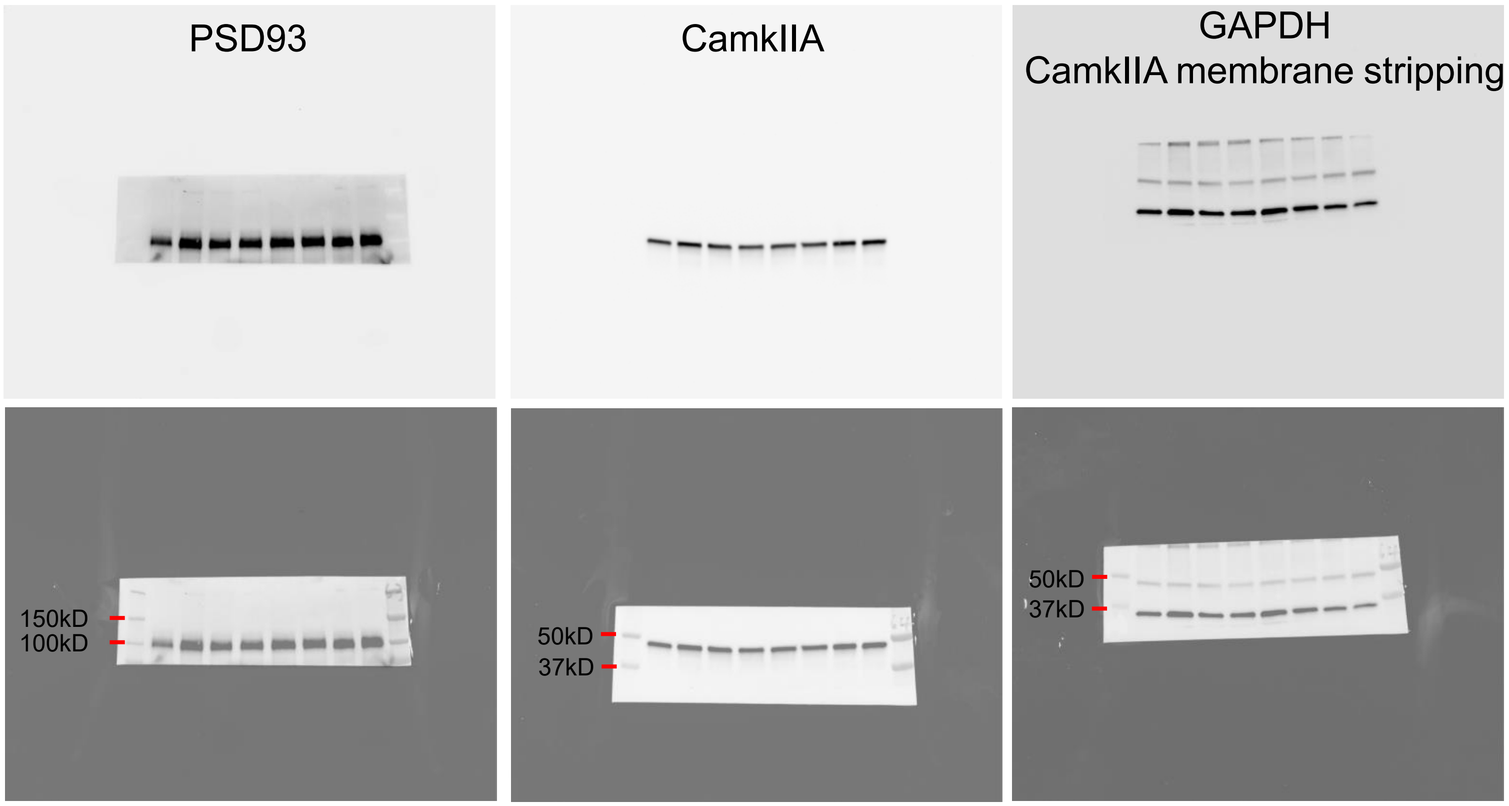

1wk_HIP

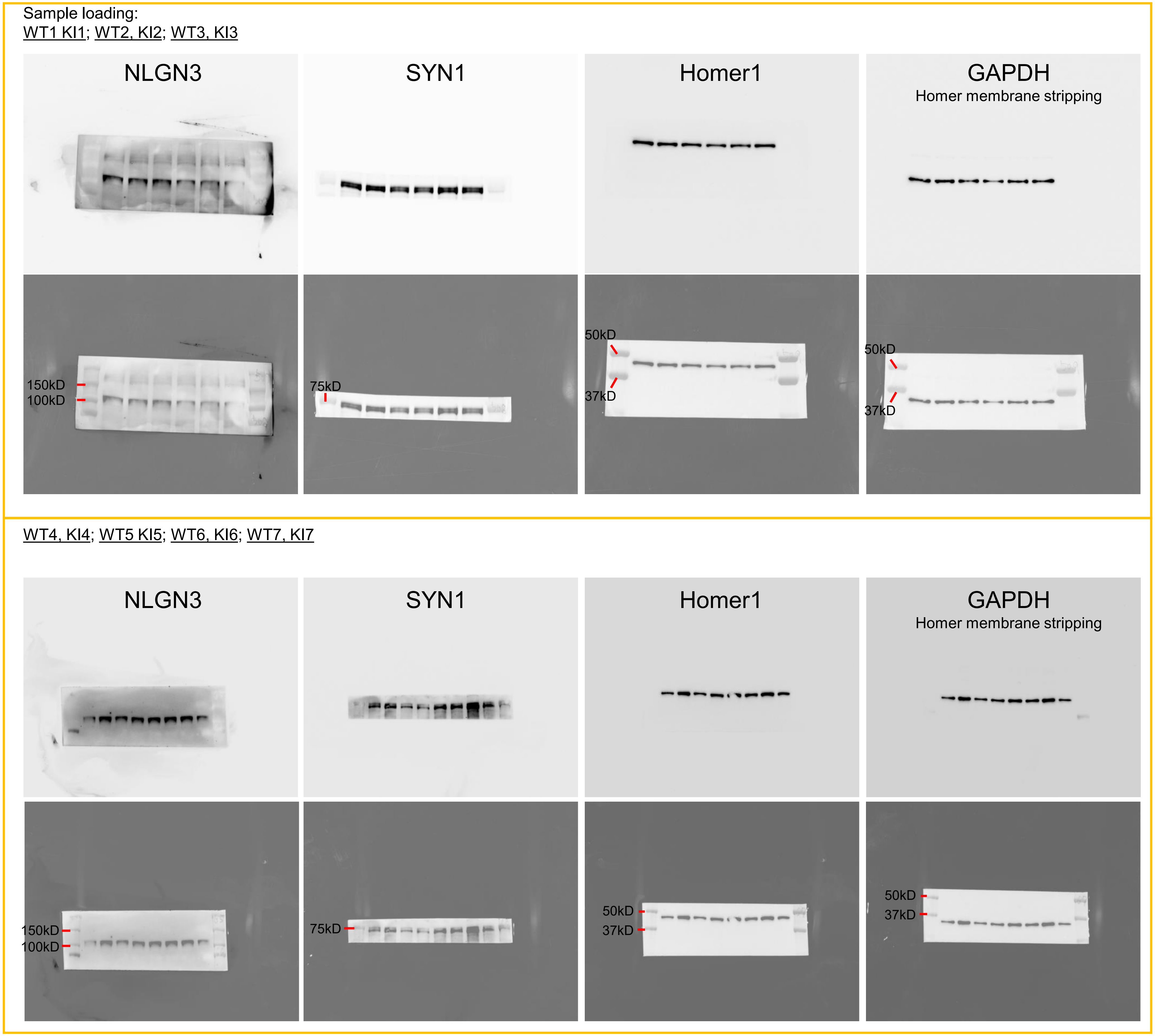

Sample loading:
WT1 KI1; WT2, KI2; WT3, KI3
NLGN3
SYN1
Homer1
GAPDH
Homer membrane stripping
150kD
100kD
75kD
50kD
37kD
50kD
37kD
WT4, KI4; WT5 KI5; WT6, KI6; WT7, KI7
NLGN3
SYN1
Homer1
GAPDH
Homer membrane stripping
150kD
100kD
75kD
50kD
37kD
50kD
37kD

1wk_HIP

Sample loading:
WT1 KI1; WT2, KI2; WT3, KI3

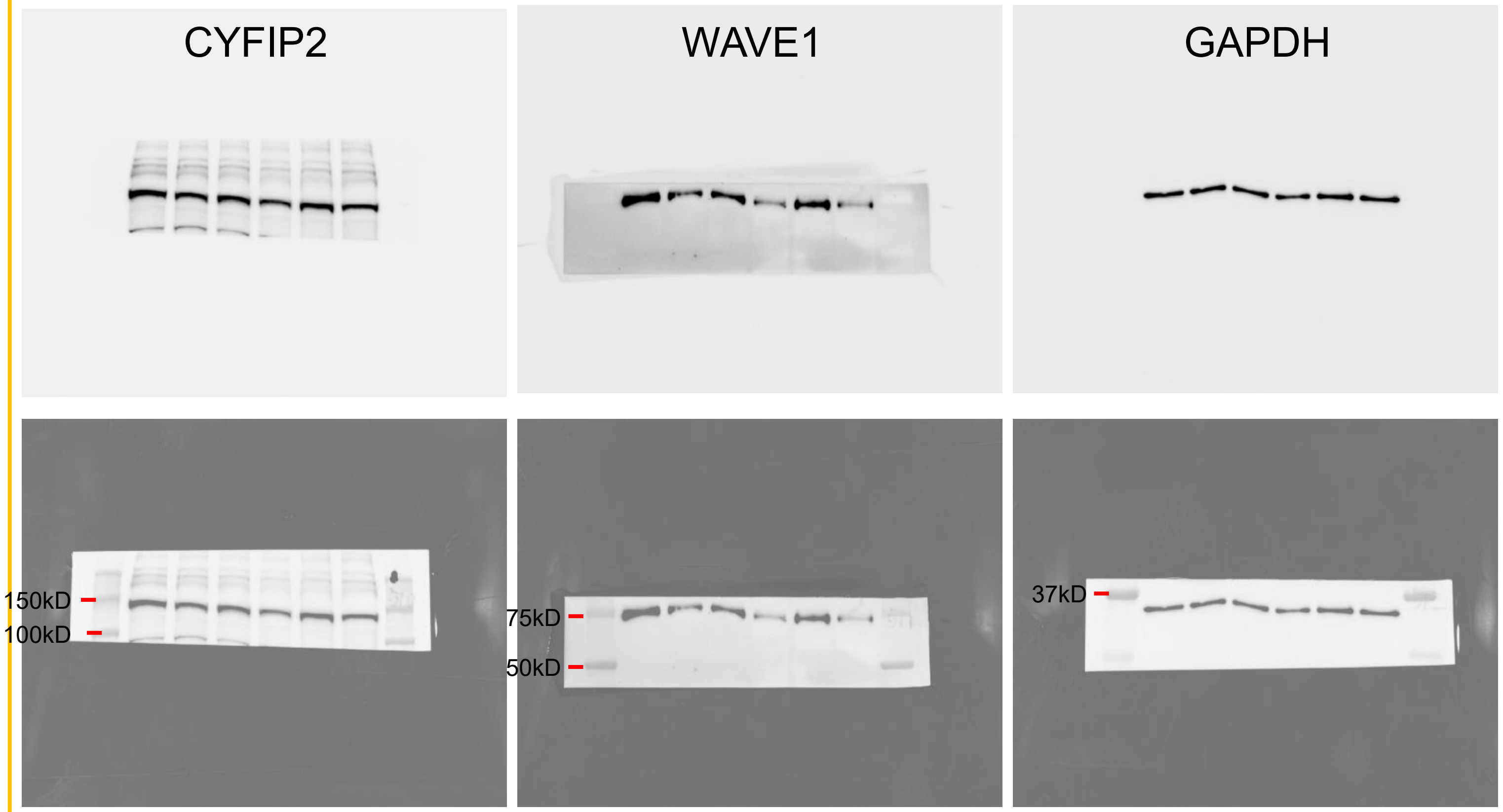

WT4, KI4; WT5 KI5; WT6, KI6; WT7, KI7

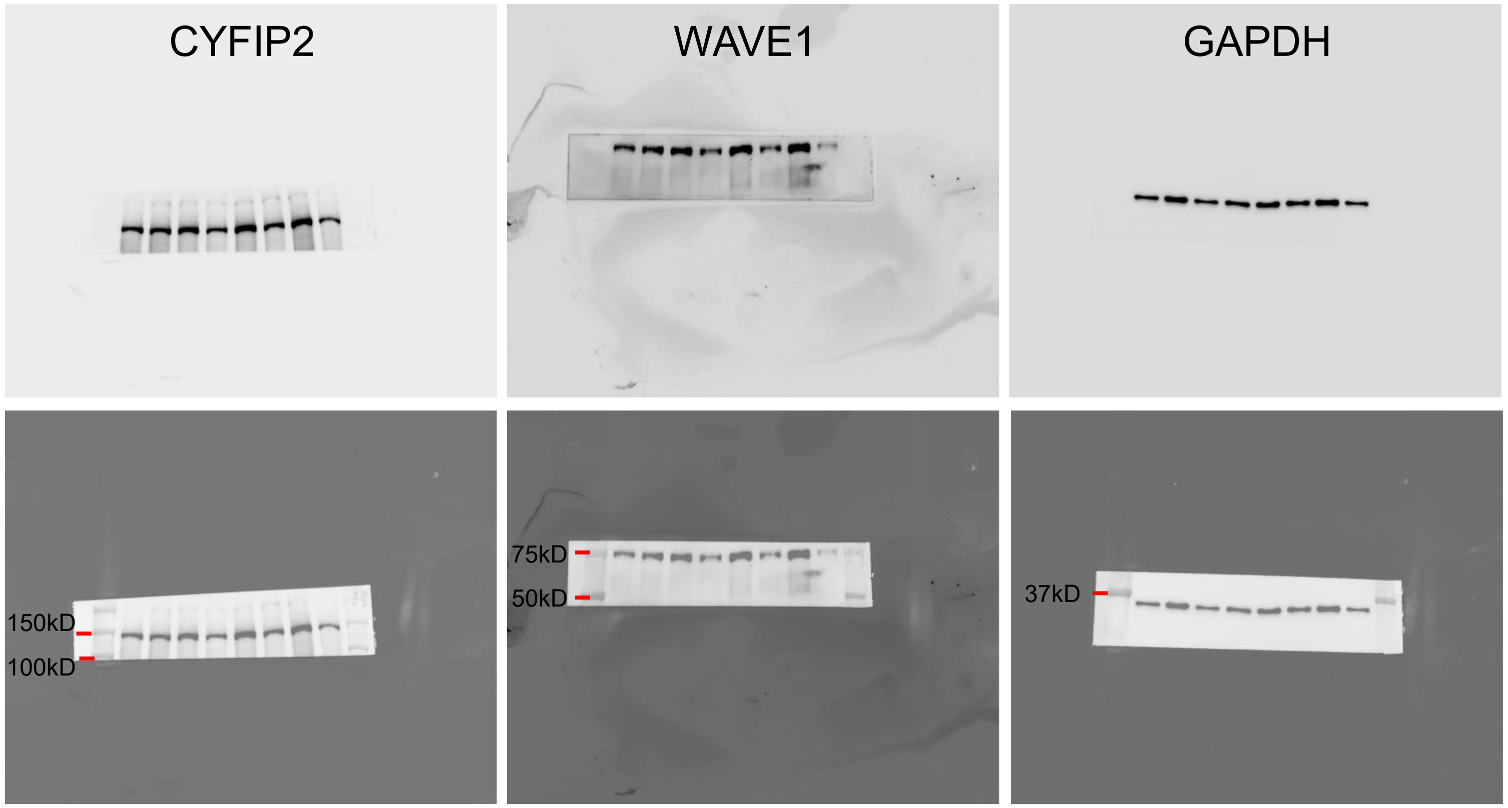

7wk_HIP

Sample loading:

WT1 KI1; WT2, KI2; WT3, KI3; WT4, KI4

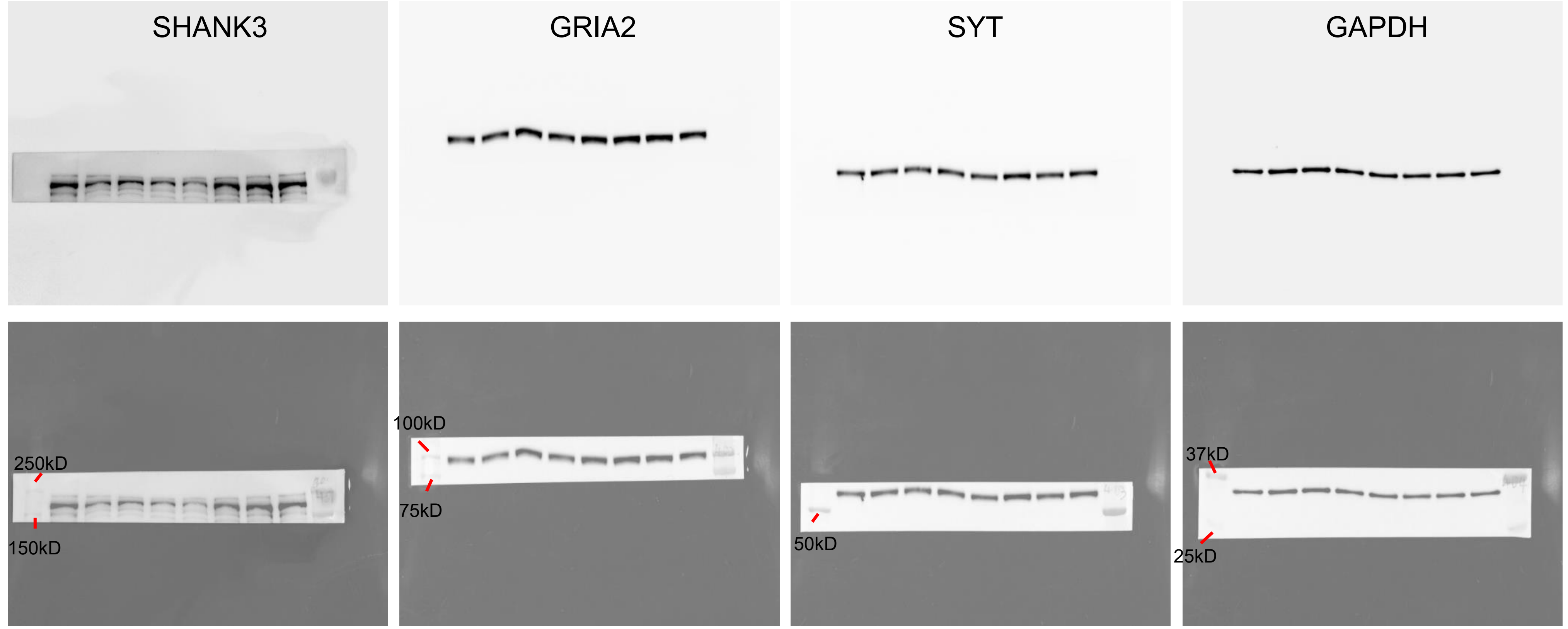

WT5 KI5; WT6, KI6; KI7

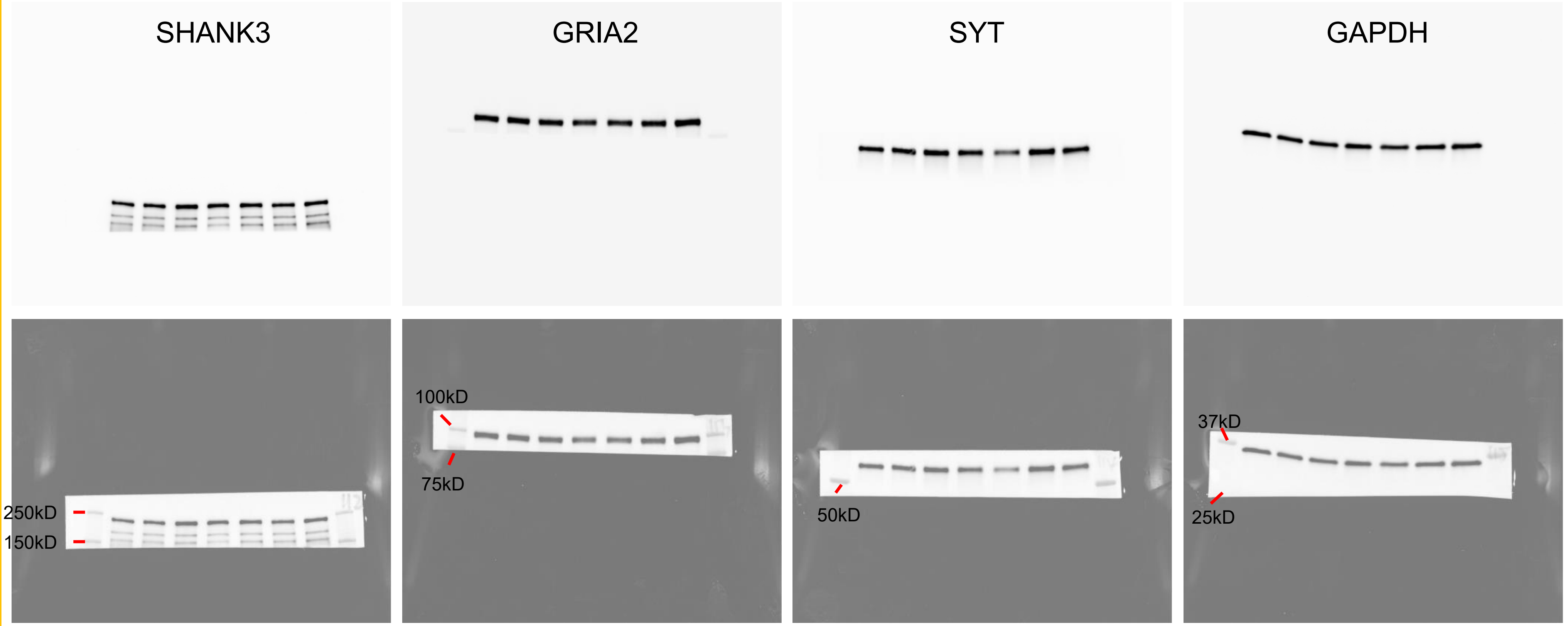

7wk_HIP

Sample loading:

WT1 KI1; WT2, KI2; WT3, KI3; WT4, KI4

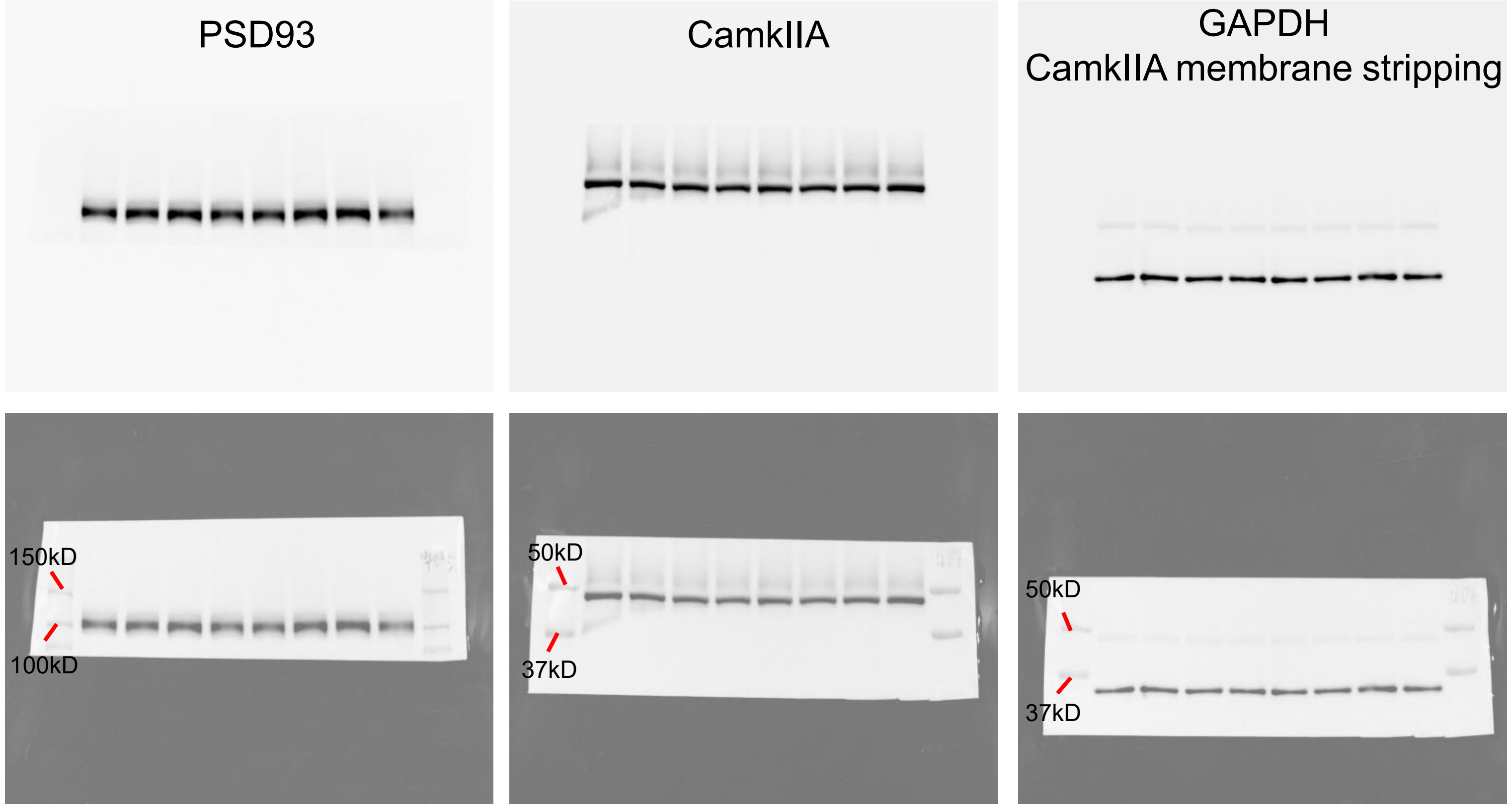

WT5 KI5; WT6, KI6; KI7

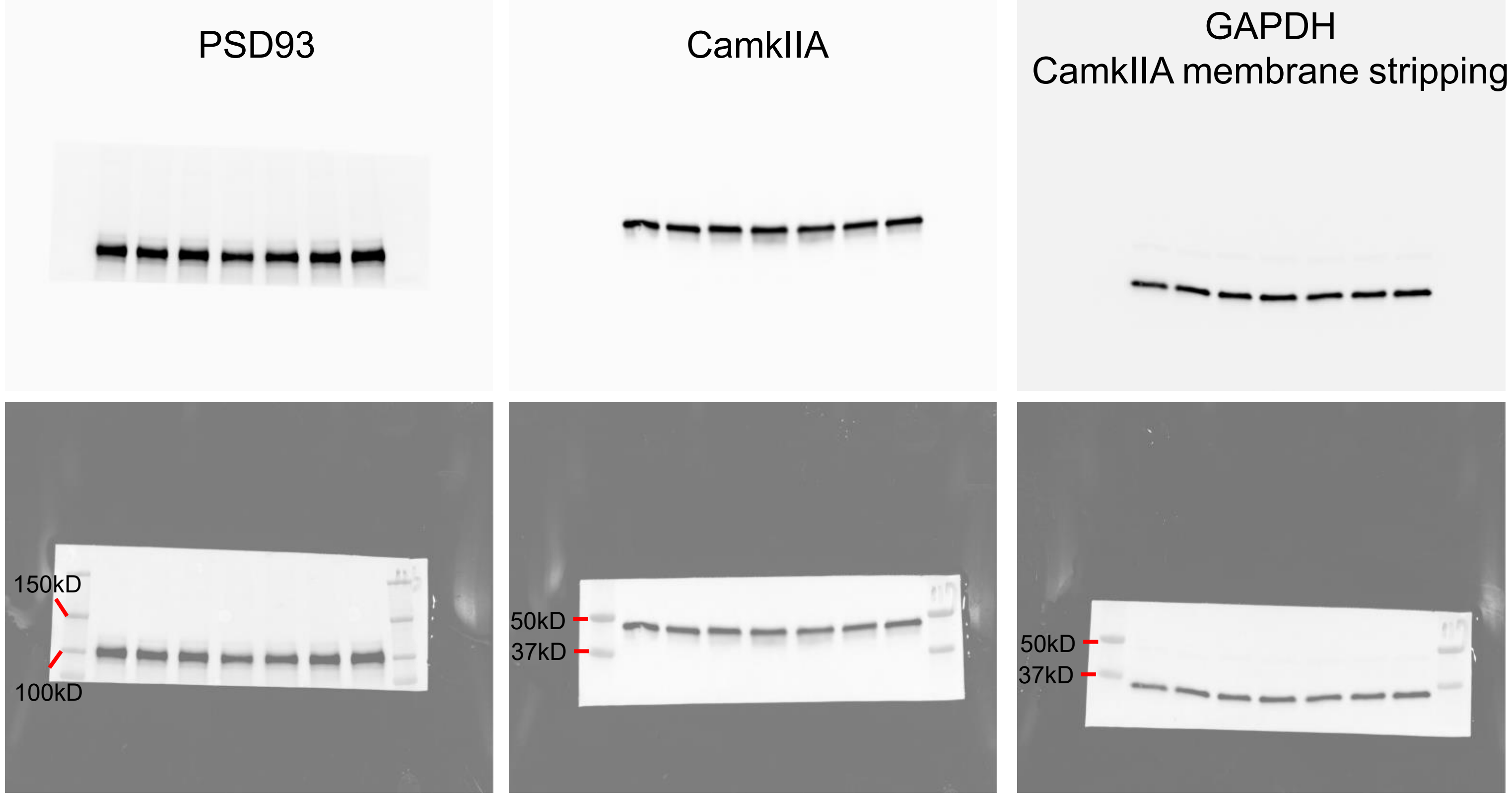

7wk_HIP

Sample loading:
WT1 KI1; WT2, KI2; WT3, KI3; WT4, KI4

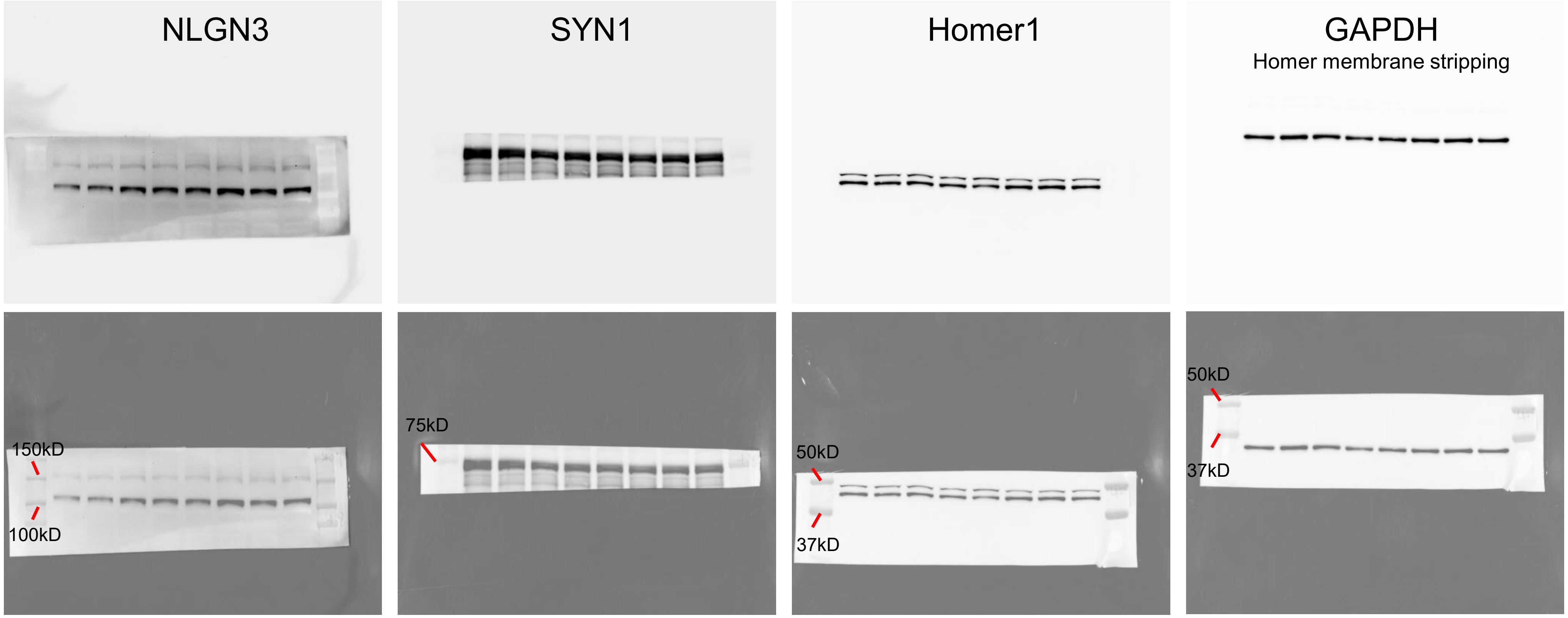

WT5 KI5; WT6, KI6; KI7

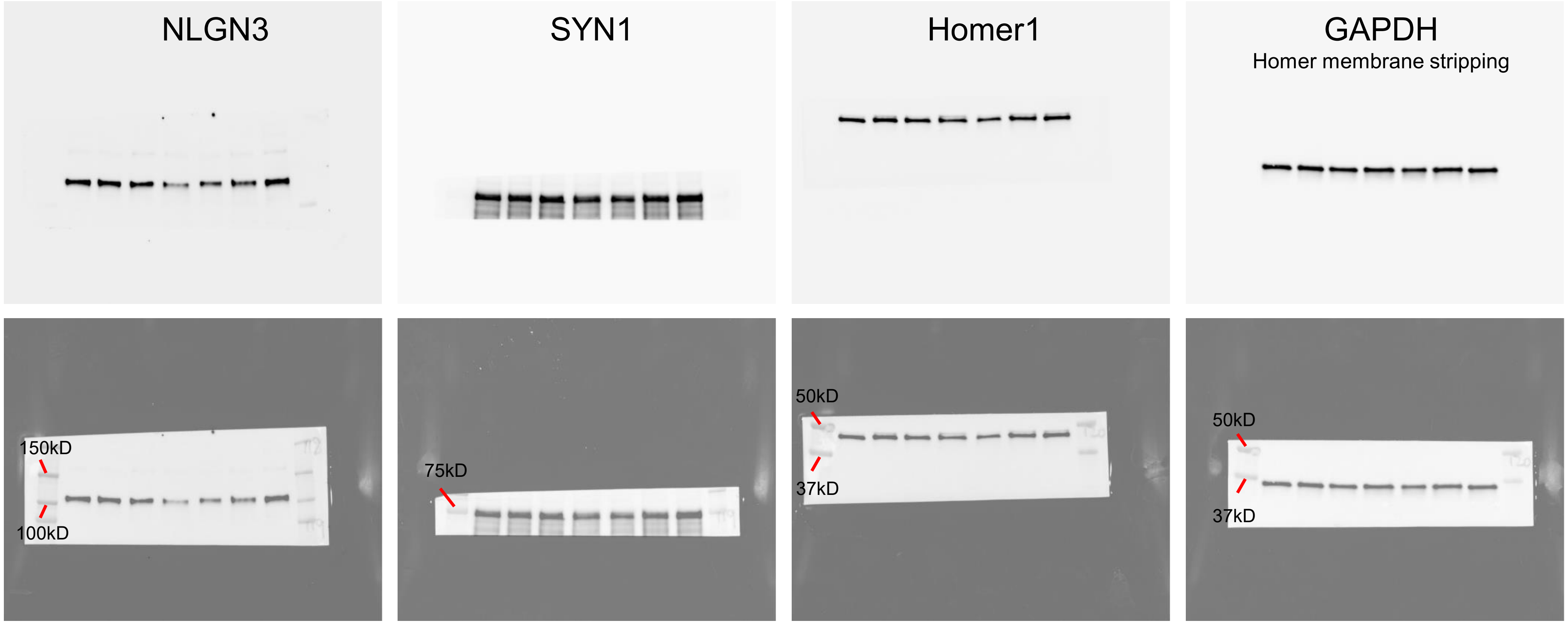

7wk_HIP

Sample loading:
WT1 KI1; WT2, KI2; WT3, KI3; WT4, KI4

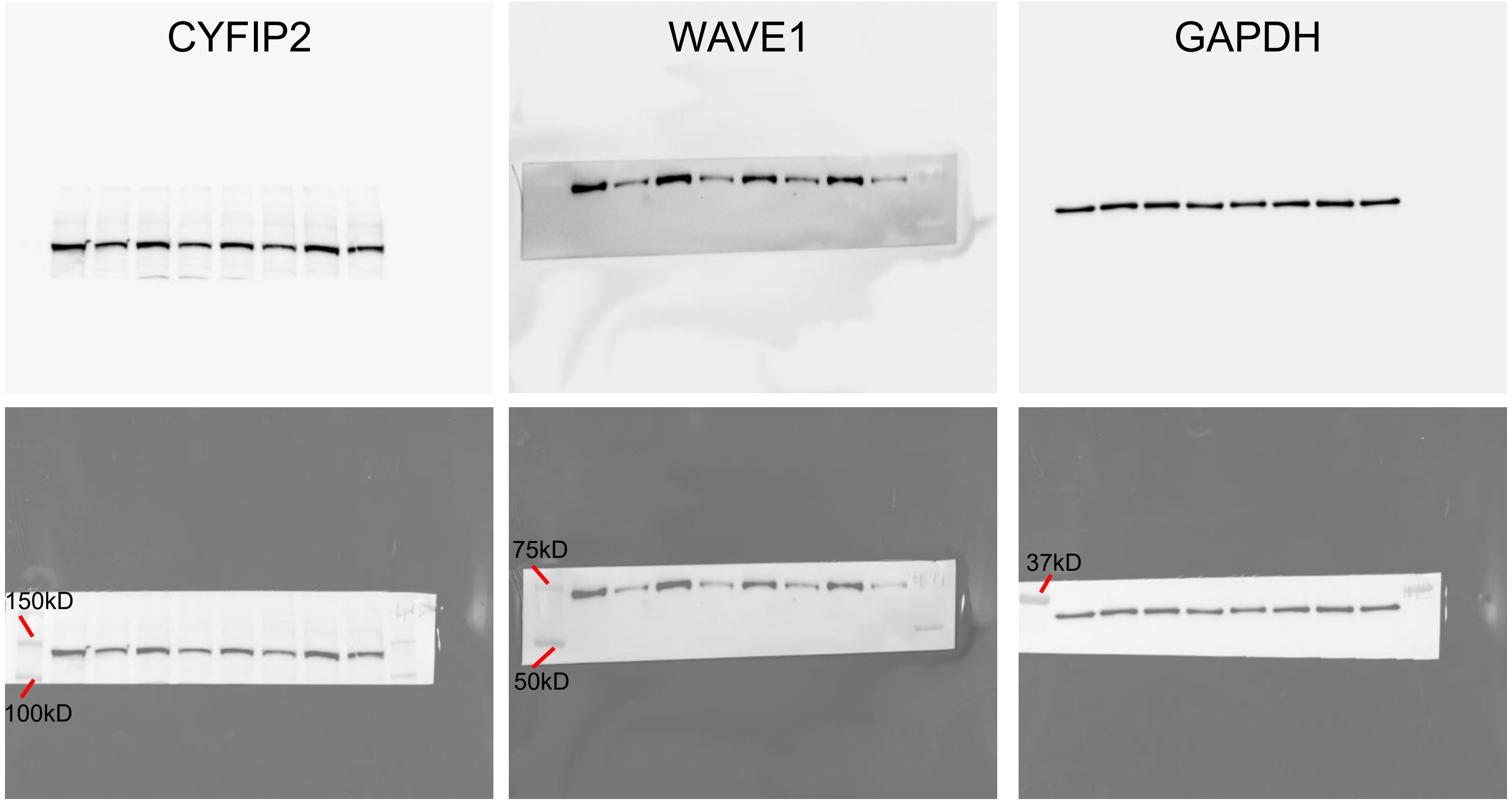

WT5 KI5; WT6, KI6; KI7

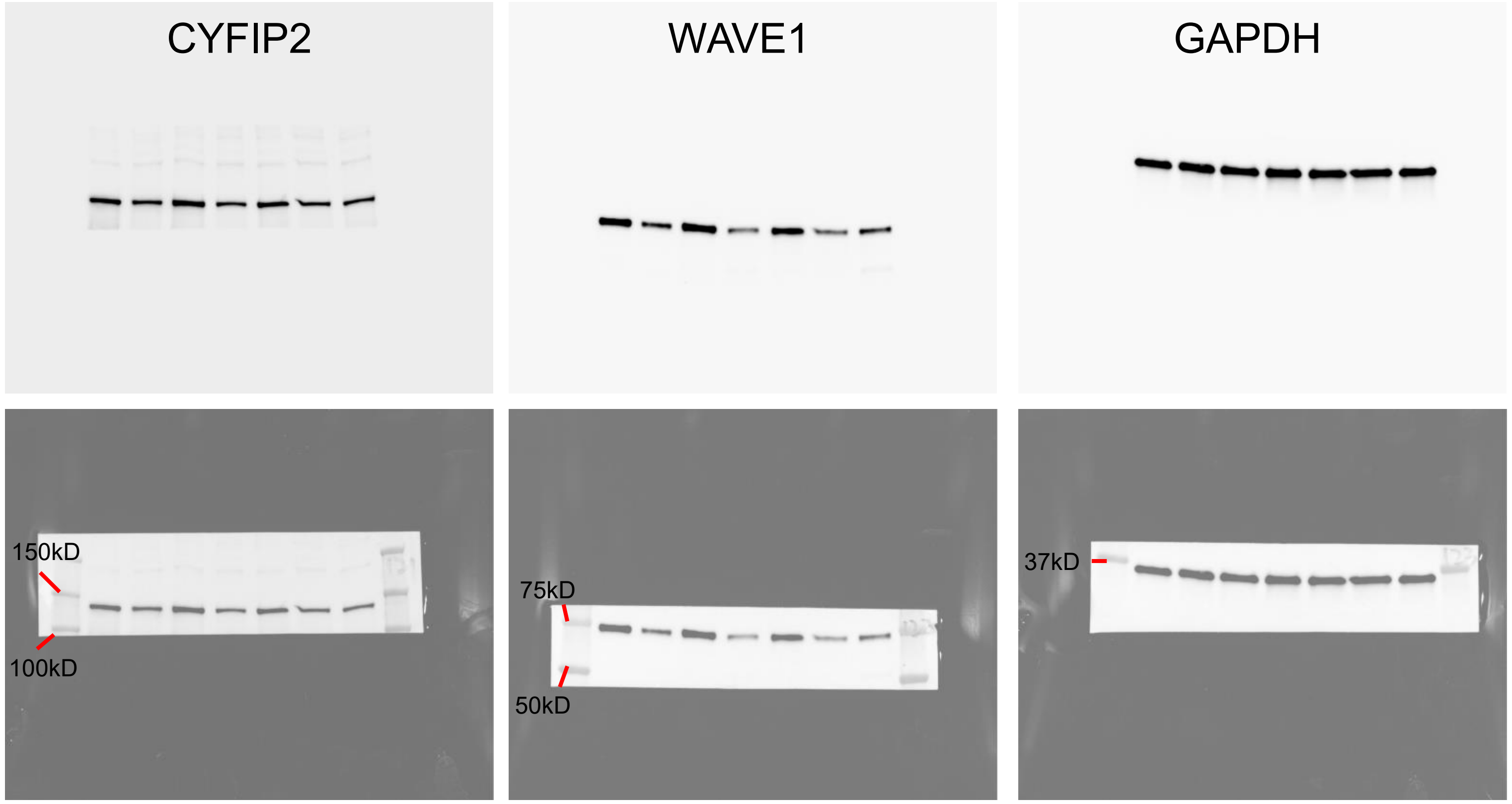

14wk_HIP

Sample loading:
WT1 KI1; WT2, KI2;

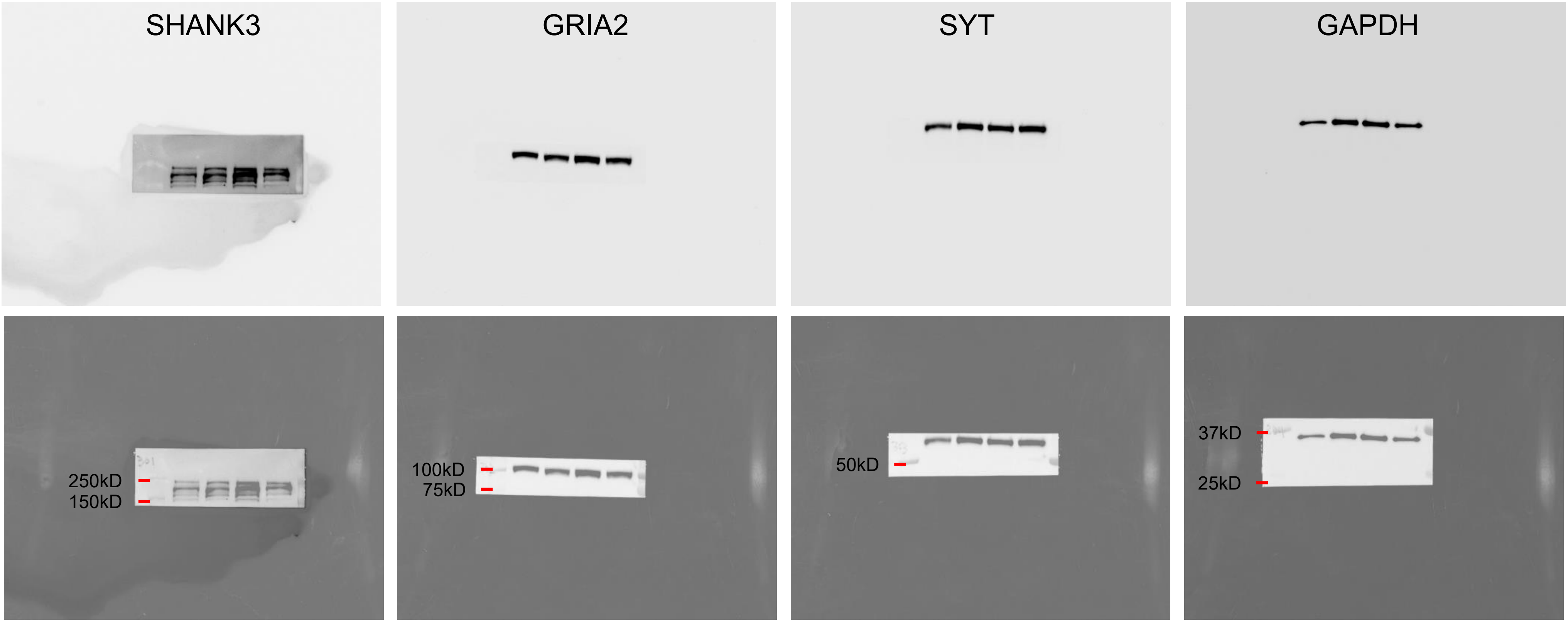

14wk_HIP

Sample loading:
WT3 KI3; X; WT4, KI4; WT5, KI5;

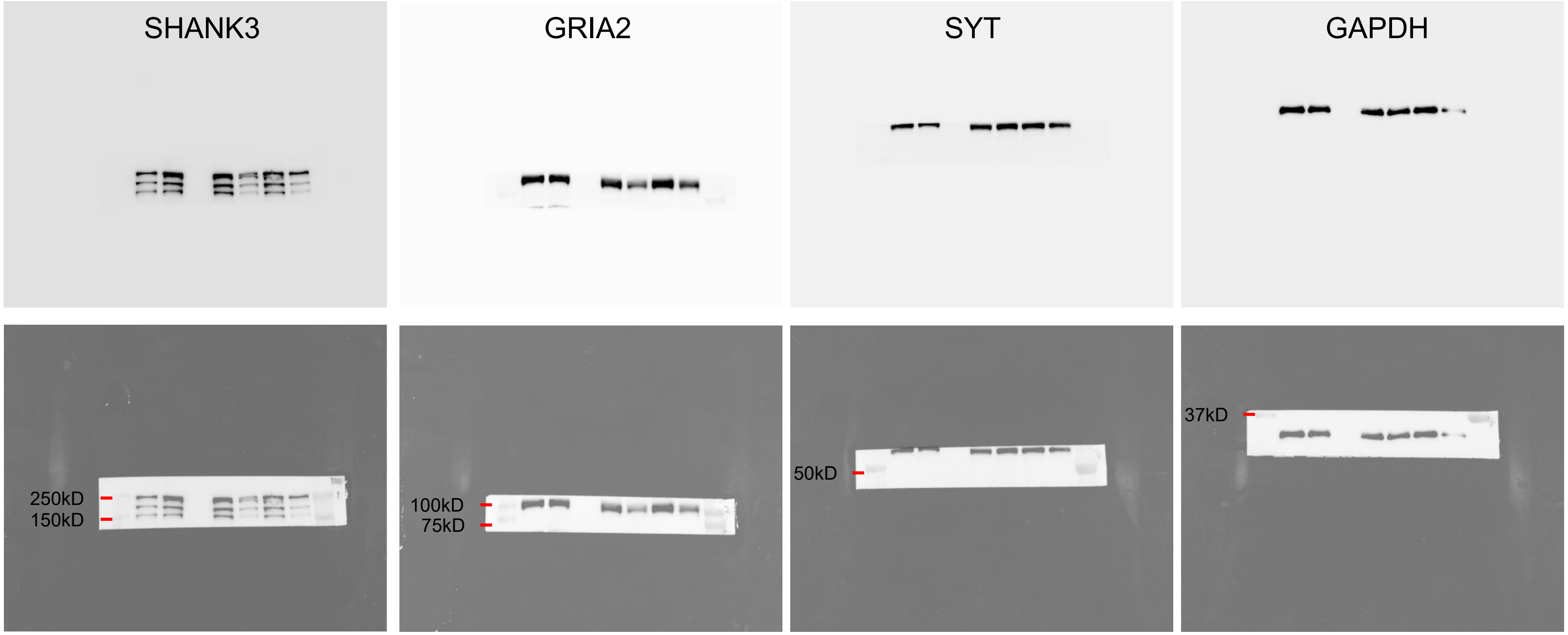

WT6, KI6; X; WT7, KI7; WT8, KI8

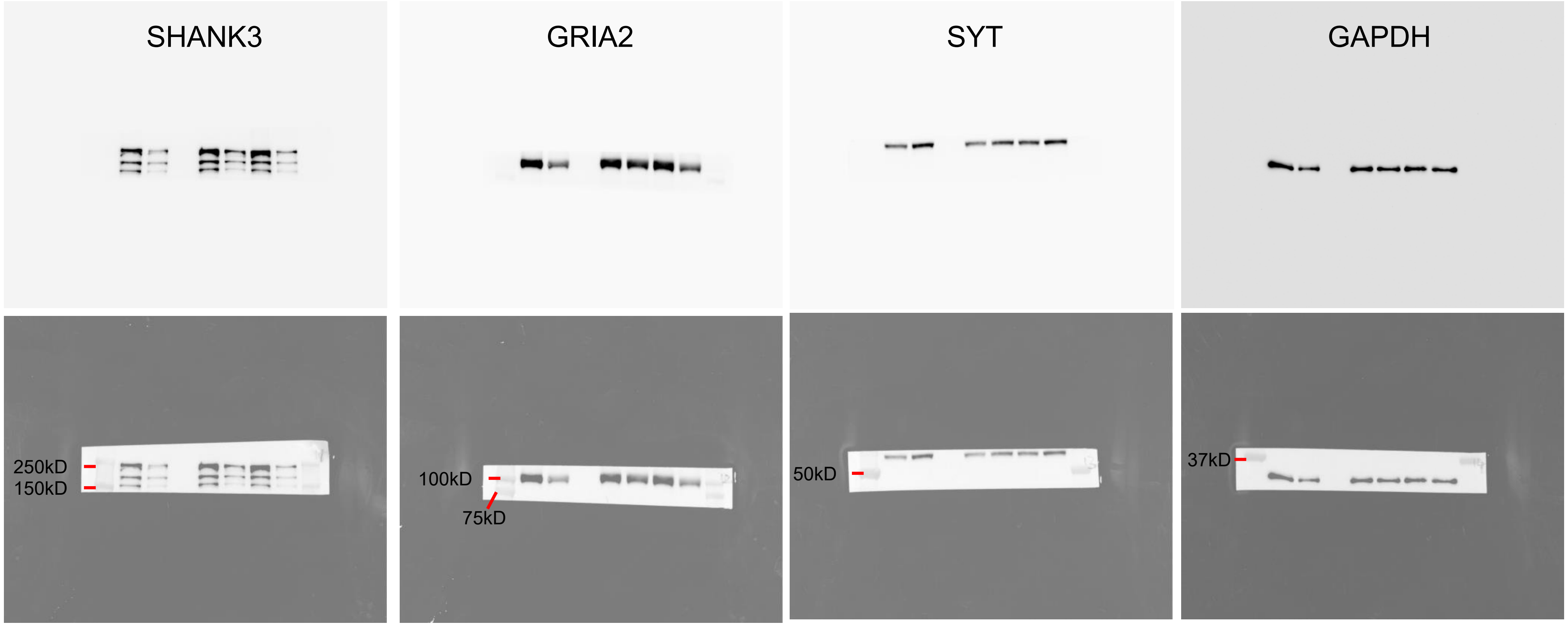

14wk_HIP

Sample loading:
WT1 KI1; WT2, KI2;

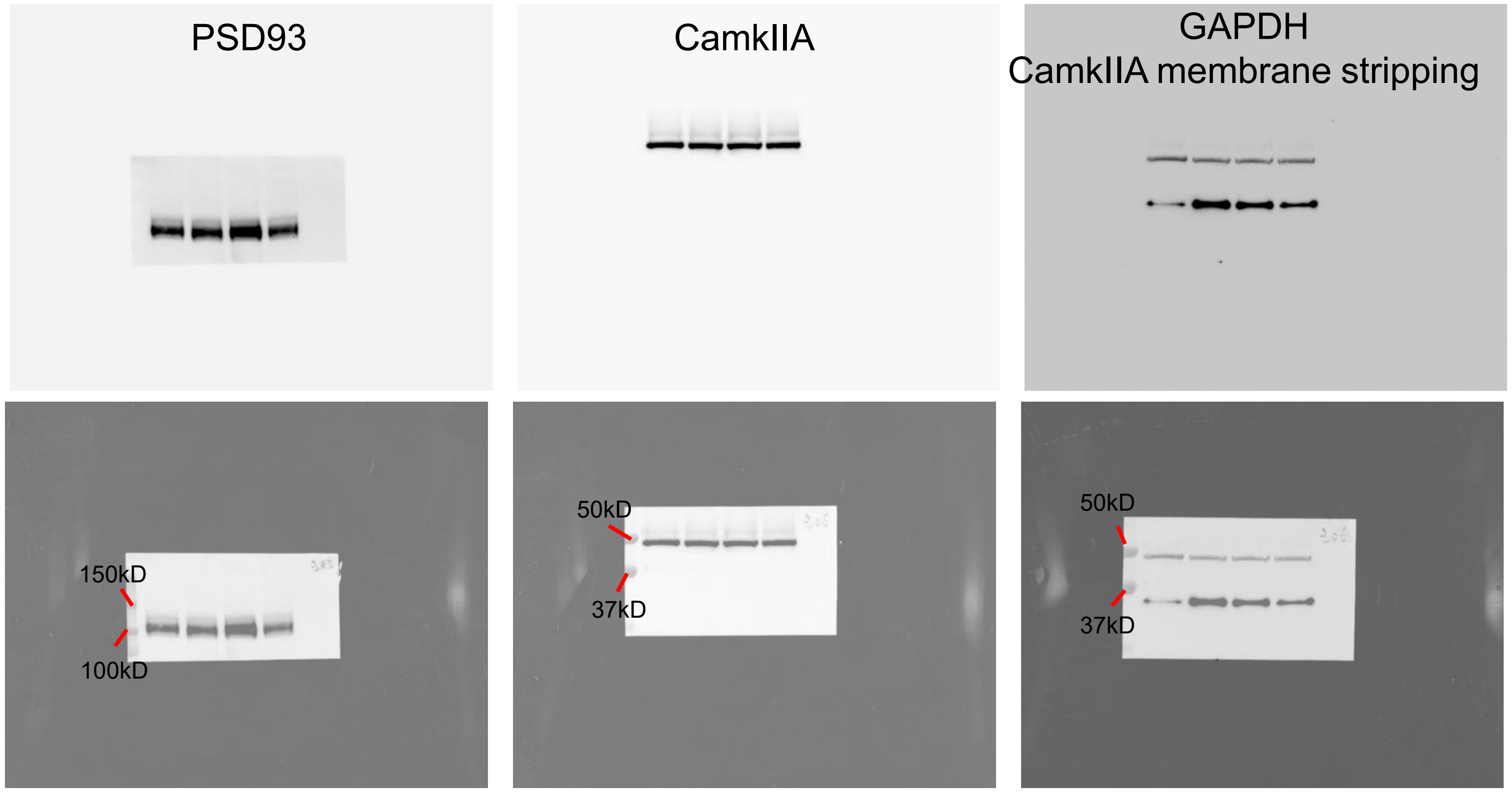

14wk_HIP

Sample loading:
WT3 KI3; X; WT4, KI4; WT5, KI5;

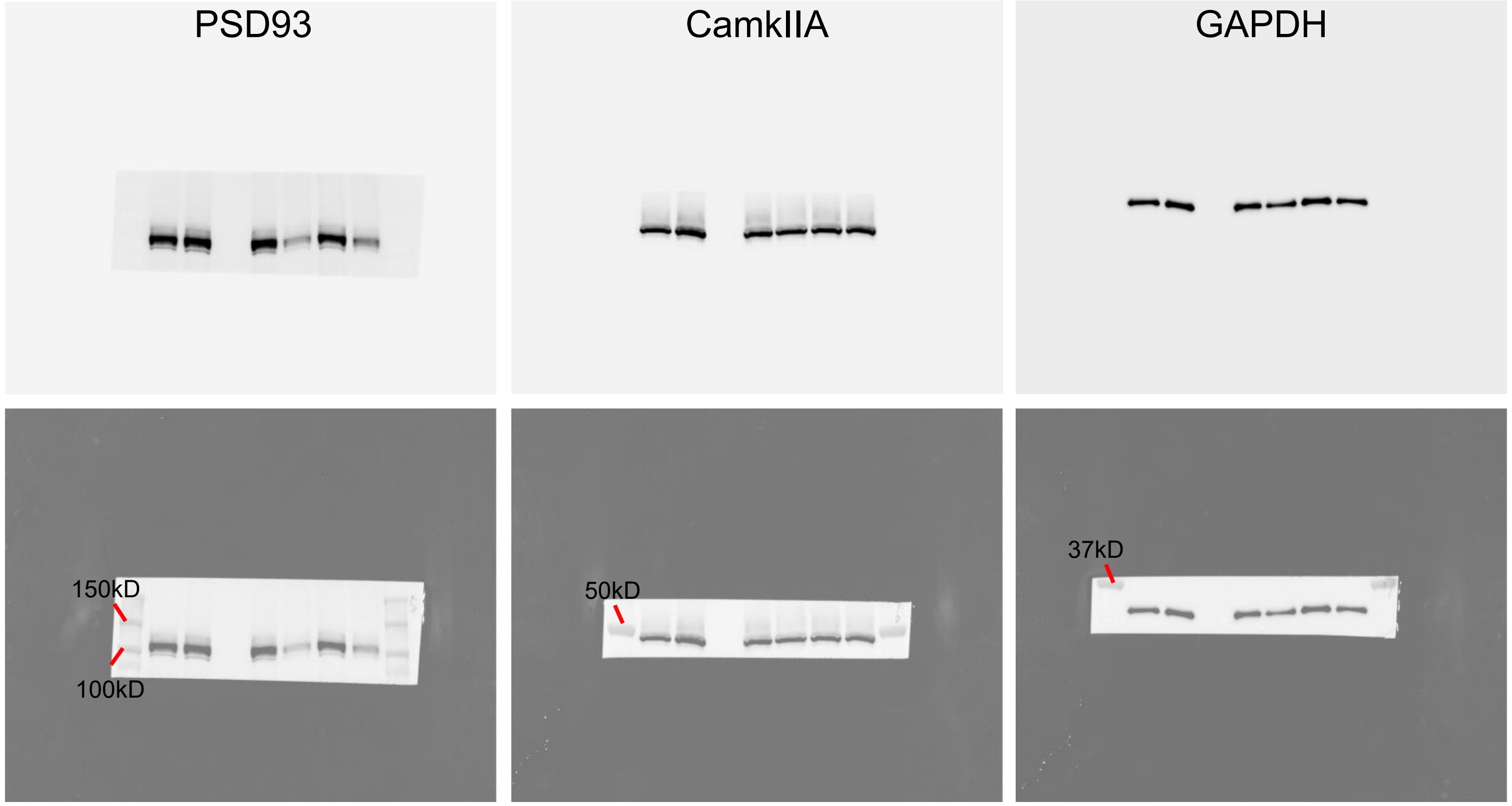

WT6, KI6; X; WT7, KI7; WT8, KI8

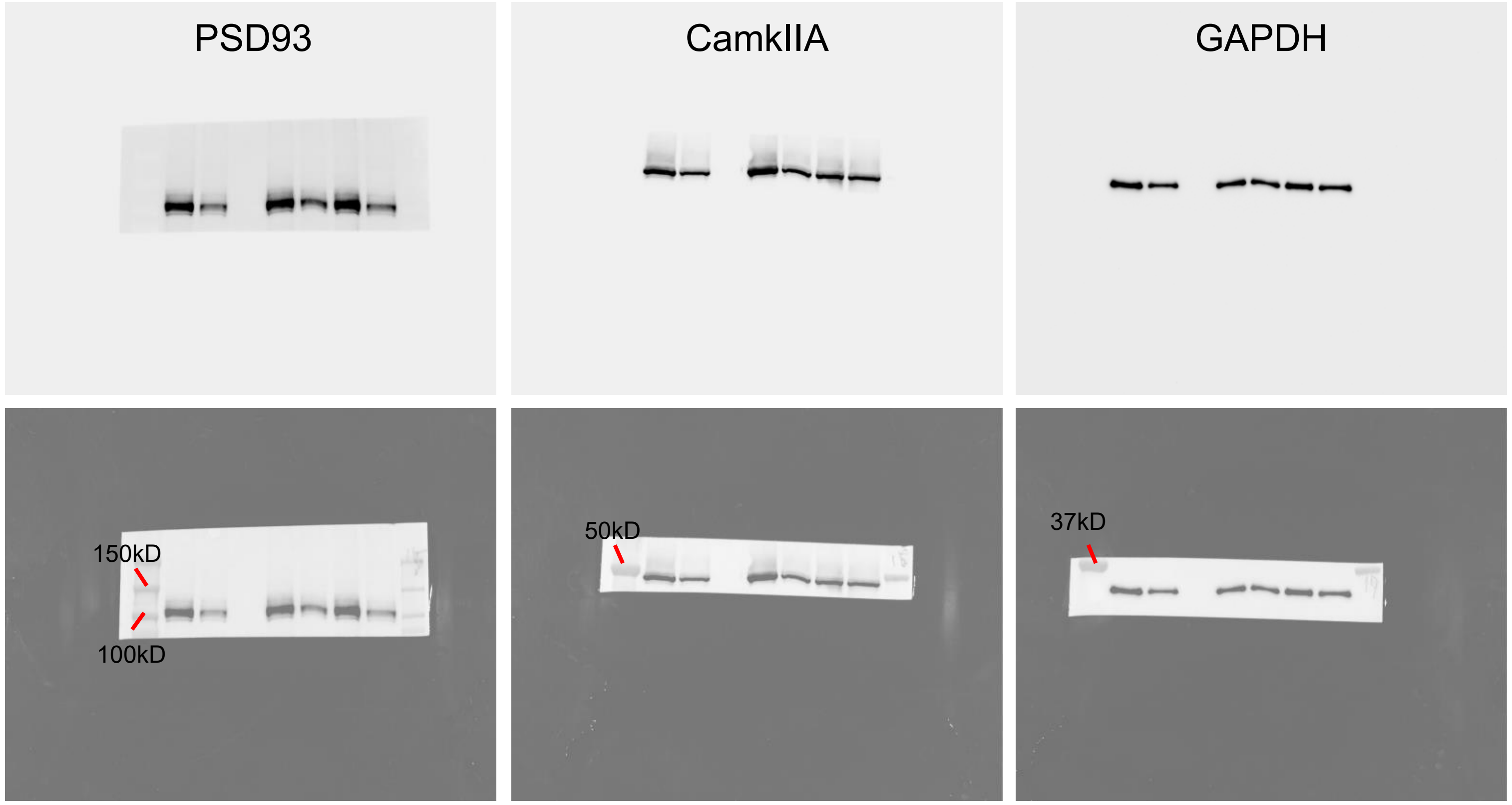

14wk_HIP

Sample loading:
WT1 KI1; WT2, KI2;

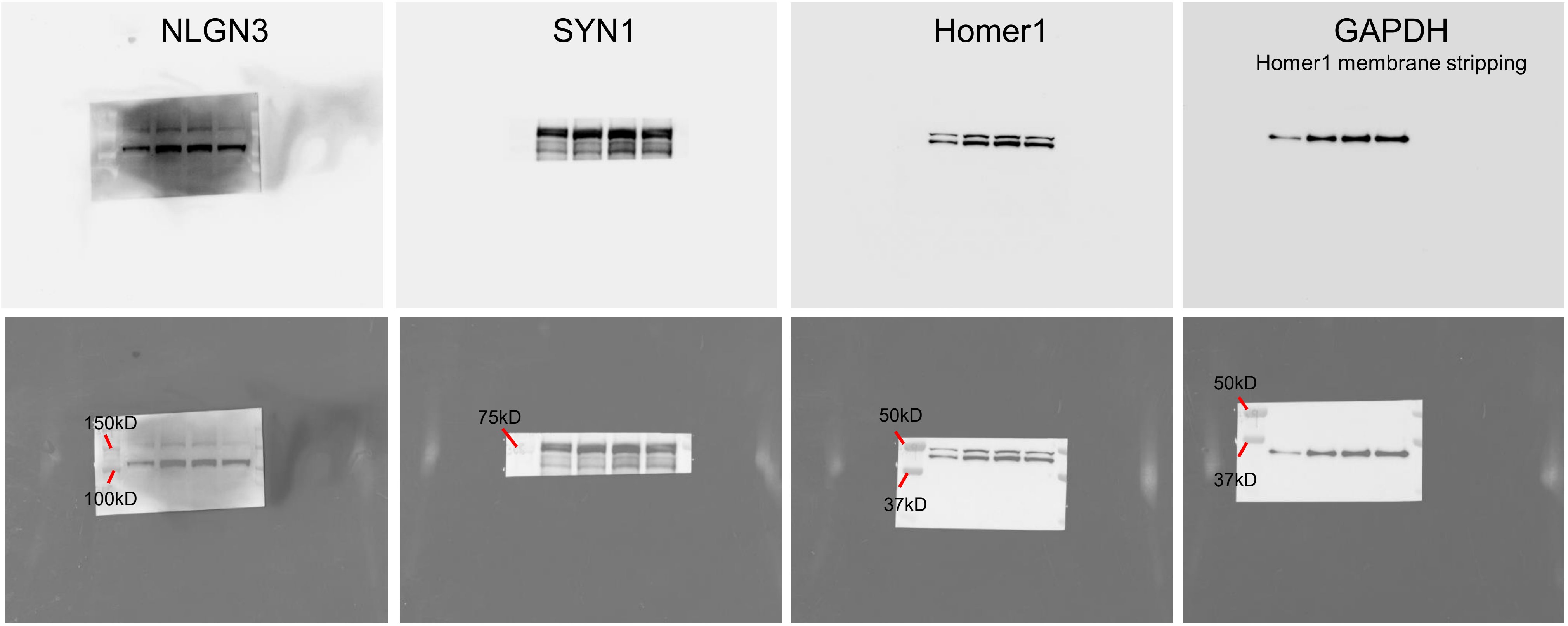

14wk_HIP

Sample loading:
WT3 KI3; X; WT4, KI4; WT5, KI5;

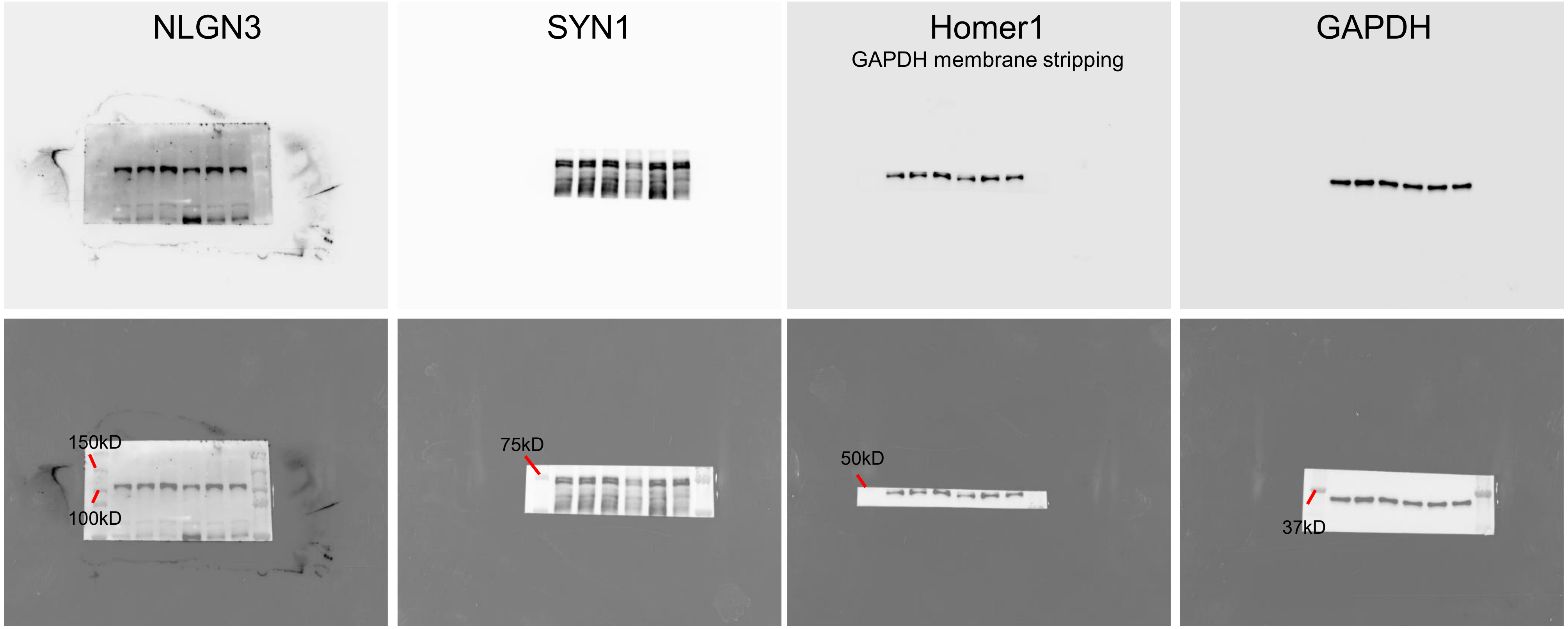

WT6, KI6; X; WT7, KI7; WT8, KI8

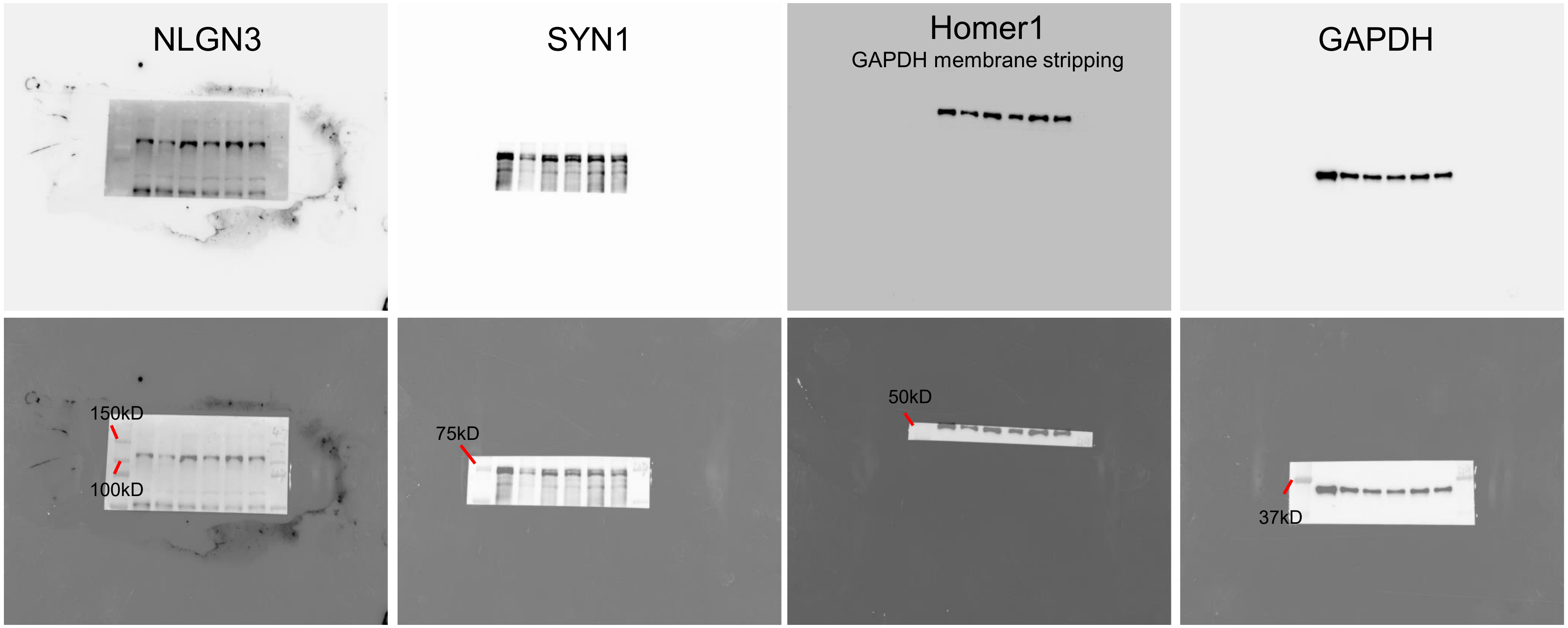

14wk_HIP

Sample loading:
WT1 KI1; WT2, KI2;

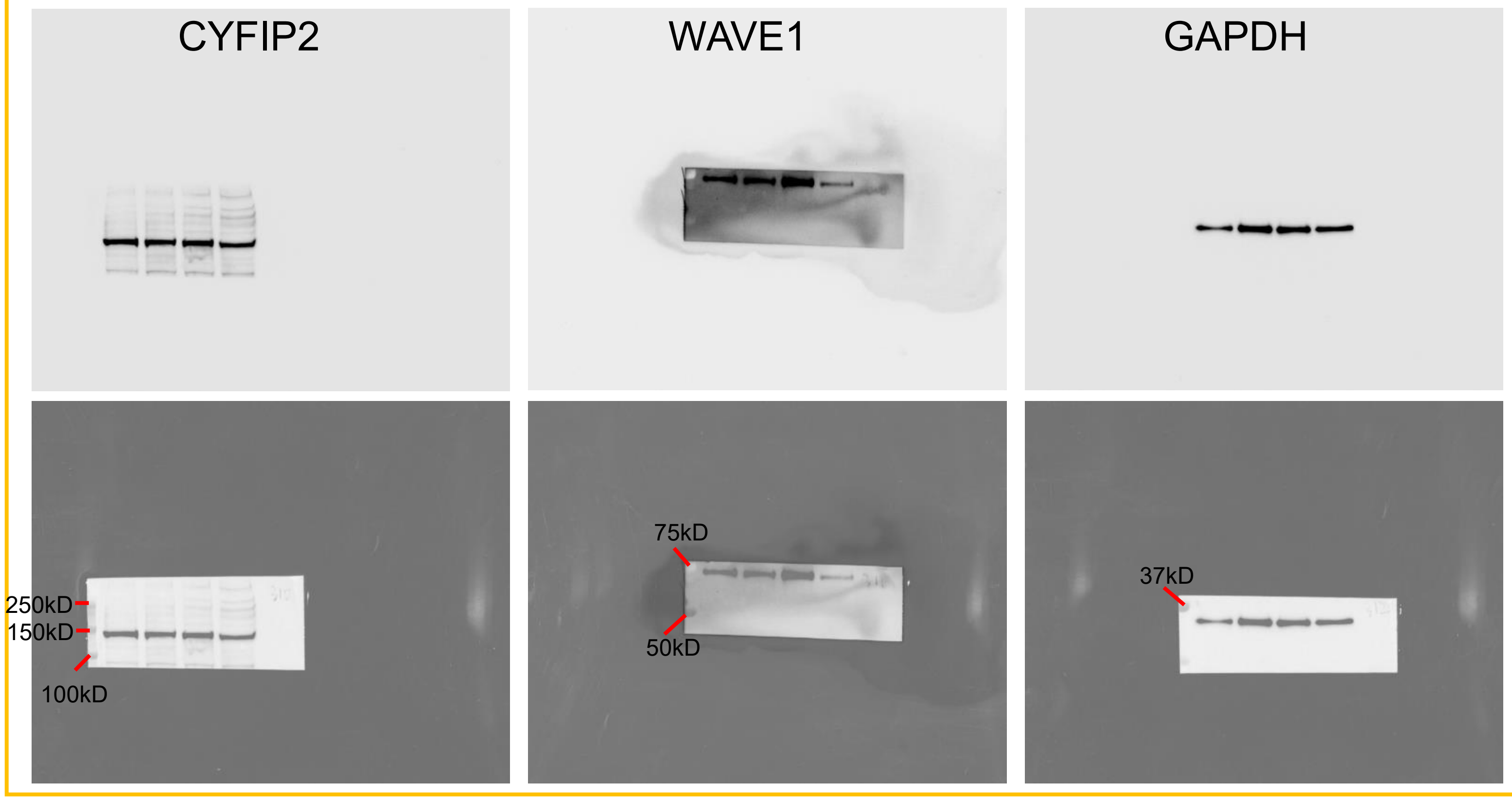

14wk_HIP

Sample loading:

WT3 KI3; X; WT4, KI4; WT5, KI5;

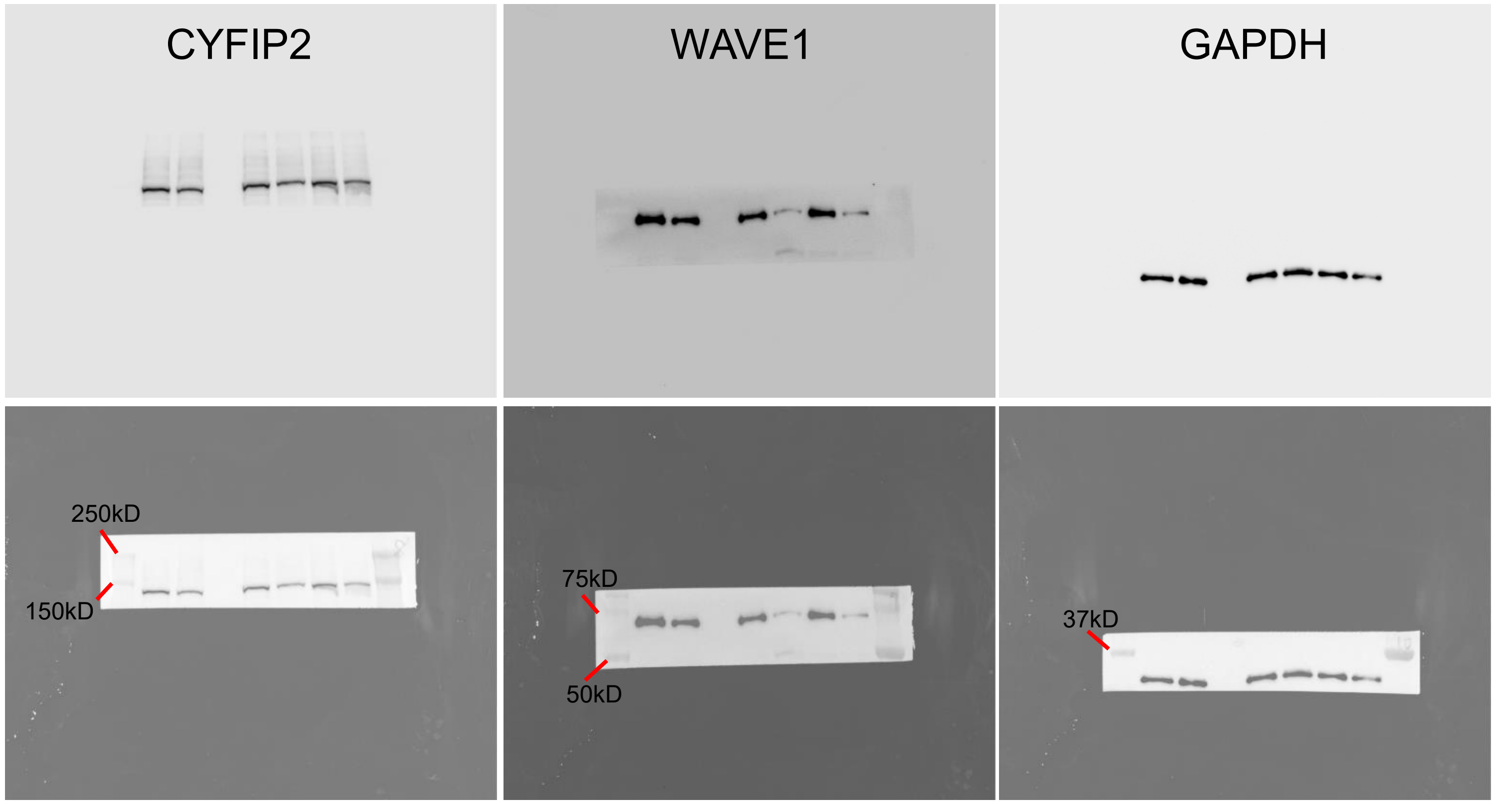

WT6, KI6; X; WT7, KI7; WT8, KI8

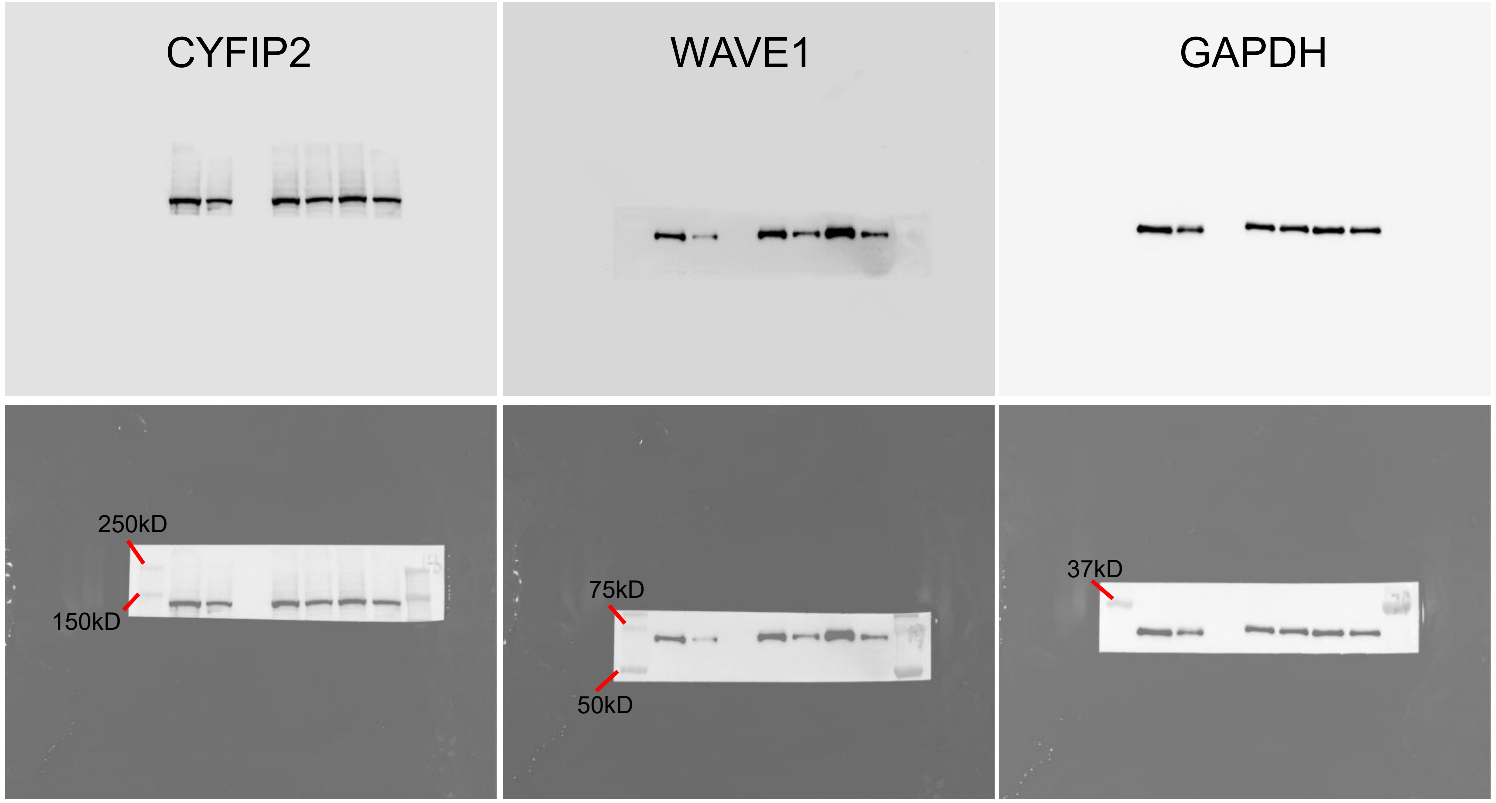

28wk_HIP

Sample loading:
WT1 KI1; WT2, KI2; WT3, KI3; WT4, KI4

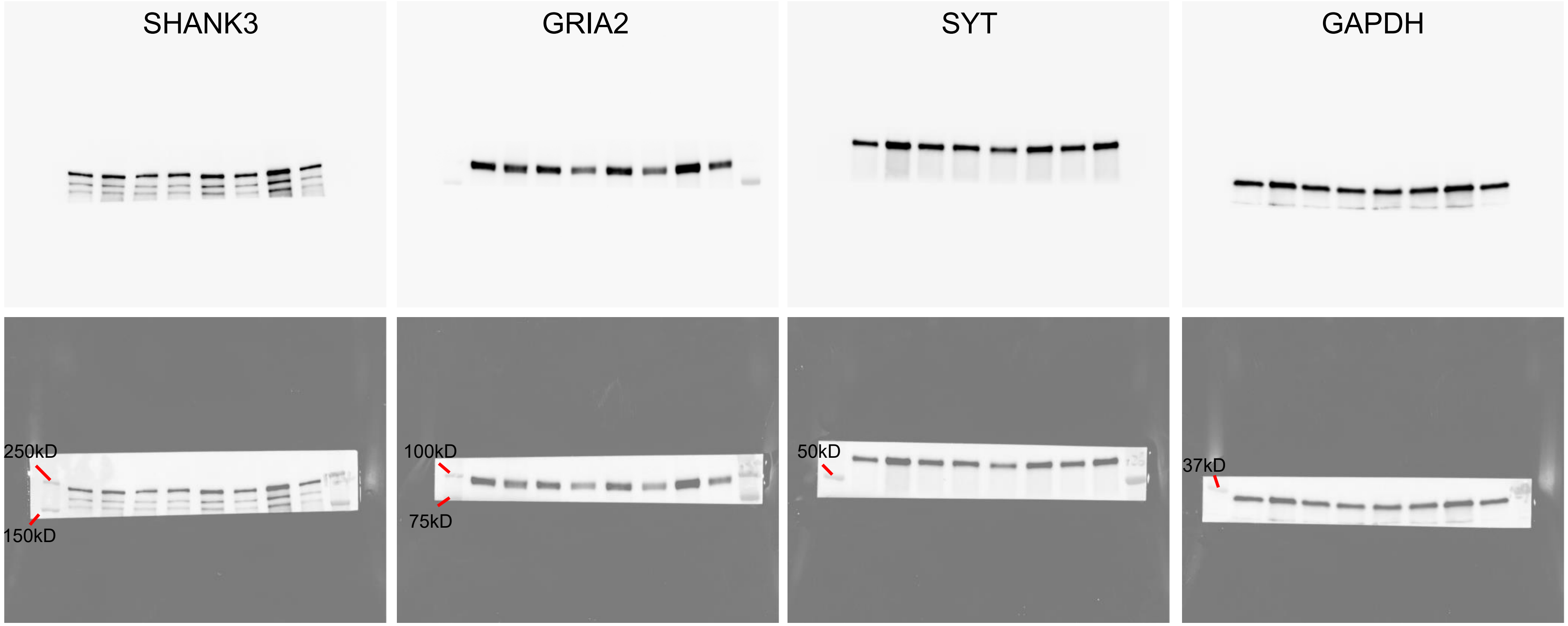

WT5, KI5; WT6, KI6; WT7

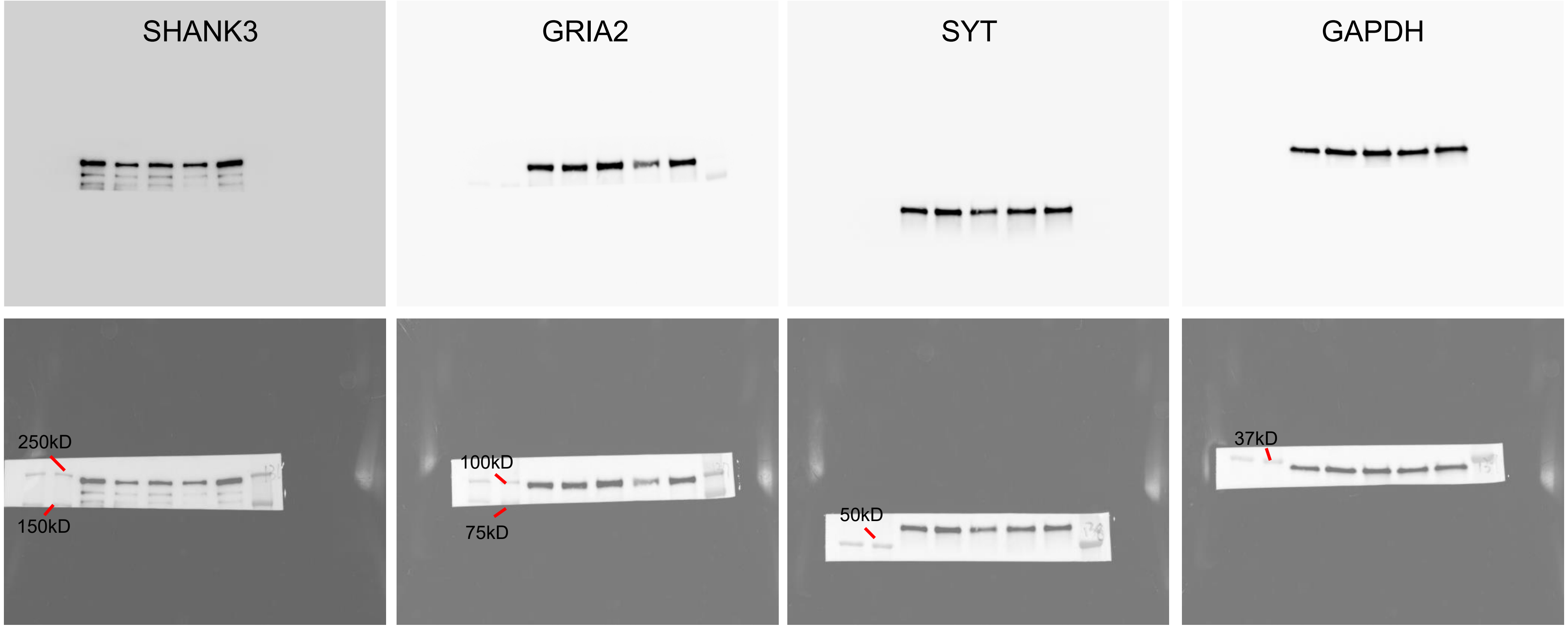

28wk_HIP

Sample loading:
WT1 KI1; WT2, KI2; WT3, KI3; WT4, KI4

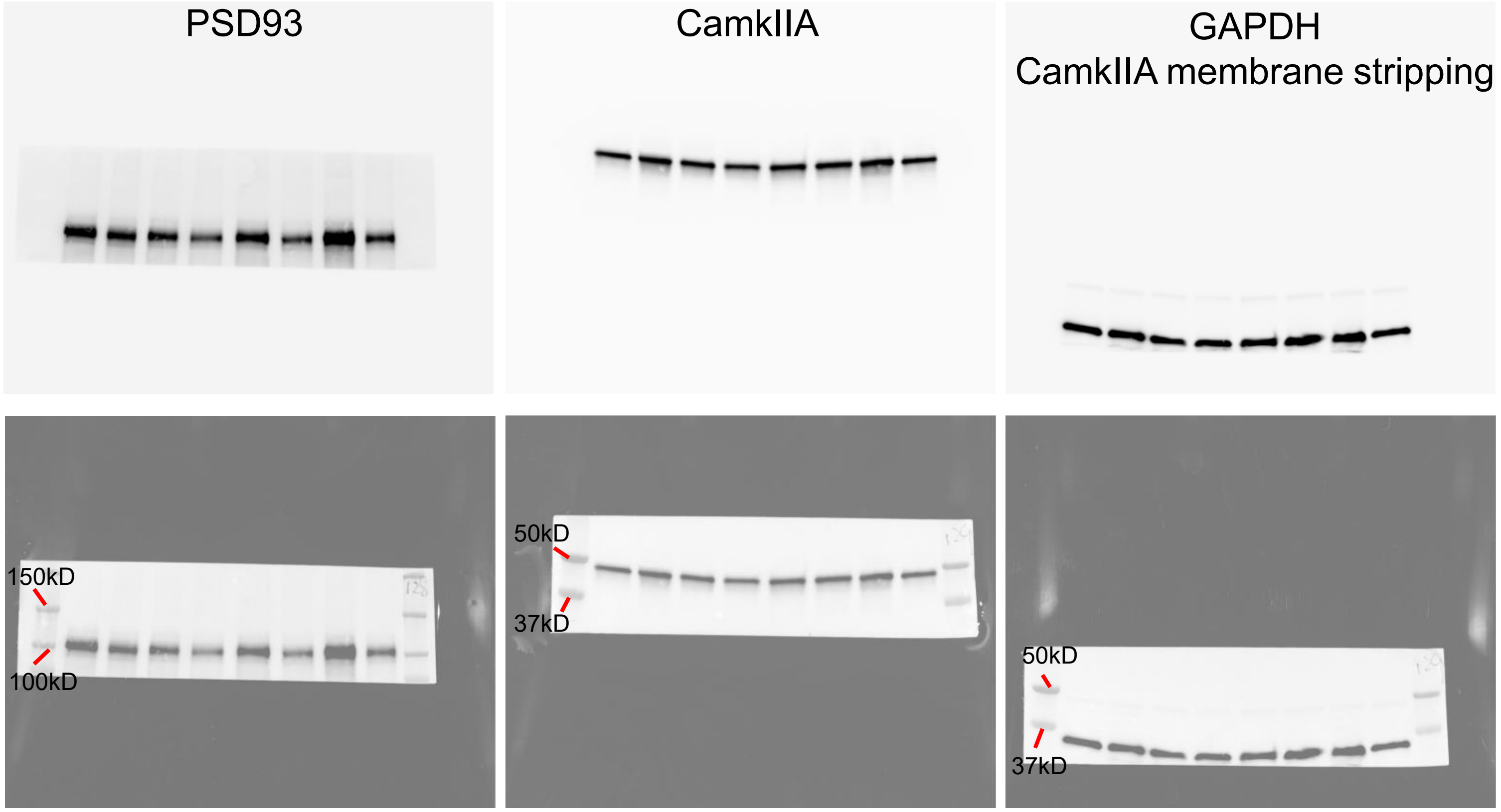

WT5, KI5; WT6, KI6; WT7

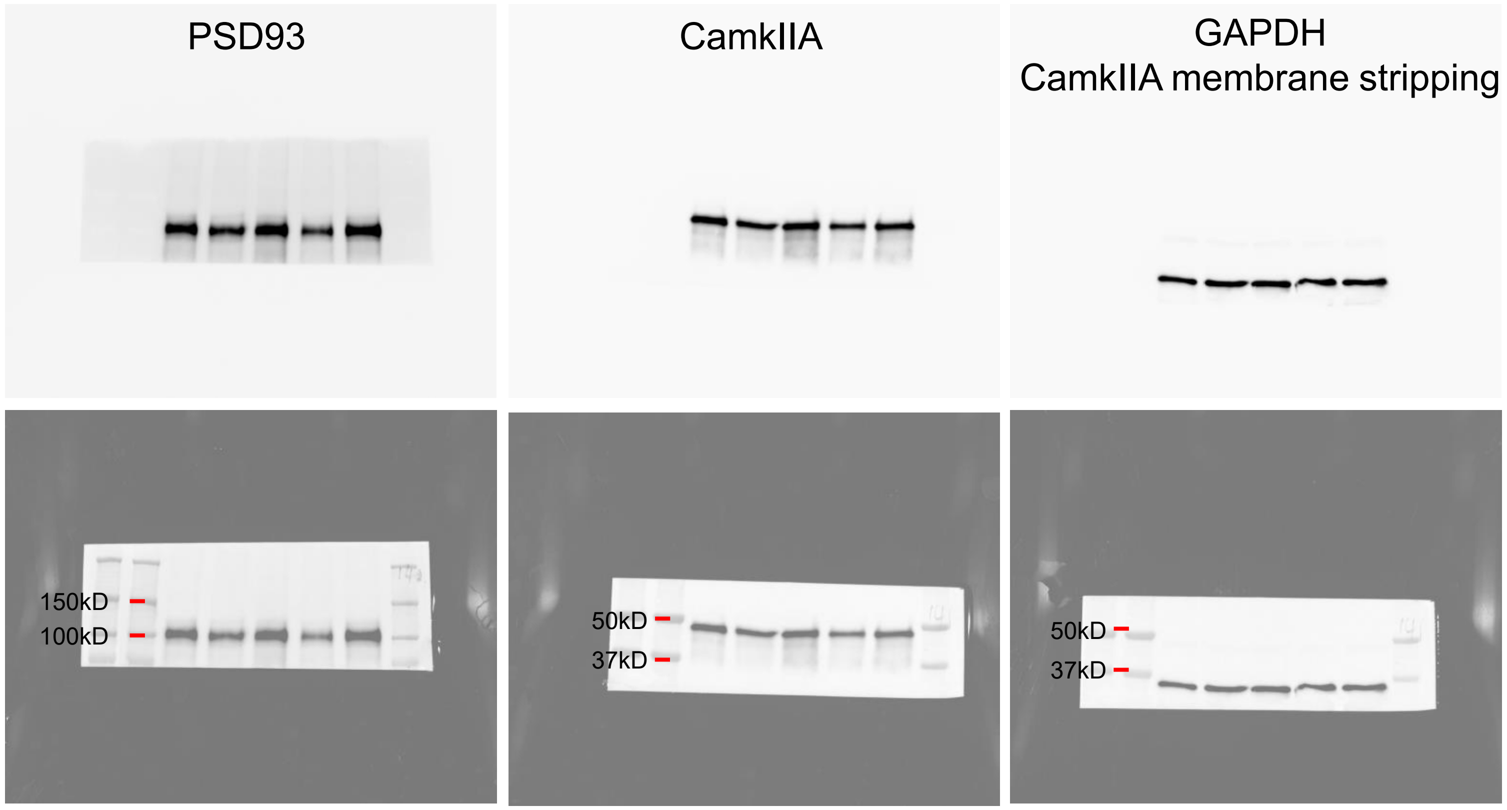

28wk_HIP

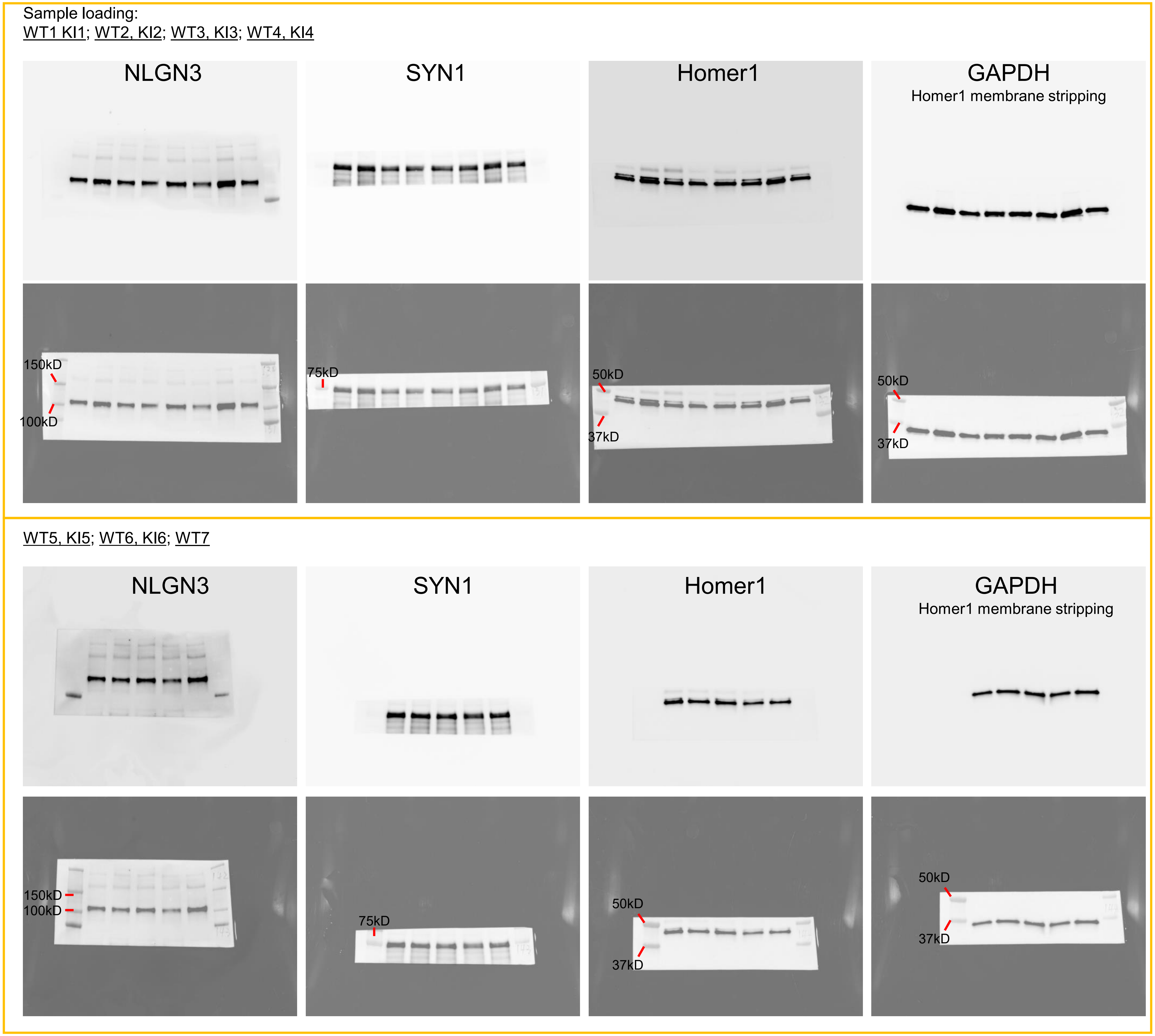
Sample loading:
WT1 KI1; WT2, KI2; WT3, KI3; WT4, KI4
NLGN3
SYN1
Homer1
GAPDH
Homer1 membrane stripping
150kD
100kD
75kD
50kD
37kD
50kD
37kD
WT5, KI5; WT6, KI6; WT7
NLGN3
SYN1
Homer1
GAPDH
Homer1 membrane stripping
150kD
100kD
75kD
50kD
37kD
50kD
37kD

28wk_HIP

Sample loading:
WT1 KI1; WT2, KI2; WT3, KI3; X, KI4

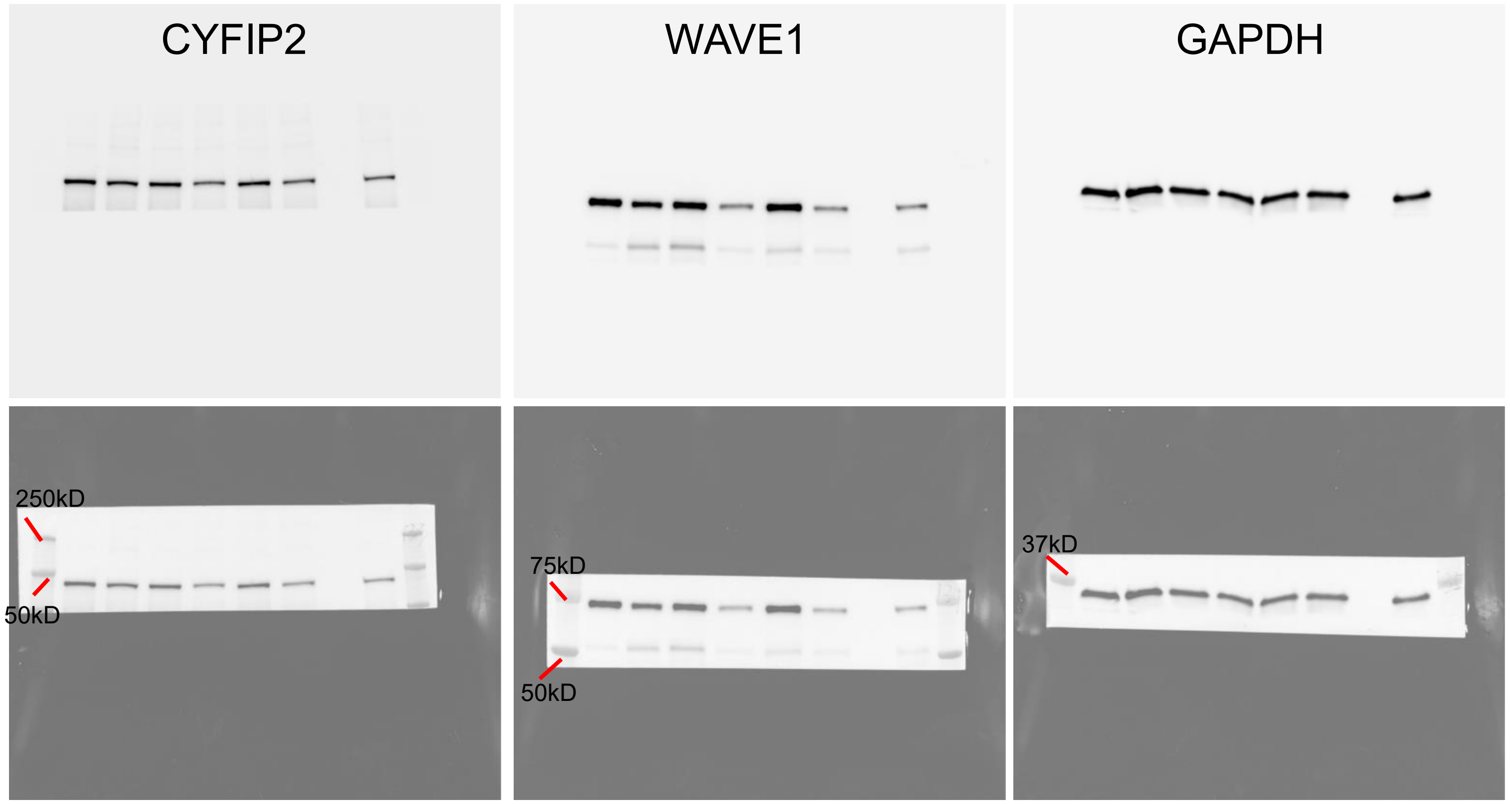

WT5, KI5; WT6, KI6; WT7

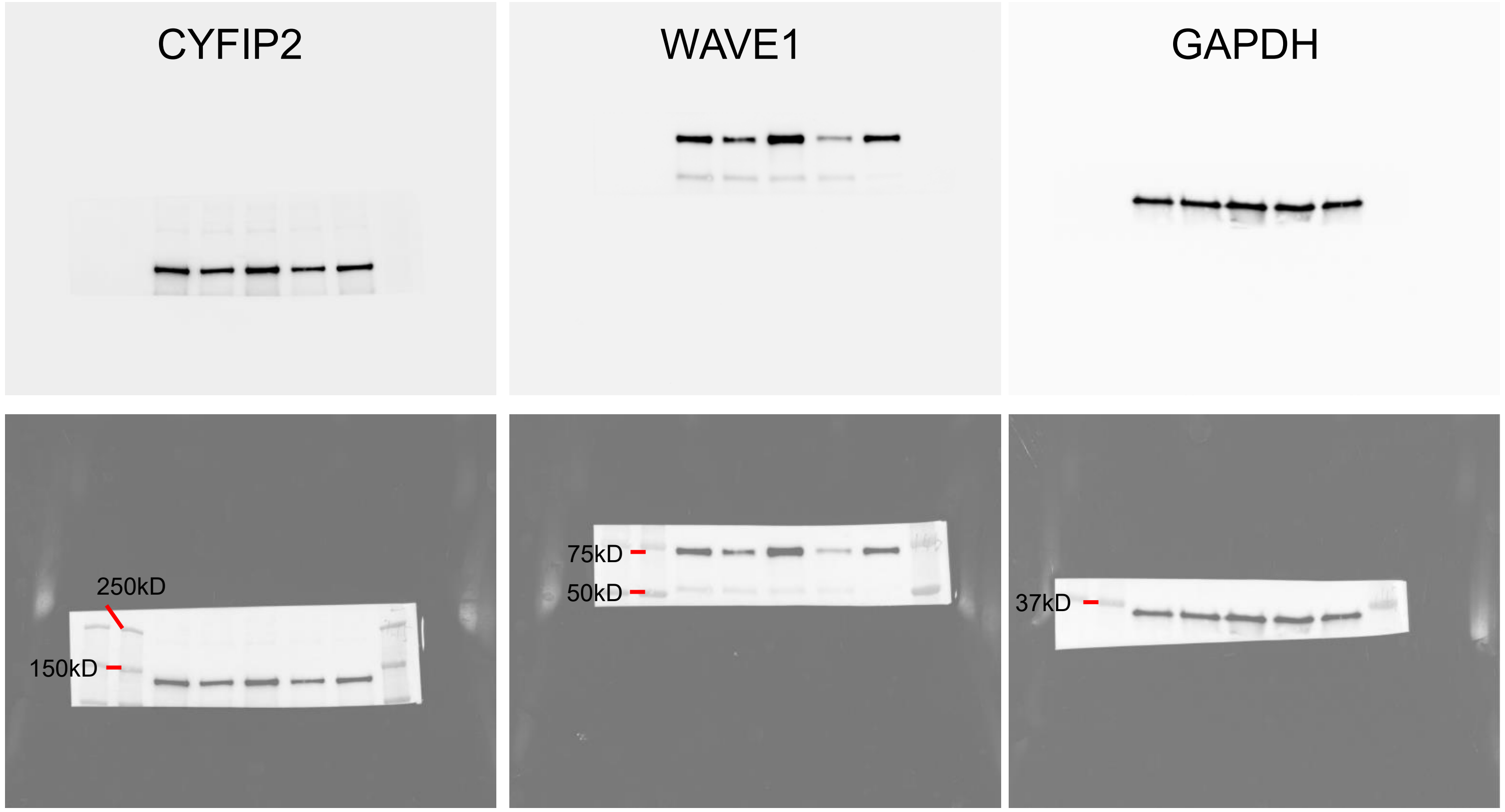

Supplement: S1 Raw Images — (PDF) [file pbio.3003192.s039.pdf]
